# Comprehensive *in silico* analysis of the underutilized crop tef (*Eragrostis tef* (Zucc.) Trotter) genome reveals drought tolerance signatures

**DOI:** 10.1186/s12870-023-04515-1

**Published:** 2023-10-21

**Authors:** Abreham Bekele-Alemu, Ayalew Ligaba-Osena

**Affiliations:** Laboratory of Plant Molecular Biology and Biotechnology, Department of Biology, University of North Carolina Greensboro, Greensboro, NC USA

**Keywords:** Tef, Drought stress, Drought-responsive genes, Functional analysis, *In silico* analysis, Underutilized crop, Gene expression

## Abstract

**Background:**

Tef (*Eragrostis tef*) is a C_4_ plant known for its tiny, nutritious, and gluten-free grains. It contains higher levels of protein, vitamins, and essential minerals like calcium (Ca), iron (Fe), copper (Cu), and zinc (Zn) than common cereals. Tef is cultivated in diverse ecological zones under diverse climatic conditions. Studies have shown that tef has great diversity in withstanding environmental challenges such as drought. Drought is a major abiotic stress severely affecting crop productivity and becoming a bottleneck to global food security. Here, we used *in silico*-based functional genomic analysis to identify drought-responsive genes in tef and validated their expression using quantitative RT-PCR.

**Results:**

We identified about 729 drought-responsive genes so far reported in six crop plants, including rice, wheat, maize, barley, sorghum, pearl millet, and the model plant Arabidopsis, and reported 20 genes having high-level of GO terms related to drought, and significantly enriched in several biological and molecular function categories. These genes were found to play diverse roles, including water and fluid transport, resistance to high salt, cold, and drought stress, abscisic acid (ABA) signaling, de novo DNA methylation, and transcriptional regulation in tef and other crops. Our analysis revealed substantial differences in the conserved domains of some tef genes from well-studied rice orthologs. We further analyzed the expression of sixteen tef orthologs using quantitative RT-PCR in response to PEG-induced osmotic stress.

**Conclusions:**

The findings showed differential regulation of some drought-responsive genes in shoots, roots, or both tissues. Hence, the genes identified in this study may be promising candidates for trait improvement in crops via transgenic or gene-editing technologies.

**Supplementary Information:**

The online version contains supplementary material available at 10.1186/s12870-023-04515-1.

## Introduction

Climate changes and increased water scarcity in some regions challenge global food security and threaten the food supply for the ever-growing global population [1–3]. To feed such a growing population, global agricultural production might need to increase by 60–110% [4, 5]. Field crops continuously experience fluctuations in environmental conditions and are often exposed to abiotic stresses such as drought, salinity, excess light, high/low temperatures, and nutrient imbalances [6, 7]. The capability of plants to respond to abiotic stress is associated with the plasticity and adaptability of their traits to the fluctuating conditions of water availability [8].

Drought is a major abiotic stress that severely affects crop production and productivity [9]. Drought affects nutrient availability, plant growth, and survival [10]. It is a complex phenomenon that can be classified into agricultural, metrological, and hydrological components [11]. A hydrological drought is associated with a deficiency in the water supply volume, a meteorological drought encompasses the degree of dryness and the duration of the dry period, and an agricultural drought results from a shortage of available water for plant growth [11]. Studies indicate that more than 30% of the world’s agricultural land is subjected to drought of which 14% is an extreme one [12, 13].

To adapt to various environmental conditions on earth, plant species have evolved C_3_, C_4_, and Crassulacean Acid Metabolism (CAM) photosynthetic systems [14]. CAM and C_4_ photosynthesis were thought to have evolved from the classical C_3_ photosynthetic pathway around 20–30 million years ago [15]. CAM and C_4_ photosynthetic processes achieve increased water use efficiency by concentrating CO_2_ at the C-fixation site of the dark reactions of photosynthesis [16]. The C_3_ photosynthesis is a one-stage process that produces a three-carbon compound (3-phosphoglyceric acid) via the Calvin Benson-Bassham (CBB) cycle, while C_4_ and CAM photosynthesis are two-stage processes, with the first CO_2_ fixation stage generating a four-carbon compound malate, followed by decarboxylation of malate, releasing CO_2_ to be refixed through the CBB cycle [16]. To boost crop resilience to global warming and to increase crop yields, efforts are ongoing to engineer C_4_ and CAM traits into C_3_ crop species [17–19].

Developing elite crop germplasms is one of the approaches used to mitigate the impact of drought and promote underutilized crop species that have the potential to enhance food security under unfavorable environmental conditions. Exploiting the large gene pool of underutilized crop plants would provide a more diversified agricultural system and an alternative healthy food resource, ensuring food, and nutritional security [20]. However, the world still relies on a limited number of food crops mostly C_3_ cereals like wheat (*Triticum aestivum),* barley *(Hordeum vulgare)* and rice (*Oryza sativa)*, and very few C_4_ cereals such as pearl millet (*Pennisetum glaucum)*, maize* (Zea mays)*, and sorghum (*Sorghum bicolor)* [21]. However, there are still several drought-tolerant and underutilized crops like tef *(E. tef*) that could be an alternative source of food, feed, and energy.

Tef is a tetraploid (2n = 4x = 20) self-pollinated crop [22]. The genus *Eragrostis* comprises about 350 species of which tef is the only species cultivated for human consumption as gluten-free grain [23]. Tef is a staple crop in Ethiopia and Eritrea for about 80 million people [24, 25] where it is most widely produced. In Ethiopia, tef was produced on about three million hectares of land in the 2021/22 cropping season and accounted for a yield estimate of about 5.7 million metric tons [26]. Most accessions of tef including their wild relatives grow under a wide range of ecological conditions, ranging from sea level to 3000 m above sea level (m.a.s.l) [27, 28]. Over the last few decades, domestication, and cultivation of tef has been taking place in several other countries including South Africa, Australia, India, USA, China, Netherlands, and Israel for its healthy grains as well as forage grass [27, 28]. Studies have shown that tef is high in protein, vitamins, and essential minerals like calcium, iron, copper, and zinc as compared to other cereal grains such as wheat, maize, barley, and sorghum [29–32] and becoming globally popular due to its attractive nutritional profiles.

Despite tef’s potential as a nutritious and healthy crop, its productivity is limited due to various factors including the lack of modern farming technologies, susceptibility to lodging (permanent bending of the stem from the upright position), soil acidity, salinity, and terminal drought [33]. Tef is an ‘orphan crops’ that has not benefitted from genetic improvement programs [31]. Its yield also remained very low with a national average yield below 1.75 t/ha in Ethiopia [34]. Tef seed is one of the smallest grains in the world with a length of about 1.0 mm and a width of about 0.60 mm [25]. Tef is moderately drought tolerant when compared to its wild progenitor *Eragrostis pilosa* [25]. It is reported that the yield loss due to moderate to severe drought from booting to grain filling stages was 35%—52% [35, 36]. A yield reduction of 69 to 77% has also been documented due to drought at the anthesis stage [37]. In Ethiopia, several germplasm screening projects were conducted and some promising tef varieties have been identified [38–42]. Furthermore, seven differentially expressed miRNAs linked to drought tolerance in tef were reported [43]. An attempt to improve drought tolerance using ethyl methyl sulfonate (EMS) based chemical mutagenesis [44] generated two early drought-tolerant (*dtt2* and *dtt13*) and three terminal drought-tolerant lines (*tdt9, tdt15,* and *tdt19*) tef varieties that have potential for trait improvement through breeding. Hence, a comprehensive understanding of the impact of drought and associated stresses is critical for developing climate-resilient crops that can adapt to changing climatic conditions.

As more than 1000 whole genome sequence data are available comprising about 788 plant species in the last two decades [45], *in silico* analysis and identification of candidate genes implicated in several agronomic traits, including drought, is becoming handier. Similarly, several bioinformatics tools capable of analyzing the role of genes and gene products are becoming available. In recent years, many research articles are utilizing *in silico* analysis for gene identification because it is cost-effective, fast, and does not need sophisticated equipment. *In silico* analysis is having a great impact on shortening the lengthy classical and laborious wet lab experimentation. It has been used to identify differentially regulated drought-responsive genes in a number of plant samples [46–48]. As most studies conducted so far were based on the identification of one or a few genes, attempts to identify a large array of genes from the whole transcriptome are limited. For instance, despite the availability of the draft genome sequence of tef, *in silico* gene identification so far focused on a few selected transcriptional factors (TFs) rather than utilizing a large array of drought-responsive genes from a range of plant species [49–54]. Some research articles published in the last two years on *in silico* analysis of drought-responsive gene identification in different crops are summarized in Table 1 below. In this paper, were performed *in silico* analyses and identified 20 potential candidate drought-responsive genes in the tef genome based on ortholog genes reported in related grass species and the model plant *Arabidopsis thaliana*. Furthermore, quantitative qPCR was used to validate the expression of putative drought-responsive genes in tef.

**Table 1:** Recent activities on *in silico* drought responsive gene identification

SN	Activities conducted	Gene identified	Reference
1	Genome-wide *in silico* identification and characterization of the stress associated protein (SAP) gene family encoding A20/AN1 in potato	17 *StSAP* genes	[55]
2	Genome-wide *in silico* identification of phospholipase D (PLD) gene family from Corchorus capsularis and *Corchorus olitorius*	12 and 11 *PLD* genes in the genome of *C*. *capsularis* and *C*. *olitorius*, respectively	[56]
3	Identification of candidate genes regulating drought tolerance in pearl millet	74 drought-responsive genes separated into five phylogenic groups	[57]
4	*In-Silico* study of Brassinosteroid (BR) signaling genes in rice	39 BR signaling genes	[58]
5	Genome-wide *in silico* identification and characterization of sodium-proton (Na + /H +) antiporters in Indica rice	sixteen *NHX* orthologous	[59]
6	*In silico* identification and annotation of drought responsive candidate genes in *Solanaceous*	109 drought responsive unigenes	[60]
7	*In silico* identification of Rare Cold Inducible 2 (RCI2) gene family in cucumber	Four *RCI2* genes	
8	*In silico* identification and expression analysis of nuclear factor Y (Nf-Y) in cucumber	27 *CsaNF-Y* members	[61]
9	Genome-wide *In silico* identification and comparative analysis of *Dof* gene family in *Brassica napus*	117 *Brassica napus Dof* genes (*BnaDofs*)	[62]
10	Genome-wide identification and *in silico* analysis of nitrate transporters in hexaploid wheat	412 nitrate transporter genes	[63]
11	*VOZS* identification from tef [*Eragrostis tef* (Zucc.) Trotter] using *in silico* tools	Four *VOZs* from tef	[50]
12	Genome-wide investigation of defensin genes in peanut	12 *AhDef* genes	[64]
13	Comparative *in silico* analysis of Eragrostis tef with other species for elucidating presence of growth regulating factors (GRFs)	Two conserved genes	[54]
14	Distribution and abundance of *CRE*s in the promoters depicts crosstalk by WRKYs in Tef	180 *CRE*s	[53]
15	Study of HRT-like genes in *Eragrostistef* and *a*nalysis for potential functions	Two *HRT*-like TFs	[49]
16	Identification and characterization of *Dof* in Tef using *in silico* approaches	33 *Dof* TFs	[51]
17	*In Silico* approach for unraveling the structural and functional roles of NF-X1-Like proteins in underutilized cereal tef	four *NFX*-like genes	[52]
18	*In-silico* prediction of novel genes responsive to drought and salinity stress tolerance in bread wheat	22 putative drought- and salinity-related genes	[27]

## Methods

### Identification of the drought-responsive genetic elements

To identify drought-responsive coding and regulatory elements in tef, sequences were retrieved were retrieved from two databases: Drought Stress Gene Database [65] and CrealESTDb which is a resource for abiotic stress-responsive annotated ESTs [63]. Furthermore, we retrieved 175 previously reported drought-responsive genes in tef [66] from the NCBI database. We also downloaded 889 novel abscisic acid, stress, and ripening (ASR) EST recently reported in pearl millet that were isolated from drought stress-responsive suppression subtractive hybridization (SSH library) and reported to confer multiple abiotic stress tolerance in transgenic Arabidopsis [67]. For drought-responsive genes, the nucleotide sequences were retrieved from the NCBI. Overall, drought-responsive genes that were reported in eight plant species, including tef using rice microarray, Arabidopsis, maize, sorghum, barley, wheat, and pearl millet, were used in our *in silico* analysis.

### Mapping of drought-responsive ortholog genes in the tef genome

**Fig. 1:**
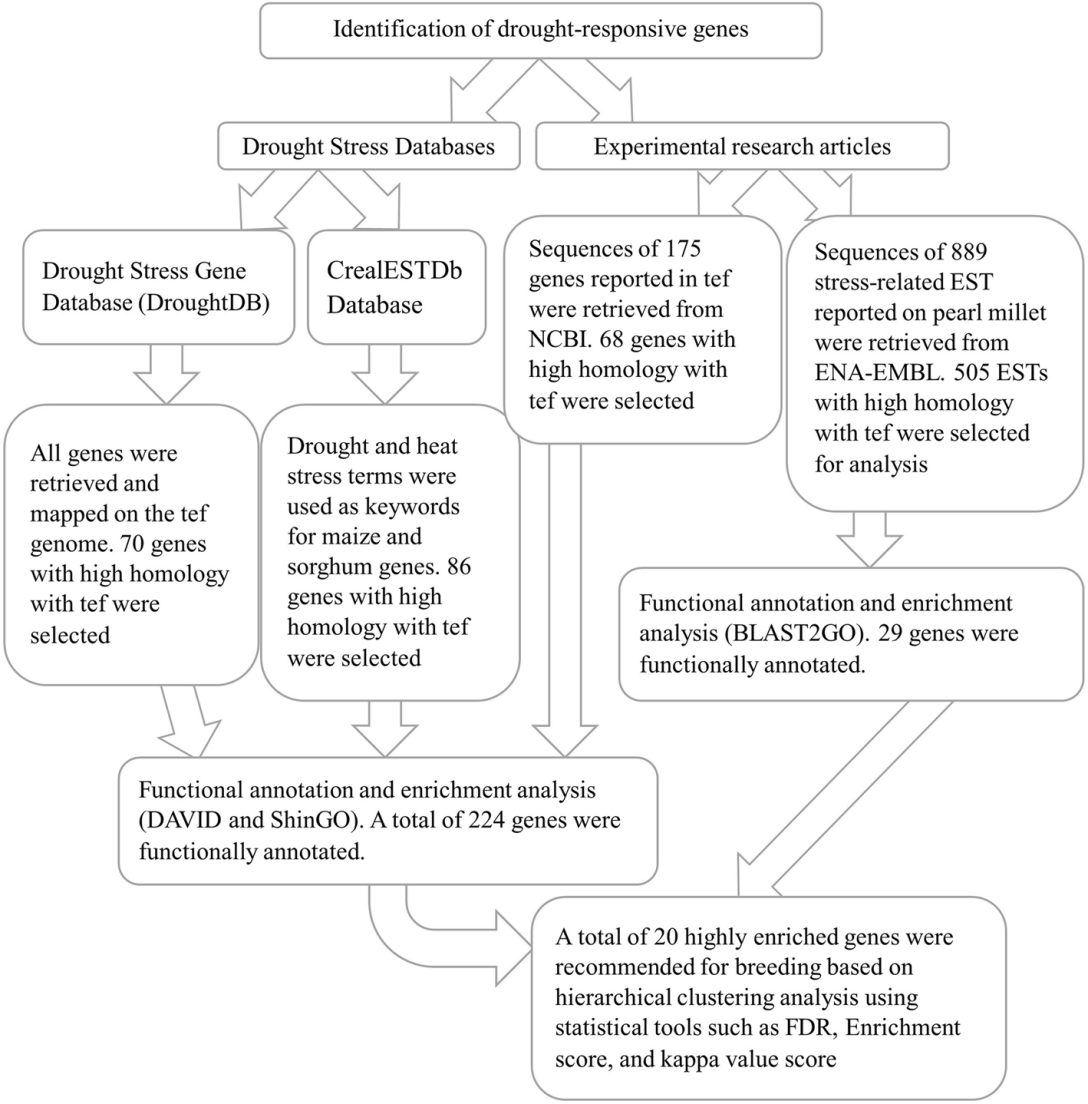
An overall flowchart illustrating the steps from retrieval to gene identification of enriched genes with high level GO terms

To identify drought-responsive gene signatures in the tef genome, we used CoGeBLAST (https://genomevolution.org/coge/CoGeBlast.pl) with genome ID 50954 [68]. Before the homology search, the E-value in CoGe BLAST was set to 1E^−30^ to generate alignment with strong matches and to minimize the inclusion of sequences with low homology. Using drought-responsive genes from the Drought Stress Gene Database [65], 70 genes having strong homology (E-value < 1E^−30^) in the tef genome were selected for further analysis. Using the sorghum and maize drought-tolerant genes deposited in CrealESTDb, 86 gene signatures with strong matches in the tef genome were also selected for further analysis. Moreover, out of 175 genes previously reported in tef, 68 genes with top hits were selected. European Nucleotide Archive-European Molecular Biology Language (ENA-EMBL) contained 889 pearl millet EST database [67] of which 505 EST having strong homology with the tef genome were also used for further analysis (Supplementary Table 1). In total, 729 genes and gene elements were used in the analysis. Figure 1 illustrates the overall flowchart from gene retrieval to the identification of highly enriched genes with high-level GO terms and their potential use in future breeding programs.

### Genetic relatedness of drought-responsive genes and genes with high-level GO term

High-level gene ontology terms represent highly enriched genes in three categories: Biological Process (BP), Molecular Function (MF), and Cellular Component (CC). To determine the phylogenetic relationship between the genes, we used 233 sequences that were identified in the *E. tef* genome. Multiple sequence alignment was performed using CLUSTALW, and the phylogenetic tree was constructed using the Neighbor Joining method [69]. The text tree file was then imported to Interactive Tree Of Life (iTOL) v5 [70] for phylogenetic tree display and annotation. For the genetic relationship of top genes, we used CDS version of homologous sequences from rice, finger millet (*Eleusine coracana)*, and foxtail millet (*Setaria italica)*. Finger millet and foxtail millet are C_4_ drought-tolerant plants like tef while rice is a C_3_ crop.

### Functional annotation and enrichment analysis of drought-responsive genes

For functional annotation and enrichment analysis, we first converted all sequences to Entrez ID and used Database for Annotation, Visualization, and Integrated Discovery (DAVID) [71], ShinyGO 0.76 [72], and the latest BLAST2GO version of OmicsBox2.1.14 software [73]. Functional annotation of 224 genes was performed by DAVID and ShinyGO while the 505 raw ESTs were functionally annotated using BLAST2GO.

### Clustering of genes, pathway analysis, and protein interaction network analysis

For extracting and clustering of top genes, FDR (false discovery rate; FDR < 0.05) score, enrichment score, *p*-value, and kappa coefficient were used. Using the kappa coefficient, highly enriched genes were reclassified into very high, high, and moderate enrichment categories. The KEGG analysis was used to identify major pathways that are regulated by the drought-responsive genes. The protein–protein interaction (PPIs) networks of genes with high-level GO terms were computed according to Ge et al. [72].

### Identification of conserved domains in genes with high-level GO term

To identify conserved domains in genes with high-level GO terms, we downloaded homologs of rice and foxtail millet from NCBI (https://www.ncbi.nlm.nih.gov/) and finger millet from Phytozome 13 ( https://phytozome-next.jgi.doe.gov/). Foxtail millet and finger millet are C4 grasses closely related to tef, whilst rice is a well-studied C_3_ species. The CDS version of all the genes were predicted by FGENESH online tool (http://www.softberry.com). Conserved domains of the CDS were identified using NCBI conserved domain identification tool [74].

### Drought treatment and gene expression analysis of selected candidate genes

To analyze the expression of drought responsive in tef, hydroponics experiment was conducted using the reference cultivar Dabi obtained from U.S. Germplasm Resources Information Network (GRIN). Briefly, 50 seeds were first washed with autoclaved millipore water in 1.5 microcentrifuge tubes. After the water was removed, the seeds were surface sterilized using 50% bleach for 8 min under continuous agitation. The bleach solution was removed, and the seeds were rinsed four times using autoclaved millipore water. Seeds were then germinated on moist filter paper for five days and the seedlings were transferred to ¼ Hoagland solution for six more days. Eleven-day-old seedling were then assigned to fresh ¼ Hoagland solution with or without 20% PEG8000 (Phytotechnology Laboratories, Lenexa, USA) which was optimized for this experiment. The PEG solution was used to impose osmotic stress in the hydroponic solution at pH 5.6. For the control samples, only ¼ Hoagland solution without PEG was used throughout the experiment. Seedlings were harvested 30 h after PEG treatment; root and shoots were separated; plant tissues were frozen in liquid nitrogen at harvest and stored in -80 °C until use. Total RNA was extracted using GeneJET Plant RNA Purification Mini Kit (Thermo Scientific). cDNA was synthesized using High-Capacity cDNA Reverse Transcription Kit (Applied Biosystems™).

For gene expression analysis using quantitative qPCR, primers were designed for sixteen candidate transcripts (*EtWOX9, EtZIP1, EtbZIP23, EtNAC2, EtDREB1A, ETDREB1C, EtDREB2A, EtPIP1-1, EtPIP1-3, EtPIP2-2, EtCPK21, EtNRT1, EtSAP8*, *EtMATE1, EtDRM3* and *EtCPP1*) using primer3plus (https://www.primer3plus.com/) (Table 2). The qPCR was performed by QuantStudio3 (Applied Biosystems) using 1X PowerUp SYBR Green master mix, 0.5 µM of forward and reverse primers and 10 ng of cDNA (1 µl) in a total volume of 20 µl. The PCR condition was 2 min initial denaturation at 95 °C, 15 s denaturation at 95 °C, 30 s annealing at 57 °C, and 1 min extension at 72 °C. The protein phosphatase 2A (PP2A) gene was used as a control as it was reported to display maximum stability under abiotic stress conditions [75]. Statistical analysis of the relative quantification data was performed from six biological replicates and three technical replicates using GraphPad Prism (GraphPad Software 8.0.1) software [76].

**Table 2:** List of primers designed for validation of selected candidate genes

Gene name	CoGe Locus ID	Forward primer (5’-3’)	Reverse primer (5’-3’)
*WOX9*	Et_3B_027571	TAAGTACGCGCGCCATTACT	TTGCTGATCCACCATGTCCC
*bZIP23*	Et_1B_011890	CCCCCAAGGCAATGTGTTTG	CCATCTTGCCAAACCCGTTG
*CPK21*	Et_8B_059052	CTTCTCGTCGCCTTCGTCTT	CCAGGTACTCGTCGTTGGTC
*NAC2*	Et_4A_032338	CATGACCACCTCCTACTCGC	GGATGTCGTCGTAGCTGAGG
*DREB1C*	Et_2B_022870	GATGATGATGGAGGAGGCCG	CGCCGTCCATATGCCAATTG
*DREB2A*	Et_3B_030727	CAGTACAGCTGCACCTTCCA	TCCTCGTGATCTCCGTCCTT
*PIP1-3*	Et_1A_007005	GAGGGGAAGGAGGAGGATGT	TACAGGAACAGGAACGTCGC
*PIP2-2*	Et_1B_014398	TTCACCGCCAAGGACTACAC	TGGTGCTTGTACCCGATGAC
*bZIP1*	Et_3B_031108	GGAGTCCCTCCTCGAGATGA	TAGTAGCAGTTGAACGCGGT
*DREB1A*	Et_2A_016724	TCCTTTCCCCGCTATCTCCA	GATGGACATGGCGGATTTGC
*PIP1-1*	Et_1B_013212	TGATCTTCGCGCTCGTCTAC	ACTCCAGCTCCACAGATTGC
*SAP8*	Et_2B_019047	CGTGCAACCCACTGATGTTG	ATAGCGGTGGAGTGCACAAA
*NRT1*	Et_7B_055308	TTTGGAGGTTTTGTGGGGCT	CGCAATCACAAGCAACCCAA
*MATE*	Et_4B_037383	ACGAAAGCTGGGATCACTGG	CGATGGGAATTTGGGTGGGA
*EtDRM3*	Et_9A_062230	CACACTTGGGTACGTCAGCT	GTAATCCTCCGAGGTCGCAG
*EtV5B/CPP1*	Et_8B_060215	TTGCAACTTCCGCTGAGACT	ACGAAAGCTGGGATCACTGG
*Et-PP2A*		CTGAATGTTGCTGGGTCCTCTGC	CACGGGGAGAGCCAGAAGTGC

## Results

### Phylogenetic analysis and mapping pattern of drought-responsive genes

**Fig. 2:**
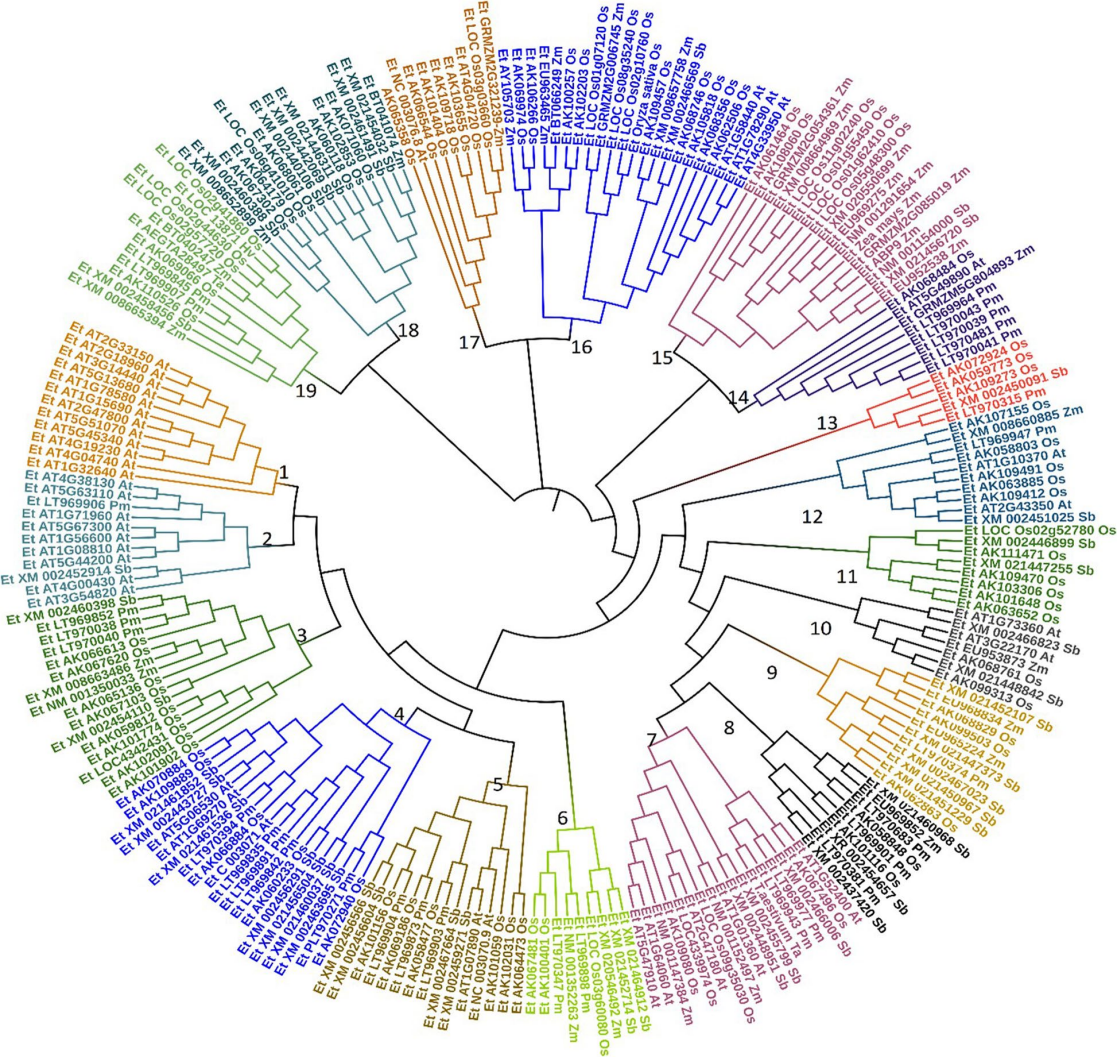
Phylogenetic analysis of drought responsive genes identified in tef. The first two letters in the descriptions represent initials of the *genus* and *species* name of each plant species. Os, *Oryza sativa*; At, *Arabidopsis thaliana;* Zm, *Zea mays;* Sb, *Sorghum bicolor;* Hv, *Hordeum vulgare;* Ta, *Triticum aestivum;* and Pm, *Pennisetum glaucum*

In the phylogenetic tree shown in Fig. 2, a total of 233 tef homologs of drought-responsive genes were identified based on rice, wheat, maize, sorghum, barley, pearl millet and Arabidopsis, and phylogenetic tree was constructed using the Neighbor Joining method. A total of 19 distinct cluster groups were detected (Fig. 2). The clustering pattern was based on the putative functions of the genes across the plant species analyzed. Clustering also shows the presence of diverse drought responsive genes in the tef genome. Many genes were clustered in sub-cluster 16 (22 genes), and 4 (20 genes). Cluster 13 has only five genes followed by cluster 10 with seven genes.

**Fig. 3:**
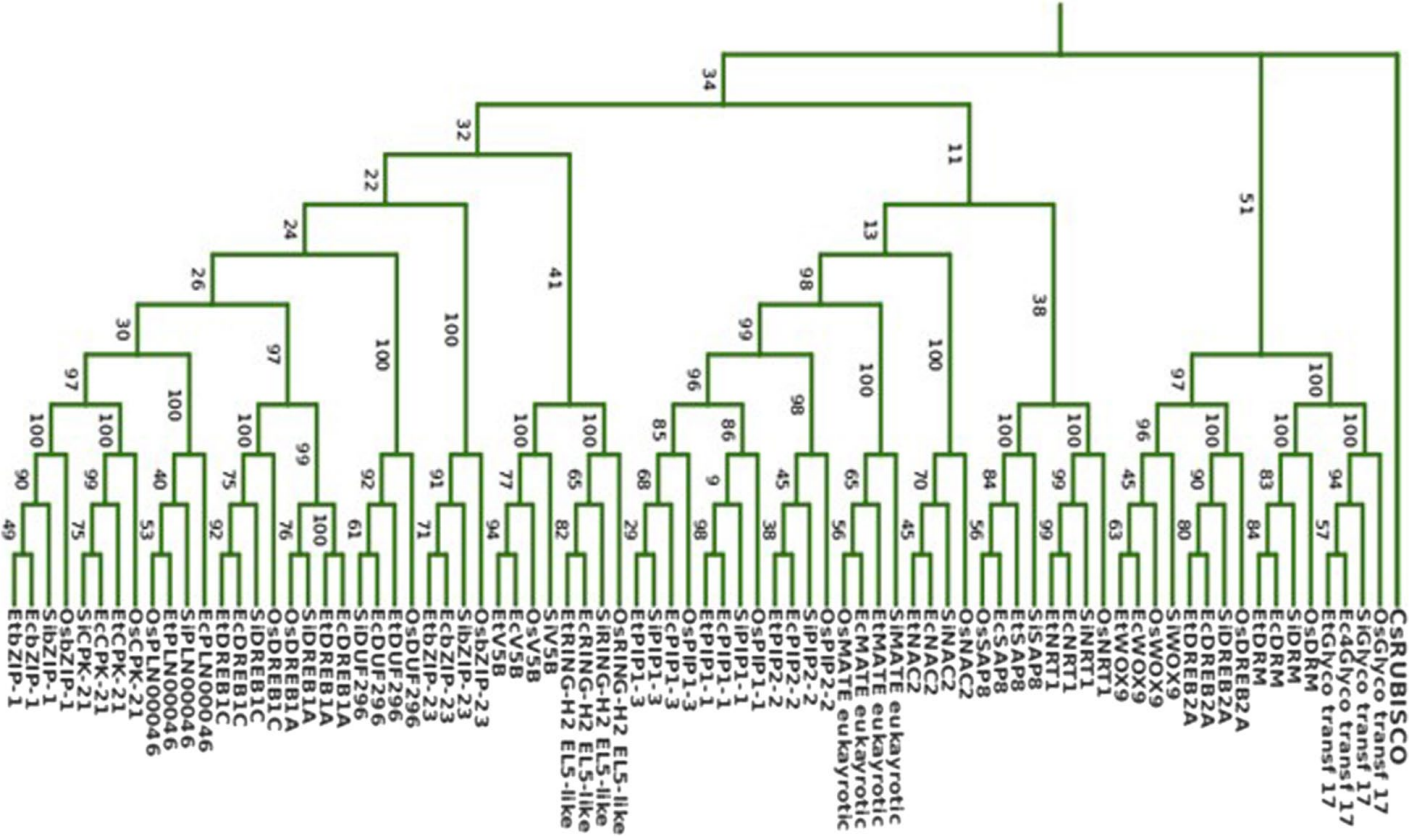
Unscaled NJ tree of 20 CDS of genes with high level GO term compared across four crop plants. Et, *Eragrostis tef*; Os, *Oryza sativa;* Si, *Seteria italica,* and Ec, *Eleusine coracana*

In the second phylogenetic tree (Fig. 3), the CDS version of genes with high level gene ontology terms representing *Eragrostis tef* (Et), *Oryza sativa* (Os), *Setaria italica* (Si) and *Eleusine coracana* (Ec) were used with ribulose bisphosphate carboxylase/oxygenase activase gene (GI: 101206383) as our group sequence (Fig. 3). The top 20 highly enriched genes were clustered into three major groups based on the neighbor-joining tree-building method. Cluster I contains nine gene families including TFs such as *EtbZIP1-1, EtbZIP-23, EtCPK-21, EtDREB1C, EtDREB1A, EtAHL-23,* and *EtCPP1*. Cluster II contains seven gene families *(EtPIP1-1, EtPIP-1–2, EtPIP2-2, EtNRT1, EtMATE, EtNAC2* and *EtSAP8*), and Cluster III contains four gene families including *EtWOX9, EtDREB2A, EtDRM*, and Glyco-transf-17.

The mapping of 253 orthologous drought-responsive genes and their chromosome distribution on the tef genome are presented in Supplementary Fig. 1. Many pearl millet coding genes (505 ESTs) have shown strong identity (> 98%) and homology signal (E = 0.00) with the tef genome. Most of the ESTs from pearl millet were mapped to conting_123 of the tef genome while large arrays of other genes were unevenly mapped to different chromosomes.

### Functional enrichment analysis of drought-responsive genes

As stated in the previous section, a total of 729 drought-responsive genes and ESTs were identified in the tef genome. Of these, 224 genes (Supplementary Table 2) and 29 ESTs that were functionally annotated (Supplementary Table 3) were used for further analysis. From the 224 genes with high homology submitted to DAVID, about 73.4% (160 genes) were functionally annotated with GO term direct at the molecular level (Supplementary Table 4), and about 59.6% (130 genes) were annotated to play a role in known drought-related biological processes (Supplementary Table 5).

In addition to GO term direct, we conducted further annotation and functional enrichment analysis using UP_KW_Biological_Process and UP_KW_Molecular_Function in DAVID to determine genes specifically enriched in both terms. About 87 genes (38.8%) were enriched in different biological processes like abscisic acid signaling pathway, auxin and abscisic acid biosynthesis, auxin signaling pathway, transport, and other biological processes (Supplementary Table 6). Eight of these genes were strongly enriched in stress response (*p* = 4.3E^−05^) in the biological process. Using UP_KW_Molecular_Function, we found 102 genes (45.5%) enriched in a number of molecular functions category including activator, DNA-binding, acyltransferase, transferase, aspartyl esterase, hydrolase, chaperone, chromatin regulator, developmental protein, serine/threonine-protein kinase, dioxygena, methyltransferase, glycosidase, glycosyltransferase, protein phosphatase, ion channel, potassium channel, voltage-gated channel, chloride channel, kinase, isomerase, monooxygenase, and oxidoreductase (Supplementary Table 7). Out of these genes, nine were highly enriched in activation (*p* = 0.006) and DNA binding (*p* = 0.02). The Up-tissue analysis report indicated that the spatial and temporal expression of these genes can be in leaves and roots, and expression can start at the seedling stage (Supplementary Table 8).

Functional annotation of the 505 pearl millet ESTs that showed the highest identity score (> 90%) and low E-Value (< 1E^−50^) when mapped to the tef genome was conducted by Blast2GO software (with E value ≤ 1E^−50^) as they lack official gene ID. Blast2GO conducts GO annotation and functions enrichment analysis by directly comparing sequences to proteins with known functions in available databases using InterPro scan algorithm. From these ESTs, we found 29 (5.7%) (Supplementary Table 3) functionally annotated ESTs distributed in different crops. Most of the ESTs were mapped to *Setaria italica* followed by *Vigna unguiculata.* The GO biological process terms identified using Blast2GO are cellular metabolic process, primary metabolic process, organic substance metabolic process, nitrogen compound metabolic process, biosynthesis, regulation of the cellular process, and methylation (Supplementary Figure 2A). The major GO molecular terms identified are ion binding, heterologous compound binding, organic cyclic compound binding, oxidoreductase activity, and metal cluster binding (Supplementary Figure 2B). Overall, about 25 ESTs were known to be involved in cellular metabolic processes while about eight were found to play a role in metal ion binding at the molecular level.

**Fig. 4:**
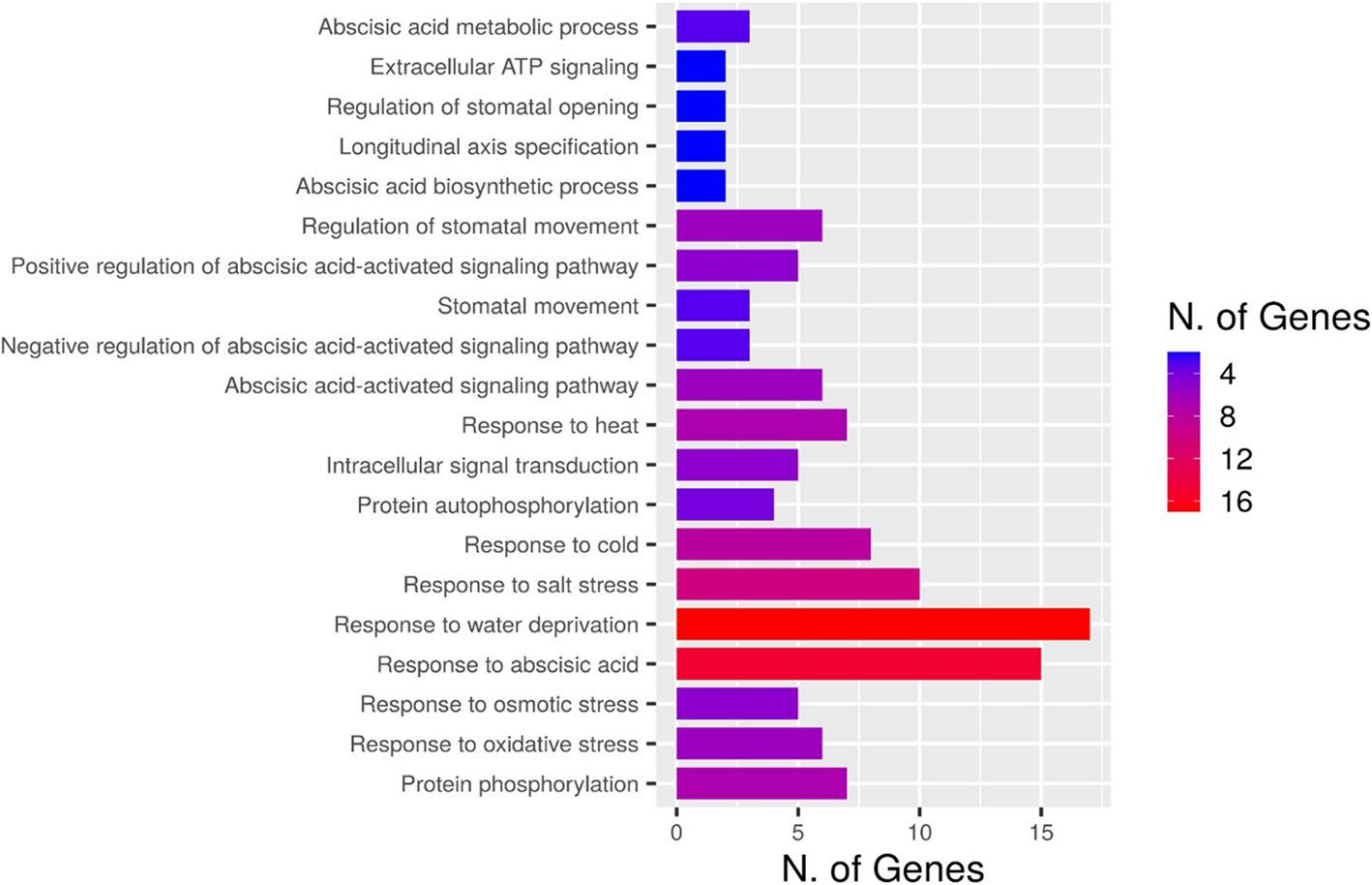
Number of genes that are strongly enriched in different categories of Biological Processes GO term using Arabidopsis as a model (*p* ≤ 1.72E^−05^; FDR ≤ 0.001)

**Fig. 5:**
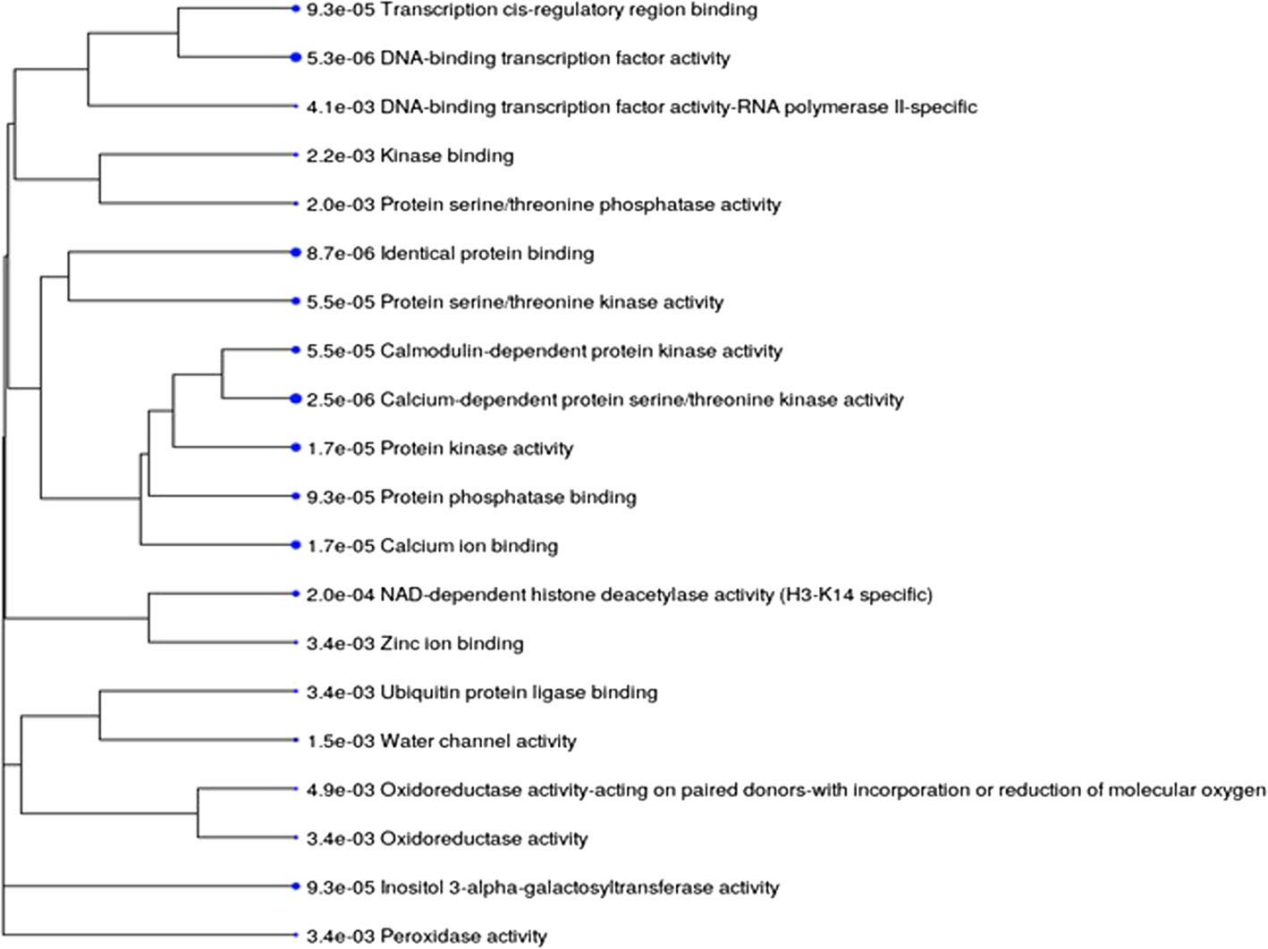
Functional cluster of genes strongly enriched in different categories of Molecular Function GO term at FDR < 0.002. Size of the bubble is proportional with the number of genes in each category. The numbers before the description indicates FDR values

To confirm the accuracy of the GO analysis and the functional annotation obtained from DAVID for multiple crop species, we used single model species *Arabidopsis* and then analyzed the GO term enrichment by ShinyGo software (Supplementary Table 9). The highly enriched genes were shown to have a role in response to abiotic stresses and chemical stimuli (cold, salinity, water deprivation, Osmotic stress, and heat), metabolic process, regulation of the biological process, regulation of the cellular process, primary metabolic process, organic substance metabolic process and response to oxygen-containing compound at the biological process. Figure 4 shows genes that are highly enriched in the biological processes category using Arabidopsis as a model. Some of the top genes with molecular roles identified using ShinyGo software are predicted to have roles in the binding of biomolecules and catalytic activity, abscisic acid 8-hydroxylase activity, calcium-dependent protein serine/threonine kinase activity and calcium ion binding activity (Supplementary Table 10). Figure 5 shows a cluster relationship of genes that are highly enriched in different molecular functions using Arabidopsis as a model and a statistically significant *p*-value (< 0.002). Hence, the outcome from both softwares is comparable even though DAVID appears to be more appropriate for functionally annotating genes from multiple species while ShinyGO is more appropriate for annotating genes from a single species.

### Hierarchical clustering of highly enriched drought-responsive genes

Test-based hierarchical clustering helps to organize genes based on their level of significance and enrichment in known biological processes and molecular function. For hierarchical clustering, we used DAVID software to cluster highly enriched drought-responsive genes. Our analysis showed that the DAVID software gene set enrichment score clusters the genes enriched in different molecular functions into two major cluster groups. Cluster I (Enrichment score = 1.33) contain eight genes (*EtWOX9, EtbZIP-1, EtbZIP23, EtNAC2, EtDREB1C, EtAHL-23, ETDREB1A* and *EtDREB2A*). The second cluster (Enrichment score = 0.78) contains 21 genes having different functional roles at the molecular level (Table 3 and Supplementary Table 11). We further used Kappa coefficient values to classify functionally enriched genes into very high, high, and moderate enrichment (Table 3 and Supplementary Tables 11 and 12). Based on the Kappa value, the genes were clustered with strong functional enrichment into two: Cluster I and II containing 21 and 32 genes, respectively (Supplementary Table 12). Cluster I contain two genes which are dehydration-responsive element-binding protein 1C (LOC4339974, *DREB1C*) and dehydration-responsive element-binding protein 1A-like (LOC4347620, *DREB1A*) that were classified with very high Kappa value (> 0.80). The other genes in cluster I that were classified with high kappa values include dehydration-responsive element-binding protein 2A-like (LOC4324418, *DREB2A*), C-repeat/DRE binding factor 2 (CBF2), NAC domain-containing protein 2-like(LOC4334553), AT-hook motif nuclear-localized protein 23 (LOC4344714, *AHL-23*), light-inducible protein CPRF2 (LOC8061169), C-repeat/DRE binding factor 1(CBF1), *bZIP* transcription factor 23-like (LOC4330838), WUSCHEL-related homeobox 9-like (LOC4324824, *WOX9*), cyclic dof factor 2 (LOC8078579), cyclic dof factor 1 (LOC8082122), *bZIP* transcriptional factor 68-like (LOC110429775), and octopine synthase (ocs) element-binding factor 1 (LOC4326871). About twelve genes with different molecular functions were classified with high Kappa values (> 0.50) (Supplementary Table 12). The second cluster of kappa values contains 21 genes of which five are uncharacterized genes. In this cluster, six genes have very high Kappa values (> 0.76) four of which are uncharacterized (Table 3 and Supplementary Table 12).

**Table 3:** Hierarchical clustering of highly enriched genes using gene set enrichment score and kappa coefficient at molecular function. The kappa coefficient is the de facto standard to evaluate the agreement between raters, which factors out expected agreement due to chance. Kappa value: Very High (0.75–1), High (0.5–0.75), Moderate (0.25–0.5) and Low (< 0.25)

Cluster	Gene ID	Gene Name	Kappa value	Kappa value term
**Cluster 1:** **Enrichment Score: 1.33**	4339974	dehydration-responsive element-binding protein 1C	0.83	Very high
	4347620	dehydration-responsive element-binding protein 1A-like	0.8	Very high
	4324418	dehydration-responsive element-binding protein 2A-like	0.695	High
	4334553	NAC domain-containing protein 2-like	0.64	High
	4344714	AT-hook motif nuclear-localized protein 23)	0.64	High
	4324824	WUSCHEL-related homeobox 9-like	0.59	High
	4330838	bZIP transcription factor 23-like	0.59	High
	4326871	ocs element-binding factor 1	0.5	High
**Cluster 2:** **Enrichment Score: 0.78**	4333169	uncharacterized	0.92	Very high
	4333878	uncharacterized	0.85	Very high
	4332352	uncharacterized LOC4332352	0.83	Very high
	4339571	probable purine permease 4	0.78	Very high
	4336249	protein NRT1/ PTR FAMILY 4.5	0.76	Very high
	4340325	uncharacterized	0.76	Very high
	4350916	uncharacterized LOC4350916	0.72	High
	4345581	CHAPERONE-like protein of por1	0.71	High
	4335799	photosystem I subunit O	0.71	High
	4340300	protein nuclear fusion defective 4	0.7	High
	4333501	protein Detoxification 29	0.62	High
	107278728	rust resistance kinase Lr10	0.62	High
	4329854	beta-1,4-mannosyl-glycoprotein 4-beta-N-acetylglucosaminyltransferase	0.58	High
	4340585	RING-H2 finger protein ATL46	0.57	High
	4337170	probable pectinesterase/pectinesterase inhibitor 13	0.46	Moderate
	4342431	protein Ethylene-Insensitive 2-like	0.46	Moderate
	4342173	potassium transporter 22-like	0.44	Moderate
	4330248	aquaporin PIP1-1-like	0.41	Moderate
	4338289	probable glycerol-3-phosphate acyltransferase 3	0.39	Moderate
	4330049	probable aquaporin PIP2-2)	0.37	Moderate
	4331194	aquaporin PIP 1–3-like	0.35	Moderate

### Pathway analysis of functionally enriched genes

To predict the pathways regulated by the drought-responsive genes identified in this study, we performed the KEGG pathway analysis. Seventy-two of the genes identified in our analysis were predicted to have a role in known cellular pathways including plant hormone signal transduction pathway, carotenoid biosynthesis, MAPK signaling pathway, plant-pathogen interaction, biosynthesis of amino acids and secondary metabolites including sesquiterpenoid and triterpenoid, ABC transporters, biosynthesis, oxidative phosphorylation, carbon fixation and ubiquitin-mediated proteolysis pathway (Supplementary Table 13A & B). Studies have shown that these pathways are implicated in drought stress tolerance [77–79].

### Candidate genes with potentials to mitigate drought-stress

**Fig. 6:**
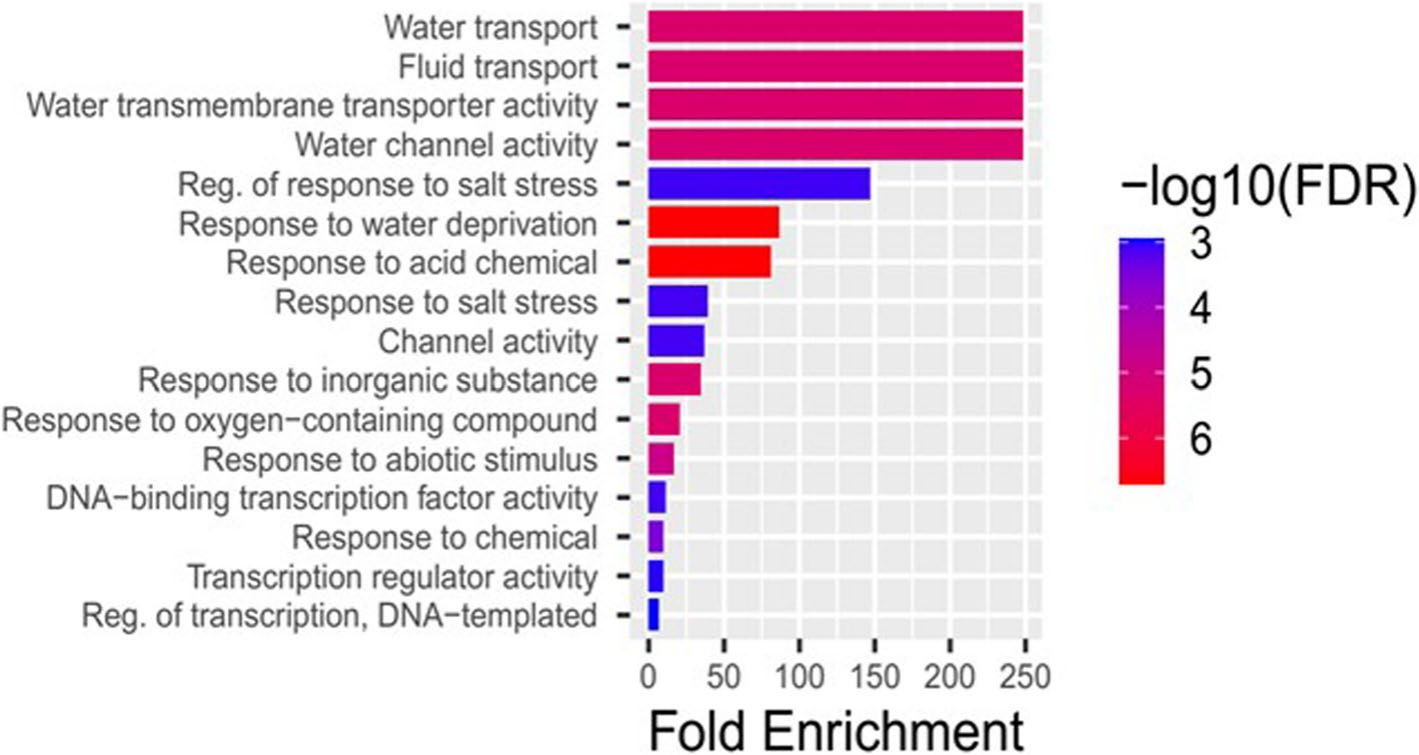
High level GO category of selected drought responsive genes with predicted fold enrichment

Genes strongly enriched in known molecular function and biological processes supported by high-level GO terms have the potential to be utilized in enhancing drought tolerance in plants. Out of the 729 genes of tef initially mapped, we identified a total of 20 candidate genes that were predicted to have a major role in abiotic stress tolerance including drought. The list of these genes with their gene symbol, Entrez ID, and putative functional role is shown in Table 4. Based on the *in-silico* analysis, it can be concluded that these genes are possible candidates for future breeding programs to improve drought tolerance. Table 4 shows 20 genes with high-level GO terms and Fig. 6 depicts a fold enrichment analysis of these genes in the high-level GO category. Most genes were predicted to play a role in a number of biological activities and may not be limited to a single process. For instance, in our present analysis the *bZIP* family genes were predicted to have an association with 13 out of 20 high-level biological categories (Supplementary Table 14).

**Table 4:** Candidate genes for future stress drought mitigation

Entrez_ID	gene symbol	Gene description
**4324824**	*EtWOX9*	WUSCHEL-related homeobox 9; Transcription factor which may be involved in the specification and maintenance of the stem cells (QC cells) in the root apical meristem (RAM); Belongs to the WUS homeobox family
**4326871**	*EtbZIP-1*	bZIP transcription factor, bZIP-1 domain-containing protein. ocs element-binding factor 1
**4330838**	*EtBZIP23*	Transcriptional activator that mediates abscisic acid (ABA) signaling (PubMed: 18,931,143, PubMed: 19,947,981, PubMed: 27,424,498, PubMed: 27,325,665). Can regulate the expression of a wide spectrum of stress-related genes in response to abiotic stresses through an ABA-dependent regulation pathway. Confers ABA-dependent drought and salinity tolerance. Binds specifically to the ABA-responsive elements (ABRE) in the promoter of target genes to mediate stress-responsive ABA signaling. Its principal role is in Plant hormone signal transduction pathway and assist in stomatal closure
**4334553**	*EtNAC2*	Transcription factor that possesses transactivation activity. Transcription activator involved in response to abiotic stresses. Plays a positive role during dehydration and salt stress. Binds specifically to the 5'-CATGTG-3' motif found in promoters of stress-responsive genes
**4337721**	*EtDRM3*	Involved in de novo DNA methylation. Involved in RNA-directed DNA methylation (RdDM)
**4339974**	*EtDREB1C*	Dehydration-responsive element-binding protein 1C; Transcriptional activator that binds specifically to the DNA sequence 5'-[AG]CCGAC-3'. Binding to the C-repeat/DRE element mediates high salinity- and dehydration-inducible transcription (By similarity)
**4344714**	EtAHL-23	AT-hook motif nuclear-localized protein; Transcription factor that specifically binds AT-rich DNA sequences related to the nuclear matrix attachment regions (MARs)
**4347620**	*EtDREB1A*	Dehydration-responsive element-binding protein 1A; Transcriptional activator that binds specifically to the DNA sequence 5'-[AG]CCGAC-3'. Binding to the C-repeat/DRE element mediates high salinity- and dehydration-inducible transcription. Confers resistance to high salt, cold and drought stress
**4324418**	*EtDREB2A*	dehydration-responsive element-binding protein 2A-like. Transcriptional activator that binds specifically to the DNA sequence 5'-[AG]CCGAC-3'. Binding to the C-repeat/DRE element mediates high salinity- and dehydration-inducible transcription
**4331194**	*EtPIP1-3*	Aquaporin PIP 1–3; Water channel required to facilitate the transport of water across cell membrane. Increases the capacity for root water uptake under water deficit. May play a role in drought avoidance in upland rice
**4341520**	*EtSAP8*	Stress associated protein 8. Zinc finger A20 and AN1 domain-containing stress-associated protein 8; Involved in environmental stress response
**4346187**	*EtCPK21*	May play a role in signal transduction pathways that involve calcium as a second messenger (By similarity). Functions in signal transduction pathways that positively regulate responses to abscisic acid (ABA) and salt stress. It also plays a role in Plant-pathogen interaction pathway
**4330049**	*EtPIP2-2*	aquaporin PIP2-2; Aquaporins facilitate the transport of water and small neutral solutes across cell membranes
**4330248**	*EtPIP1-1*	Aquaporin PIP1-1; function as water channel to facilitate the transport of water across cell membrane; Belongs to the MIP/aquaporin (TC 1.A.8) family
**4336249**	*EtNRT1*	protein NRT1/ PTR FAMILY 4.5
**4345581**	*EtCPP1*	Chaperone-like protein of protochlorophyllide oxidoreductase (POR), J-like protein, Regulation of chlorophyll biosynthesis
**4335799**	*EtPLN00046*	photosystem I subunit O. Plays a role in Photosynthesis and Metabolic pathways,
**4333501**	*EtMATE*	protein Detoxification 29. Multi antimicrobial extrusion protein MatE family protein
**4329854**	*GNT3*	beta-1,4-mannosyl-glycoprotein 4-beta-N-acetylglucosaminyltransferase. Has principal role in N-Glycan biosynthesis and Metabolic pathways
**4340585**	*EtRING*	RING (really interesting new gene); RING-H2 finger protein ATL46

### Network analysis of selected candidate genes with high level GO terms

**Fig. 7:**
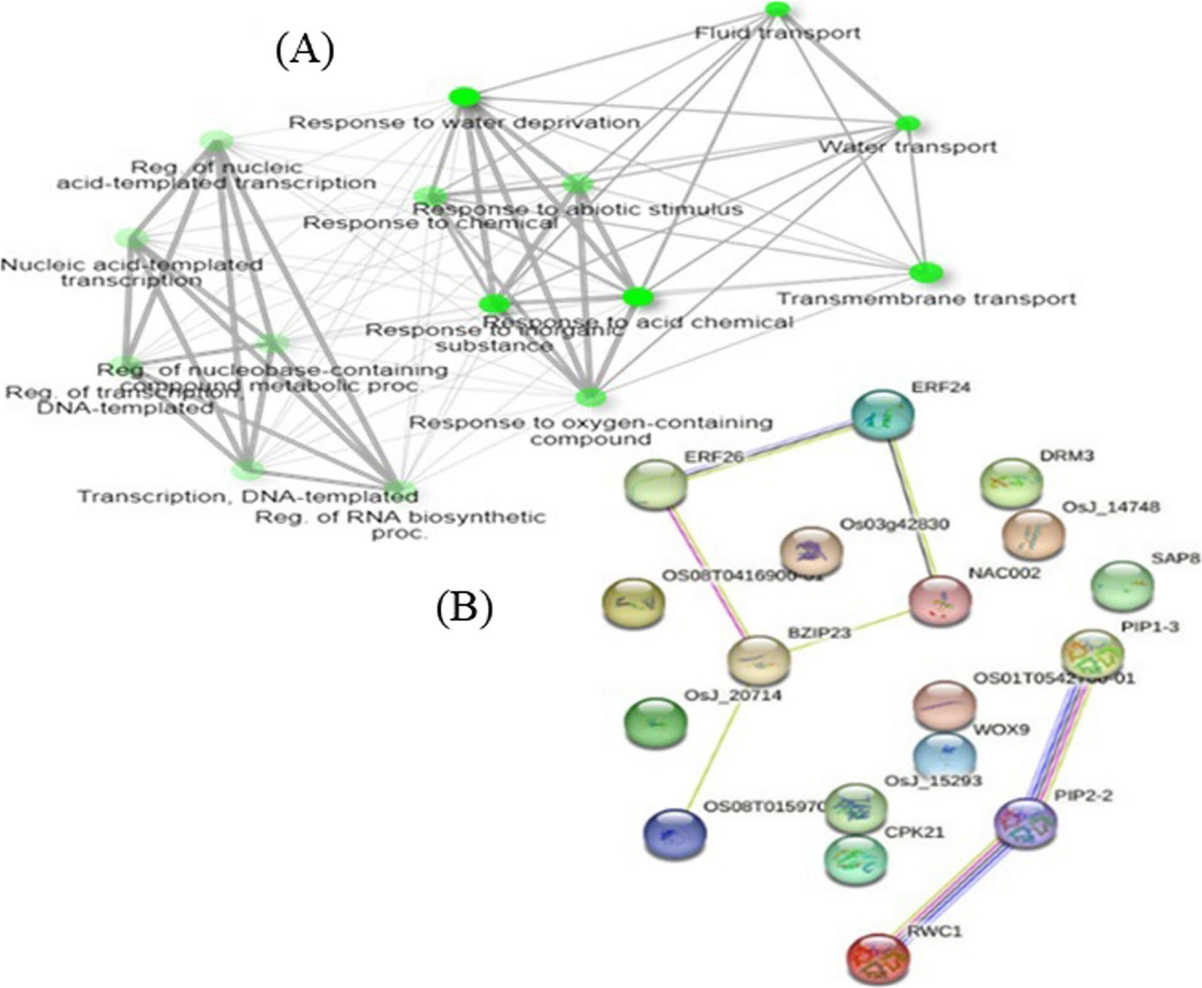
Gene–gene and protein–protein interaction network of selected candidate genes. **A** Each node represents an enriched GO term. Related GO terms are connected by a line, whose thickness reflects percent of overlapping genes. The size of the node corresponds to number of genes. Darker nodes represent more significantly enriched gene sets. Bigger nodes represent larger gene sets. Thicker edges represent more overlapped genes. **B** Represent protein–protein interaction. Line thickness represent level of interaction (thicker line for strong interactions)

To predict gene–gene and protein–protein interaction network of the selected 20 candidate genes, network analysis was performed at gene and protein levels. A gene interaction network is a set of genes (nodes) connected by edges representing functional relationships among these genes. Genes are thought to have either a physical interaction through their translation products (proteins), or one of the genes alters or affects the activity of another gene of interest [80]. The functional products of genes work together to accomplish a particular function, and they often physically interact with each other to carry out a more complex biological process. Figure 7A shows gene to gene interaction network of selected candidate genes. Accordingly, genes may interact directly or indirectly with one another. For instance, genes predicted to be involved in water transport were found to directly interact with eight other genes, including those involved in fluid transport, water deprivation, abiotic stresses, chemical stimuli, oxygen-containing compounds, inorganic substrates, stresses, and genes responsive to acid chemicals (Fig. 7A).

Similarly, protein–protein interaction (PPI) network of selected genes was also conducted (Fig. 7B). Proteins usually interact with one another or with other molecules like DNA or RNA to mediate metabolic and signaling pathways, cellular processes, and organismal systems [81]. For example, the *PIP1-1, PIP2-2,* and *bZIP-1* proteins are predicted to strongly interact with one another, but not with the *PIP1-3* protein. The *PIP1-3* protein was predicted to physically interact with the *SAP8* protein. *DREB1A* and *DREB1C* are also predicted to directly interact with *NAC2* and *bZIP23* proteins.

### Analysis of conserved domains in selected candidate genes

To determine the nature and putative roles of conserved domains present in the selected 20 genes in tef, we compared their domains with genes from widely studied japonica rice. Of the 20 genes, 12 have comparable conserved and binding sites in both rice and tef, suggesting functional similarity between a C_3_ and a C_4_ plant. However, we found distinct differences for the remaining eight genes. Dehydration-responsive element binding protein 1C (*DREB1C*) of tef has a shorter APETALA2 (AP2) binding site (31 amino acids) compared to rice (60 amino acid) (data not shown). Likewise, the tef TF *bZIP-1* (32 amino acids) has a shorter DNA binding domain compared to rice (38 amino acids). However, the nature and roles of conserved amino acids in *bZIP-1* are similar in both tef and rice.

**Fig. 8:**
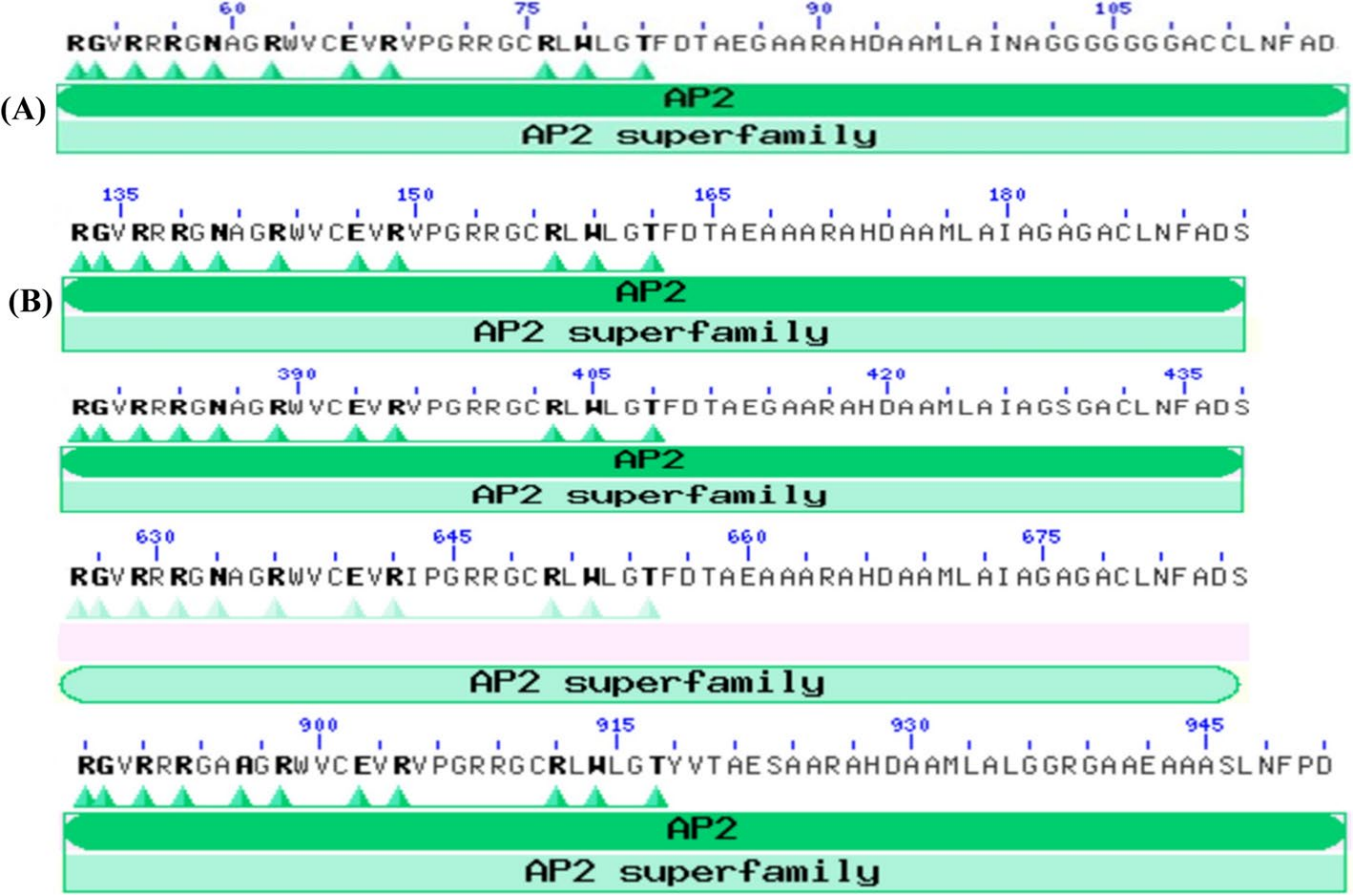
Conserved domain analysis of selected genes. **A** Rice DREB1A with single AP2 binding site, and **B** Tef DREB1A with four structurally related copes within single transcriptional machinery. The amino acids in bold are predicted significant binding sites

On the other hand, tef has four AP2 conserved domains with 60–68 amino acids on the same TF *DREB1A* while *DREB1A* of rice has only one AP2 conserved domain (Fig. 8). In tef, *CPK21* gene binding domain (STKc_CAMK) is twofold longer (279 amino acids) than the rice homolog (138 amino acids). In addition to STKc_CAMK domain, *CPK21* gene of tef has another Ca^2+^ binding domain (FRQ1 domain with 152 amino acids). The *PIP1-1* and *PIP2-2* tef homologs have three-fold longer conserved domain as compared to the rice homolog. Another interesting difference was the presence of three copies of chemical substrate Major Facilitator Superfamily (MSF) with single transcriptional machinery in the tef *NRT1* gene while only one MFS domain was detected for the rice *NRT1*. The conserved domain of GNT3, a beta-1,4-mannosyl-glycoprotein 4-beta-N-acetylglucosaminyltransferase of tef is also longer (348 amino acids) than that of rice (222 amino acids). The description of the candidate genes and their conserved domains is illustrated in Table 5.

**Table 5:** Conserved domain report of genes with high level gene ontology term recommended for future breeding

**Gene:ID**	**Gene name**	**Domain name**	**CD description**	**Domain Interval**	**E-value**	**CD length (amino acid)**	**CDS length (amino acid)**
**4324824**	*EtWOX9*	homeodomain super family	DNA binding domains involved in the transcriptional regulation of key developmental processes	40–213	8.3E-11	58	201
**4326871**	*EtbZIP-1*	bZIP plant GBF1	Basic leucine zipper (bZIP) domain of Plant G-box binding factor 1 (GBF1)-like transcription factors. DNA-binding and dimerization domain. GBFs are involved in developmental and physiological processes	124–237	8.5E-11	38	158
**4330838**	*EtbZIP-23*	bZIP plant BZIP46	Similar description with bZIP-1	745–879	2.2E-20	45	344
**4334553**	*EtNAC2*	NAM	No apical meristem (NAM) protein involved in plant development proteins. Mutations in NAM result in the failure to develop a shoot apical meristem in petunia. NAM plays a role in determining positions of meristems and primordial	49–420	1.0E-61	124	294
**4337721**	*EtDRM3*	Dcm super family	site specific DNA- cytosine methylase (replication, recombination and repair)	1429–1785	1.5E-03	119	597
**4339974**	*EtDREB1C*	AP2 super family	DNA-binding domain found in transcription regulators in plants such as APETALA2 and EREBP (ethylene responsive element binding protein). EREBP involved in stress response, contain a single copy of the AP2 domain. APETALA2-like proteins, which play a role in plant development contain two copies	121–213	3.1E-10	31	218
**4347620**	*DREB1A*	AP2	Description similar with DREB1C	889–1071	1.4E-20	61	1052
		AP2		2416–2613	1.2E-13	66	
		AP2		151–333	3.5E-10	61	
		AP2 super family		1630–1812	1.7E-09	61	
**4324418**	*EtDREB2A*	AP2	DNA-binding domain in plant proteins such as APETALA2 and EREBPs	169–354	2.3E-31	61	372
**4331194**	*EtPIP1-3*	MIP	Major intrinsic protein (MIP) family that exhibit essentially two distinct types of channel properties: (1) specific water transport and (2) small neutral solutes transport	136–825	2.0E-99	230	288
**4330049**	*EtPIP2-2*	MIP	Similar with EtPIP1-3	97–810	1.4E-92	238	288
**4330248**	*EtPIP1-1*	MIP	Similar with EtPIP1-3	136–825	1.7E-99	230	289
**4336249**	*EtNRT1*	MFS super family	Major Facilitator Superfamily (MFS) is a large and diverse group of secondary transporters that includes uniporters, symporters, and antiporters. MFS proteins facilitate the transport across cytoplasmic or internal membranes of a variety of substrates including ions, sugar phosphates, drugs, neurotransmitters, nucleosides, amino acids, and peptides	1714–3261	3.0E-161	516	1613
		MFS super family		70–1569	2.0E-110	500	
		MFS super family		3283–4719	2.0E-108	479	
**4345581**	*EtCPP1/V5B*	CPP1-like	CHAPERONE-LIKE PROTEIN OF POR1 (CPP1), is an essential protein for chloroplast development, plays a role in the regulation of POR (light-dependent protochlorophyllide oxidoreductase) stability and function	247–672	2.6E-46	142	1613
**4335799**	*PLN00046*	PLN00046	photosystem I reaction center subunit O	4–429	1.1E-72	142	242
**4333501**	*MATE*	MATE_eukaryotic	Eukaryotic members of the multidrug and toxic compound extrusion (MATE) family. MATE has been identified as a large multigene family linked to disease resistance. Acts as solute transporters responsible for secretion of cationic drugs. Has also a role in iron homeostatis under osmotic stress	31–1332	2.0E-150	651	144
		A1904 super family	K + -dependent Na + /Ca + exchanger; [Transport and binding proteins, Cations and iron carrying compounds]	52–702	2.5E-04	217	
**4329854**	*GNT3*	Glyco_transf_17	Glycosyltransferase family 17. This family represents beta-1,4-mannosyl-glycoprotein beta-1,4-N-acetylglucosaminyltransferase. This enzyme transfers the bisecting GlcNAc to the core mannose of complex N-glycans	211–1254	0.0	348	388
**4340585**	*EtRING*	RING-H2_EL5-like	RING finger, H2 subclass, found in rice E3 ubiquitin-protein ligase EL5 and similar proteins. EL5 acts as an anti-cell death enzyme	478–609	7.8E-22		407
**4341520**	*EtSAP8*	ZnF_AN1	AN1-like Zinc finger; Zinc finger at the C-terminus of An1, a ubiquitin-like protein in Xenopus laevis	334–426	1.9E-08	31	141
		zf-A20	A20-like zinc finger; The A20 Zn-finger is a Ubiquitin Binding Domain	43–114	8.4E-08	24	
**4344714**	*AHL-23*	DUF296	This domain is found in proteins that contain AT-hook motifs, which suggests a role in DNA-binding for the proteins as a whole	283–528	2.1E-21	82	287
**4346187**	*EtCPK21_like*	STKc_CAMK	The catalytic domain of CAMK family Serine/Threonine Kinases. STKs catalyze the transfer of the gamma-phosphoryl group from ATP to serine/threonine residues on protein substrates. CaMKs are multifunctional calcium and calmodulin (CaM) stimulated STKs involved in cell cycle regulation	244–1077	1.9E-125	278	618
		FRQ1 super family	Ca2 + -binding protein, EF-hand superfamily	1291–1776	4.0E-22	162	

### Quantitative RT-PCR validation of selected drought-responsive genes

**Fig. 9:**
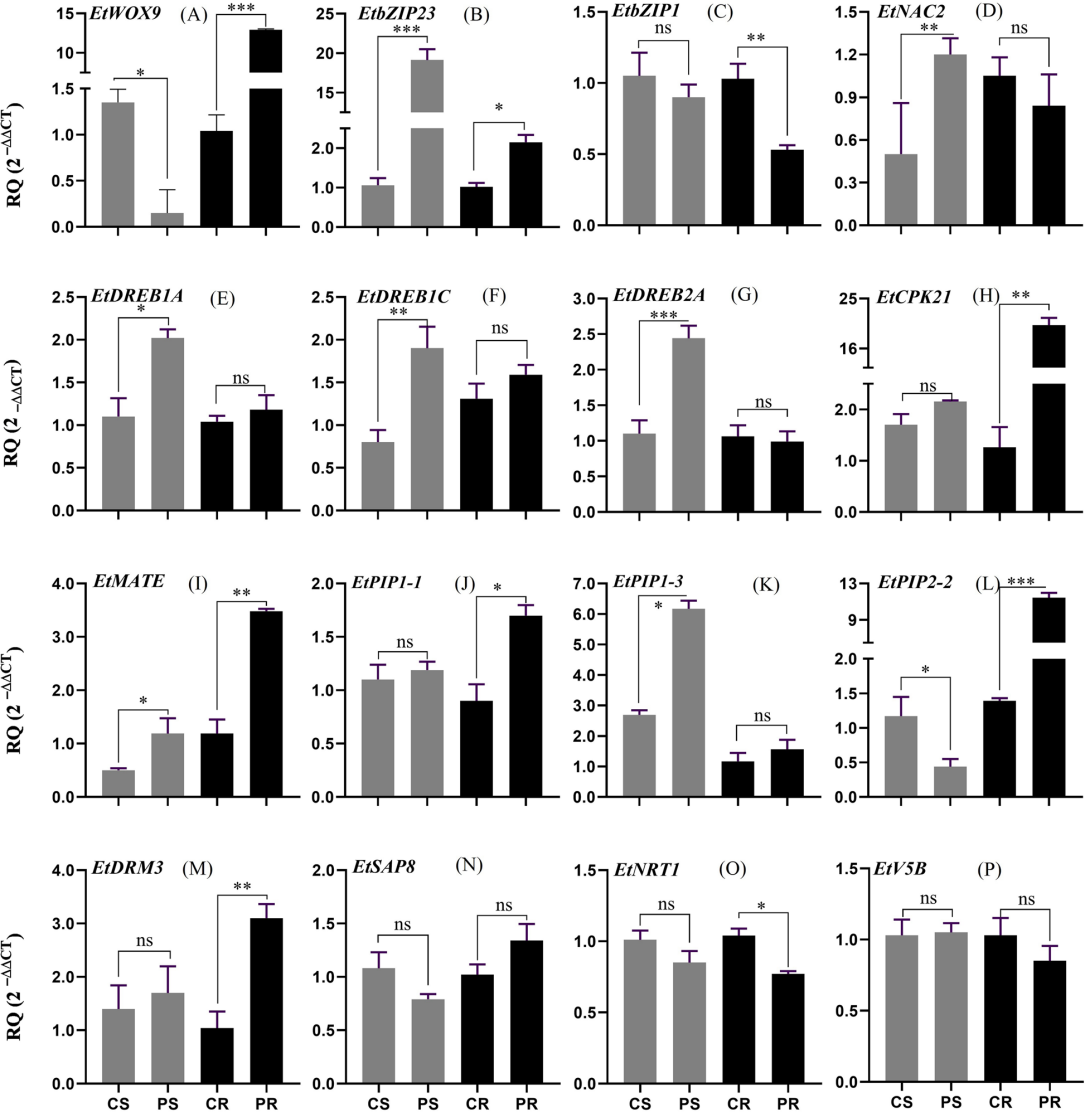
Analysis of drought-responsive genes expression using qRT-PCR in shoot (grey bar) and root (black bar) tissues of tef (Dabbie).CS, control shoot; PS, PEG treated shoot, CR, control root, and PR, PEG treated root. **A** *EtWOX*9, **B ***EtbZIP2*3, **C ***EtbZIP1*, **D ***EtNAC2*, **E ***EtDREB1*, **F ***EtDREB1C*, **G ***EtDREB2A;***H ***EtCPK21*, **I ***EtMATE*, **J ***EtPIP1-1*, **K ***EtPIP1-2*, **L ***EtPIP1-3*, **M ***EtDRM3*, **N ***EtSAP8*, **O ***EtNRT1*, and **P ***EtV5B*. ns, *p* > 0.05; *, *p* ≤ 0.05; **, *p* ≤ 0.01; ***, *p* ≤ 0.001)

Quantitative RT-PCR was performed to validate the expression of selected candidate drought-responsive genes in tef. We analyzed the expression of 16 genes in both shoot and root in response to osmotic stress induced by PEG8000. As shown in Fig. 9, the expression of 14 candidate genes was deferentially regulated in shoots, roots or both tissues by PEG-treatment. In shoots, the expression of *EtbZIP23*, *EtNAC2*, *EtDREB1A*, *EtDREB1C*, *EtDREB2A*, *EtMATE* and *EtPIP1-3* (Fig. 9B, D, E, F, G, I, and K, respectively) was upregulated while in roots, the expression of *EtWOX9*, *EtbZIP23*, *EtCIPK21*, *EtMATE*, *EtPIP1-1*, *EtPIP2-2* and *EtDRM3* (Fig. 9A, B, H, I, J, L and M, respectively) were upregulated by PEG treatment. Whereas *EtZIP1* (Fig. 9C) and *EtNRT1* (Fig. 9O) were significantly downregulated in roots while *EtWOX9* and *EtPIP2-2* were significantly downregulated in shoots by PEG induced osmotic stress. In shoots, the expression of *EtbZIP1, EtCIPK21, EtPIP1-1, EtDRM3, EtSAP8, EtNRT1* and *EtV5B* (Fig. 9C, H, J, M, N, O, and P, respectively) was not significantly affected by the PEG treatment while the expression of *EtSAP8* (Fig. 9N) and *EtV5B* (Fig. 9P) *was* not affected by the PEG treatment in both tissues. The fold increase in gene expression in response to PEG-induced osmotic stress was the highest for *EtCIPK21* (Fig. 9H) followed by *EtWOX9* (Fig. 9A) and *EtPIP2-2* (Fig. 9L) (20-, 14- and eightfold, respectively) in roots and *EtbZIP2*3 ((Fig. 9B) (20-fold) in shoots. Overall, the expression of the selected candidate genes showed varying degrees of response to PEG-induced osmotic stress in shoots and roots.

## Discussion

Tef is a C_4_ crop relatively tolerant to drought due to physiological and genetic mechanisms compared to C_3_ crops [82]. Therefore, identifying genes that regulate drought tolerance in tef is paramount important to improve drought-sensitive cultivars or crops and enhance crop yields under drought-prone conditions. In this study, we performed *in silico* analysis to retrieve 729 drought-responsive genes representing Arabidopsis, maize, sorghum, barley, wheat, and pearl and identified highly enriched candidate genes in tef. We used CoGEBlast to identify the collected drought-responsive genes in the tef genome and MEGA-11 to study the relationship of drought-responsive tef genes. Moreover, we used DAVID, ShinyGO, and BLAST2GO for gene orthology and enrichment analysis. To further confirm the pattern of gene expression in highly enriched genes, osmotic stress was induced by PEG and qRT-PCR was used using root and shoot samples and reported in terms of relative gene expression.

Using gene enrichment analysis, we categorized all the genes based on biological and molecular functions by utilizing statistical indices including *p*-value, FDR, and Kappa coefficient, and selected 20 genes that are predicted to have significant roles in drought tolerance and conducted genes and proteins interaction network analysis.

One of these genes is the WUSCHEL-Related Homeobox (*WOX9*)-like gene which we putatively named *EtWOX9* for *Eragrostis tef WOX9.* The qRT-PCR relative gene expression analysis indicated that *WOX9* was 13-fold upregulated in PEG-treated roots while it was downregulated in PEG-treated shoots (Fig. 9). The *WOX9* gene may have a role in plants including the regulation of developmental processes. The *WOX* family is the homeobox transcription factor superfamily playing many functions in embryonic growth to organ formation in plants [83]. This gene family is implicated in developmental processes including cell division, development, stem cell repair, organ formation, seed formation, tissues, and organ regeneration [83–86]. Recent studies have indicated that *WOX* genes play a role in the regulation of abiotic stress resistance including drought [87]. In *Glycine max,* expression of the *WOX* gene family has been shown to be induced by drought, heat, cold, and salt stress in [84], and in Arabidopsis, *WOX9* was reported to be expressed in the root tip meristem tissue and promote root cell multiplication which is a mechanism of drought tolerance [88, 89].

The other gene family that showed high-level GO classification was the basic leucine zipper (*bZIP*) transcriptional family genes. Two genes putatively named *EtbZIP-1* and *EtbZIP23* were identified in this study. The *bZIP* family proteins are the largest TFs family and the most diverse family that are implicated in various abiotic stress responses [90–92]. Our finding suggested that the two *bZIP* proteins play a role in the regulation of biological processes, cellular processes, stimulus and stress responses, biosynthetic processes, regulation of metabolic processes, and some signaling pathways (Table 4). Our analysis revealed that the *bZIP23* may also play a role in mediating abscisic acid (ABA) signaling, regulating the expression of a wide range of stress-related genes in response to abiotic stresses through an ABA-dependent regulation pathway, conferring ABA-dependent drought and salinity tolerance. *bZIP23* is known to bind specifically to the ABA-responsive elements (ABRE) of the promoter of target genes to mediate stress-responsive ABA signaling, and phytohormone signal transduction pathway and stomatal closure [92]. The qRT-PCR relative gene expression analysis indicated that *bZIP23* was upregulated in both PEG-treated roots and shoots within 30 h of drought induction (Fig. 9). However, *bZIP-1* was not found to be co-overexpressed with *bZIP23* in both roots and shoots. A number of studies indicated that *bZIP* family proteins are strongly associated with drought tolerance in crops [93–95]. Though bZIP-1 and *bZIP23* are highly enriched in the current analysis, there are about 93 *bZIP* family transcriptional factors (TF) so far reported in tef [96]. These TFs have not been isolated and characterized in tef. In Arabidopsis, the *bZIP23* and *bZIP19* were reported to be involved in Zn uptake and accumulation and were regarded as Zn sensors [97, 98]. Since tef is rich in Zn and Fe, *bZIP* TFs may play a role in Zn accumulation in tef; however, this needs further studies [31].

We also identified the *EtNAC2* gene which is a member of the *NAC* TFs family which are involved in biotic and abiotic stress responses. Tef was reported to have 172 putative *NAC* transcriptional factors [96] from which *NAC2* was found with high-level GO term. Expression analysis of the *NAC2* showed that it is upregulated in PEG-treated shoots whilst it was downregulated in roots (Fig. 9). The *NAC2* gene was predicted to play a role during dehydration and salt stress by binding specifically to the 5'-CATGTG-3' motif found in promoters of stress-responsive genes (Table 4). The transcriptional factor belonging to NAC (NAM, ATAF, and CUC) family has been widely recognized as plant biotic and abiotic stress-responsive factors [99, 100]. Some NAC TFs were implicated in the regulation of senescence and drought response [101, 102]. In Arabidopsis, overexpression of three *NAC* genes (ANAC019, ANAC055, and ANAC072) which were induced by drought stress improved stress tolerance in the transgenic lines compared to the wild type [103].

In this study, the expression of *DRM3* gene was upregulated in both root and shoots though the level of expression is higher in shoots in response to drought stress (Fig. 9). The epigenetic role of the *DRM3* gene in response to drought was previously reported from studies using whole genome bisulfite sequencing of mulberry (*Morus alba*) [94]. In Arabidopsis, *DRM3* lacks catalytic activity and is reported to play a role in promoting DNA Pol V transcriptional elongation factor, controlling DNA methylation, and regulating RNA polymerase V transcript abundance [104]. Similarly, our analysis suggests that the mechanism by which this gene promotes drought tolerance could be through de novo DNA methylation and RNA-directed DNA methylation (RdDM) activities, suggesting that drought tolerance mechanism in tef could involve epigenetic mechanism.

Dehydration-responsive element-binding (*DREB*) proteins are members of TFs that are well-studied for their role in abiotic stress tolerance including drought, salt, and cold and contain conserved APETALA2/ethylene responsive factor (AP2/ERF) DNA binding domain [105, 106]. In this analysis, we identified three *DREB* TFs including *EtDREB1A, EtDREB2A,* and *EtDREB1C* with high-level GO terms. All three DREB families have shown different degrees of expression in shoots, though the *DREB2A* transcript was relatively higher. However, there was no change in the relative expression of *DREB2A* in roots while *DREB1C* and *DREB1A* transcripts are upregulated in PEG-treated shoots (Fig. 9). Overexpression of the Arabidopsis *DREB1A* gene under the regulation of stress-inducible rd29A promoter has been reported to improve drought and low-temperate tolerance in transgenic tobacco [107]. Similarly, co-overexpression of *DREB2A* and ascorbate peroxidase (APX) in Indica Rice (*Oryza sativa* L.) resulted in drought tolerance enhancement [108]. Furthermore, overexpression of full-length and partial *DREB2A* was reported to enhance soybean drought tolerance [109]. Recently, *DREB1C* has been shown to regulate nitrogen-use efficiency and flowering time in rice and help to boost grain yields and shorten the growth duration of rice [110]. In addition to shortening flowering time, *DREB1C* regulates the expression of several important growth-related genes including nitrate transporters and nitrate reductase, display a higher harvest index and increased remobilization of N and C to sink organs [110]. The biological role of tef *DREB* proteins remains to be characterized.

The Plasma Membrane Aquaporin (AQP) *PIPI1-1, PIP1-3,* and *PIP2-2* are another group of genes detected by high-level GO term in this analysis. The *PIP1-1* and *PIP1-3* were up-regulation in both shoot and roots though *PIP1-3* transcript was relatively higher in shoots. *PIP2-2* was 13-fold selectively up-regulated in roots whilst it was down-regulated in shoots (Fig. 9). As members of major intrinsic proteins, AQPs facilitate the transport of water, glycerol, and small uncharged solutes through the cell membranes [111]. Our *in-silico* analysis predicted that these aquaporins function as water channels to facilitate the transport of water and small neutral solutes across the cell membrane. *PIP2* aquaporins are implicated in water transport when expressed in *Xenopus* oocytes and yeast whereas most *PIP1*s do not have significant water channel activity, however, PIP1 type aquaporins have been shown to interact with PIP2 type channels to facilitate water transport [112–115]. Previously, Ren et al. [116] conducted a meta-analysis on the effect of the overexpression of the aquaporin gene family on drought stress response and reported that the *PIP2* gene family has positive effects on drought tolerance in transgenic plants.

Our analysis also detected the *EtCPP1* gene with highly enriched GO terms. It is predicted to play a role in the regulation of chlorophyll biosynthesis. The rice chaperone-like protein of protochlorophyllide oxidoreductase (POR), J-like protein has been reported to play a role in chlorophyll and tocopherol biosynthesis [117]. J-like proteins have been shown to modulate the functions of Hsp70, J-domain protein (JDP) systems in novel ways thereby regulating diverse plant processes [118].

The *NRT1* protein was another drought-responsive gene that was detected in our analysis. However, its transcripts were downregulated in both PEG-treated shoots and roots (Fig. 9). The *NRT1/NPR* has been reported to facilitate carbohydrate and nitrogen accumulation in drought-stressed genotypes of grapevine [119]. Some NPF transporters can also transport different substrates, such as nitrate/auxin, nitrate/abscisic acid, nitrate/glucosinolate, or gibberellin/jasmonic acid [119–121]. In rice, *OsNPF3.1* is a member of the *NRT1/PTR* genes that has been reported to affect plant height by increasing the nitrogen use efficiency [117]. Thus, downregulation of its expression might lead to lodging tolerance while decreasing nitrogen uptake.

Calcium-dependent protein kinases (*CDPK*s or *CPK*s) are key calcium-binding proteins that have pivotal role in abiotic stress tolerance through activation and regulation of several genes, transcription factors, enzymes, and ion channels by ABA-dependent manner [122]. Overexpression of *OsCPK21* increases ABA levels and enhances salt tolerance by regulating and inducing the salt tolerance genes [123]. In our analysis, *CPK21* was one of the genes identified in tef with high level GO term. The *CPK21* functions in signal transduction pathways that positively regulate responses to abscisic acid (ABA) and salt stress. The qRT-PCR relative gene expression analysis revealed that *CPK21* was up-regulated in both shoots and roots (Fig. 9). The relative up-regulation was higher in PEG-treated roots (24-fold) when compared with shoots.

Furthermore, *EtMATE*, *GNT3, EtRING* were detected with high GO terms in the tef genome. Our *in-silico* analysis showed that *EtMATE* plays a role in the protein detoxification process and multi-antimicrobial extrusion process. MATE (Multidrug and Toxic Compound Extrusion or Multi-Antimicrobial Extrusion) transporters comprise a universal gene family of membrane transporters that are present in all kingdoms of life. The *EtMATE* transcripts were upregulated in both roots and shoots though the level of expression was higher in roots (Fig. 9). *MATE* transporters have been implicated directly or indirectly in mechanisms of detoxification of noxious compounds or heavy metals, tolerance to aluminum toxicity, disease resistance, nutrient homeostasis, such as Fe^3+^ uptake, and the transport of diverse types of secondary metabolites, such as alkaloids, flavonoids, and anthocyanidins, as well as hormones, such as ABA, salicylic acid, and auxin [124].

To further understand the nature of functionally conserved domains among selected genes in C_4_ and C_3_, conserved domain identification was performed based on the full-length CDS (Fig. 8). The protein coded by *DREB* family genes contains a conserved AP2 domain, which consists of approximately 60 amino acids [125]. The AP2 subfamily binds to the GCAC(A/G)N(A/T)TCCC(A/G)ANG(C/T) element and has an important impact on reproductive organ development and meristem maintenance [126]. In addition to drought and other abiotic stresses, *DREB1C* has recently been implicated in increasing crop yield and nitrogen use efficiency [110]. In our conserved domain analysis, remarkable differences were detected among eight tef and rice homologs including *DREB1C, bZIP-1, CPK21, PIP1-1, PIP2-2, NRT1*, EtbZIP23, *EtPIP1-3*. For example, we observed variation in the size of AP2 domain between *EtDREB1C* and *OsDREB1C*. The AP2 domain in *EtDREB1C* is 30 amino acids compared to the AP2 domain of the rice *DREB1C* which is 61 amino acids long. As shown in the unscaled NJ phylogenetic tree, the tef *DREB1C* underwent a fast substantial evolutionary process when compared to rice (Supplementary Figure 3). With this variation, it is unclear whether the *EtDREB1C* has a conserved physiological function as its rice homolog and requires overexpression or knockout studies to validate its function.

The length of conserved domain of the TF *bZIP-1* in tef was also shorter by six amino acids than the rice homolog (38 amino acids). Due to this difference, the rice *bZIP-1* has an extra binding domain for protein Toc75. Toc75 at the outer envelope of chloroplasts initiates the import of nuclear-encoded proteins from the cytosol into the organelle [127]. In *EtDREB1A*, there are four copies of the AP2 conserved domain in the vicinity of one another within the same transcriptional machinery with a size of 60–68 amino acid while the *OsDREB1A* gene of rice has only one AP2 conserved domain. Our analysis also showed that the length of the substrate binding domain in *EtCPK21* (279 amino acids) is twice that of rice. Unlike the rice homolog, *CPK21* binding domain in tef has an extra binding domain (FRQ1) that was validated to help as Ca^2+^ binding protein. The FRQ1 gene is essential for the growth of budding yeast and a calcium-binding protein [128] but its function in plants is not well characterized. We also observed that the conserved site of *PIP1-1* and *PIP2-2* genes of tef is threefold longer than rice. As mentioned above, these are AQP genes that facilitate the transport of water, glycerol, and small uncharged solutes through the cell membranes [111]. The *NRT1* gene in tef has three copies of the Major Facilitator Superfamily (MSF) protein with single transcriptional machinery, though only one is detected for rice *NRT1*. In addition to drought, *NRT1* is responsible for nitrate uptake and transport, auxin transport, and mediates nitrate-modulated root development [129]. Overall, significant variations were observed in the number size of conserved domains among tef and rice drought-responsive genes, however, the implication of these variations in protein function remains to be understood. Taken together, we identified several candidate genes whose transcript levels respond to drought stress, suggesting their involvement in drought stress responses in tef, however, further research will validate their physiological functions.

## Conclusion

In this study, we performed an *in-silico* analysis and identified 20 potential drought responsive genes in the tef genome that showed high homology with those previously reported in Arabidopsis, rice, maize, sorghum, barley, wheat and pearl millet. We used gene ontology functional enrichment analysis and KEGG pathway analysis to refine promising genes. Out of 253 genes and gene elements identified in the tef genome, we refined 20 top genes with highest enrichment score and statistical indices including kappa coefficient, FDR, and *p*-value. We also performed qPCR to validate the expression of the candidate drought-responsive genes. We found that 14 of 16 genes analyzed were differentially expressed in root, shoot or both tissues in response to drought stress, suggesting their potential role in drought stress responses. However, none of the tef genes analyzed in this study have been isolated and functionally characterized. Therefore, there is a need to functionally characterize these genes in model as well as crop plants. Genes that confer drought resistant under wet lab research are candidates for future drought mitigation programs through molecular breeding approaches.

### Supplementary Information

**Additional file 1: Supplementary Figure 1.** Mapping pattern of 253 orthologous genes and gene elements on the tef genome (colors represent genes and same color on different chromosome represent existence of gene copy. A gene has copy element on several chromosomes (1A/1B, 2A/2B, 3A/3B, 4A/4B, 5A/5B, 6A/6B, 7A/7B, 8A/8B, 9A/9B, 10A/10B and contings_123, 124, 251, 471 and 765) with uneven distribution). **Supplementary Figure 2.** GO analysis of functionally annotated ESTSs in biological process (Fig. 2A) and molecular function (Fig. 2B) using Blast2GO. Bars represent the number of genes in each functional category. **Supplementary Figure 3.** Scaled NJ tree of 20 CDS of genes with high-level GO term compared across four crop plants. The multiple sequence alignment was conducted by CLUSTALW and the Phylogenetic tree was constructed by the Neighbor Joining (NJ) algorithm with default parameters and 1000 bootstrap replication. Genes were grouped into three distinct clusters (I, II, III). Et, Os, Si, and Ec indicates Eragrostis tef, Oryza sativa, Seteria italica and Eleusine coracana respectively.

**Additional file 2: Supplementary Table 1.** Lists of 505 ESTs mapped on teff and submitted to Blast2GO. **Supplementary Table 2.** Lists of 224 genes submitted to DAVID for GO analysis. **Supplementary Table 3.** Lists of 29 ESTs annotated using Blast2GO. **Supplementary Table 4.** 160 genes with Gene ontology term for molecular function. **Supplementary Table 5.** 130 genes with Gene ontology term for biological process. **Supplementary Table 6.** 87 Genes with known GO terms enriched in different biological processes using UP_KW_BIOLOGICAL_PROCESS. **Supplementary Table 7.** 102 Genes with known GO terms enriched in different molecular function using UP_KW_MOLECULAR_FUNCTION. **Supplementary Table 8.** UP_tissue report of gene expression analysis. **Supportive Table 9.** List of genes highly enrich in biological process using ShniyGO. **Supplementary Table 10.** Genes significantly enriched in different molecular Function using shinyGO and using Arabidopsis model. **Supplementary Table 11.** Cluster of genes using gene set enrichment score in several molecular function. **Supplementary Table 12.** Cluster of genes having critical function in several molecular function using Kappa score. **Supplementary Table 13 A.** Genes involved in several biological pathways using KEGG pathway enrichment analysis. **Supplementary Table 13 B.** Pathway analysis using KEGG with ShinyGO. **Supplementary Table 14.** Genes grouped by functional categories defined by high-level GO terms.

## Data Availability

All data used in this manuscript are included in the main manuscript or included as supplementary data files.
