# Correction: Comprehensive *in* *silico* analysis of the underutilized crop tef (*Eragrostis tef* (Zucc.) Trotter) genome reveals drought tolerance signatures

**DOI:** 10.1186/s12870-023-04601-4

**Published:** 2023-11-15

**Authors:** Abreham Bekele‑Alemu, Ayalew Ligaba‑Osena

**Affiliations:** Laboratory of Plant Molecular Biology and Biotechnology, Department of Biology, University of North Carolina Greensboro, Greensboro, NC USA

**Correction:** ***BMC Plant Biol*** **23, 506 (2023)**

**https://doi.org/10.1186/s12870-023-04515-1**

Following publication of the original article [1], author spotted errors in Fig. 8. Revised Fig. 8 was mistakenly not provided during production process. The correct figure is given below:

**Fig. 8:**
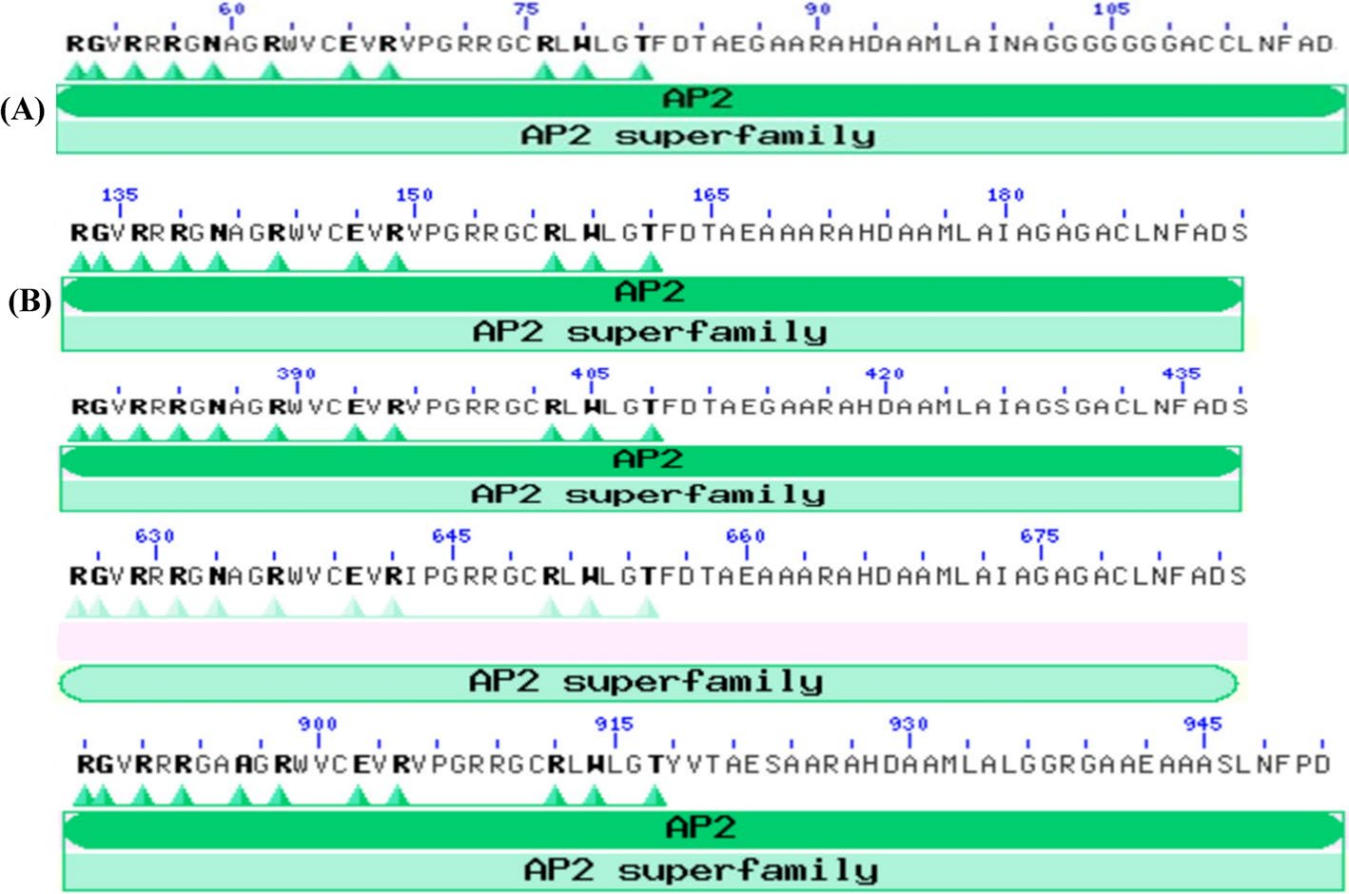
Conserved domain analysis of selected genes. **A** Rice DREB1A with single AP2 binding site, and **B** Tef DREB1A with four structurally related copes within single transcriptional machinery. The amino acids in bold are predicted significant binding sites

The corrections do not affect the overall result or conclusion of the article. The original article has been corrected.
