# Supplementary material for: Comprehensive in silico analysis of the underutilized crop tef (Eragrostis tef (Zucc.) Trotter) genome reveals drought tolerance signatures
Source: BMC Plant Biol. 2023 Oct 21;23:506. doi: 10.1186/s12870-023-04515-1 (PMC10589971; doi:10.1186/s12870-023-04515-1)
Supplement: Supplementary file 1 — Additional file 1: Supplementary Figure 1. Mapping pattern of 253 orthologous genes and gene elements on the tef genome (colors represent genes and same color on different chromosome represent existence of gene copy. A gene has copy element on several chromosomes (1A/1B, 2A/2B, 3A/3B, 4A/4B, 5A/5B, 6A/6B, 7A/7B, 8A/8B, 9A/9B, 10A/10B and contings_123, 124, 251, 471 and 765) with uneven distribution). Supplementary Figure 2. GO analysis of functionally annotated ESTSs in biological process (Fig. 2A) and molecular function (Fig. 2B) using Blast2GO. Bars represent the number of genes in each functional category. Supplementary Figure 3. Scaled NJ tree of 20 CDS of genes with high-level GO term compared across four crop plants. The multiple sequence alignment was conducted by CLUSTALW and the Phylogenetic tree was constructed by the Neighbor Joining (NJ) algorithm with default parameters and 1000 bootstrap replication. Genes were grouped into three distinct clusters (I, II, III). Et, Os, Si, and Ec indicates Eragrostis tef, Oryza sativa, Seteria italica and Eleusine coracana respectively. [file 12870_2023_4515_MOESM1_ESM.docx]

**List of Supplementary figures**


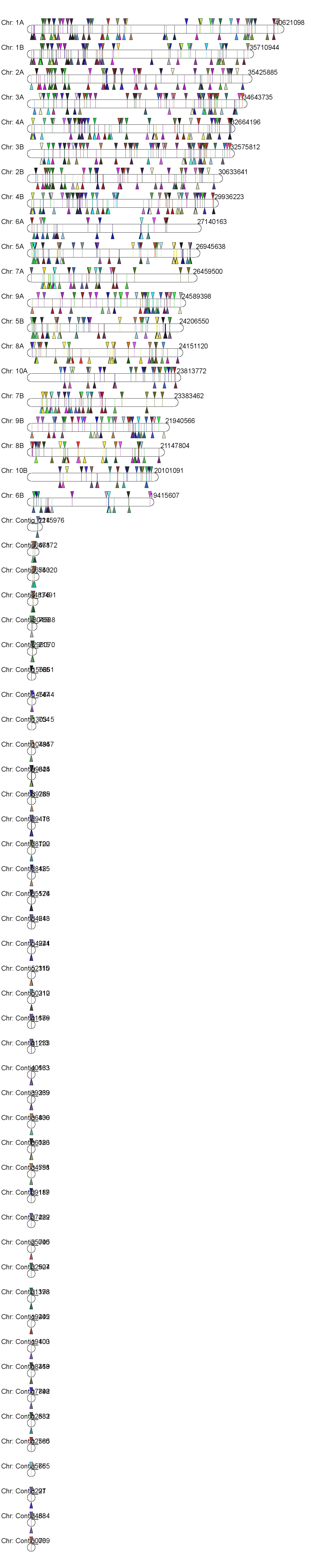


**Supplementary Figure: 1.** Mapping pattern of 253 orthologous genes and gene elements on the tef genome (colors represent genes and same color on different chromosome represent existence of gene copy. A gene has copy element on several chromosomes (1A/1B, 2A/2B, 3A/3B, 4A/4B, 5A/5B, 6A/6B, 7A/7B, 8A/8B, 9A/9B, 10A/10B and contings_123, 124, 251, 471 and 765) with uneven distribution).


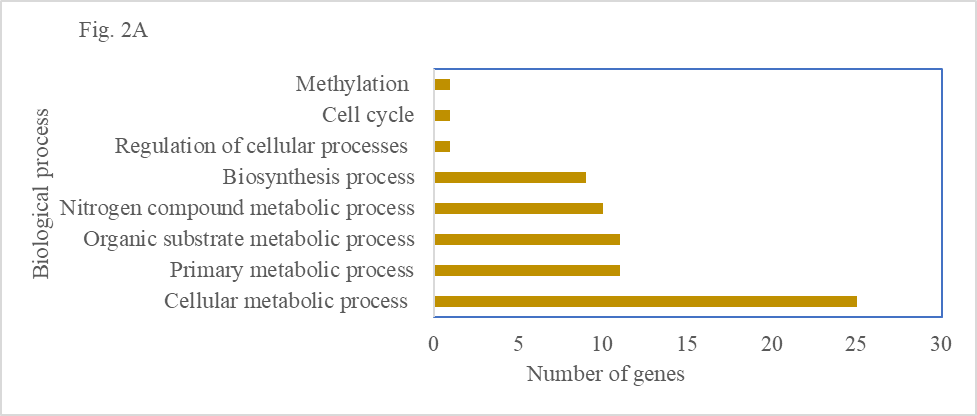

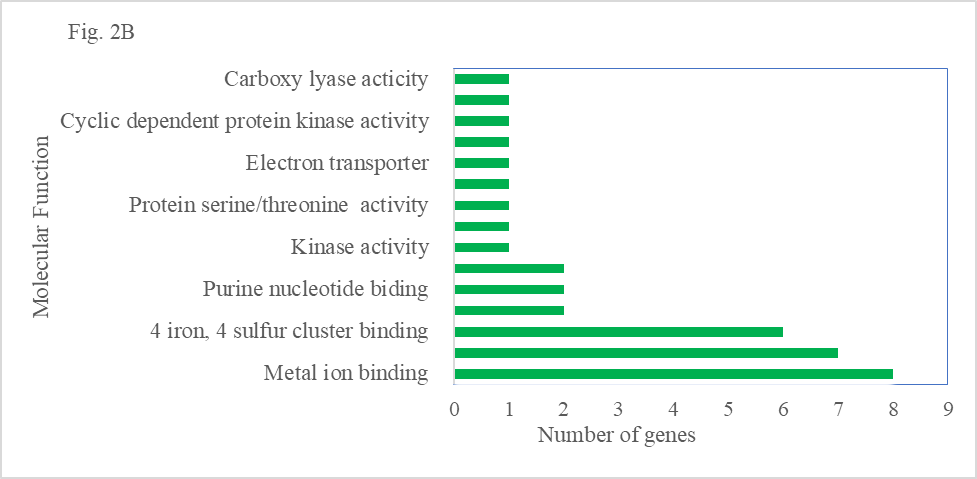


**Supplementary Figure: 2**. GO analysis of functionally annotated ESTSs in biological process (Fig 2A) and molecular function (Fig 2B) using Blast2GO. Bars represent the number of genes in each functional category.


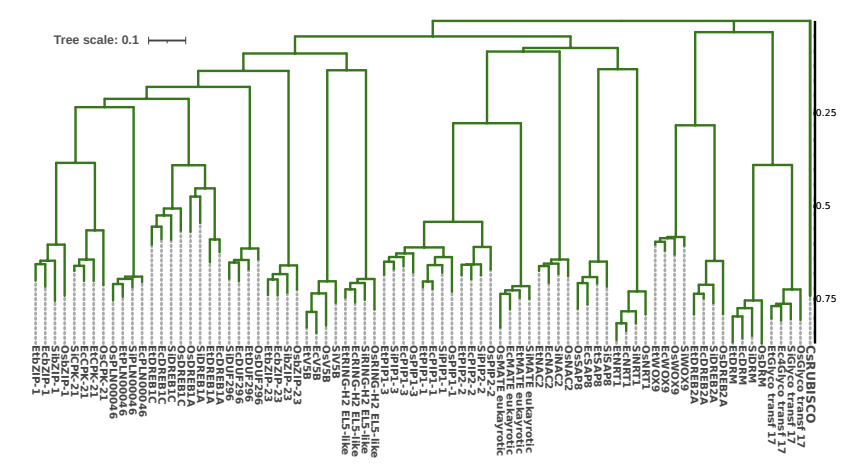


**Supplementary figure 3.** Scaled NJ tree of 20 CDS of genes with high-level GO term compared across four crop plants. The multiple sequence alignment was conducted by CLUSTALW and the Phylogenetic tree was constructed by the Neighbor Joining (NJ) algorithm with default parameters and 1000 bootstrap replication. Genes were grouped into three distinct clusters (I, II, III). Et, Os, Si, and Ec indicates *Eragrostis tef, Oryza sativa, Seteria italica* and *Eleusine coracana* respectively.
