# Supplementary material for: Comprehensive in silico analysis of the underutilized crop tef (Eragrostis tef (Zucc.) Trotter) genome reveals drought tolerance signatures
Source: BMC Plant Biol. 2023 Oct 21;23:506. doi: 10.1186/s12870-023-04515-1 (PMC10589971; doi:10.1186/s12870-023-04515-1)
Supplement: Supplementary file 2 — Additional file 2: Supplementary Table 1. Lists of 505 ESTs mapped on teff and submitted to Blast2GO. Supplementary Table 2. Lists of 224 genes submitted to DAVID for GO analysis. Supplementary Table 3. Lists of 29 ESTs annotated using Blast2GO. Supplementary Table 4. 160 genes with Gene ontology term for molecular function. Supplementary Table 5. 130 genes with Gene ontology term for biological process. Supplementary Table 6. 87 Genes with known GO terms enriched in different biological processes using UP_KW_BIOLOGICAL_PROCESS. Supplementary Table 7. 102 Genes with known GO terms enriched in different molecular function using UP_KW_MOLECULAR_FUNCTION. Supplementary Table 8. UP_tissue report of gene expression analysis. Supportive Table 9. List of genes highly enrich in biological process using ShniyGO. Supplementary Table 10. Genes significantly enriched in different molecular Function using shinyGO and using Arabidopsis model. Supplementary Table 11. Cluster of genes using gene set enrichment score in several molecular function. Supplementary Table 12. Cluster of genes having critical function in several molecular function using Kappa score. Supplementary Table 13 A. Genes involved in several biological pathways using KEGG pathway enrichment analysis. Supplementary Table 13 B. Pathway analysis using KEGG with ShinyGO. Supplementary Table 14. Genes grouped by functional categories defined by high-level GO terms. [file 12870_2023_4515_MOESM2_ESM.docx]

| **Supplementary Table 1.** Lists of 505 ESTs mapped on teff and submitted to Blast2GO | | | | | | |
| --- | --- | --- | --- | --- | --- | --- |
| LT969835 | LT970009 | LT970135 | LT970293 | LT970488 | LT970565 | LT970643 |
| LT969840 | LT970015 | LT970137 | LT970296 | LT970489 | LT970566 | LT970644 |
| LT969841 | LT970020 | LT970138 | LT970297 | LT970490 | LT970567 | LT970645 |
| LT969842 | LT970021 | LT970139 | LT970298 | LT970491 | LT970568 | LT970647 |
| LT969845 | LT970024 | LT970142 | LT970299 | LT970492 | LT970569 | LT970648 |
| LT969846 | LT970026 | LT970143 | LT970300 | LT970493 | LT970570 | LT970649 |
| LT969849 | LT970028 | LT970144 | LT970304 | LT970494 | LT970571 | LT970650 |
| LT969852 | LT970029 | LT970145 | LT970306 | LT970495 | LT970572 | LT970651 |
| LT969853 | LT970038 | LT970146 | LT970308 | LT970496 | LT970572 | LT970652 |
| LT969854 | LT970039 | LT970147 | LT970309 | LT970497 | LT970573 | LT970653 |
| LT969858 | LT970040 | LT970148 | LT970311 | LT970498 | LT970574 | LT970655 |
| LT969860 | LT970041 | LT970149 | LT970312 | LT970499 | LT970575 | LT970656 |
| LT969864 | LT970042 | LT970151 | LT970315 | LT970500 | LT970576 | LT970657 |
| LT969866 | LT970043 | LT970152 | LT970316 | LT970501 | LT970577 | LT970658 |
| LT969869 | LT970044 | LT970153 | LT970319 | LT970502 | LT970578 | LT970659 |
| LT969870 | LT970045 | LT970154 | LT970322 | LT970503 | LT970580 | LT970661 |
| LT969871 | LT970046 | LT970155 | LT970325 | LT970504 | LT970581 | LT970662 |
| LT969872 | LT970047 | LT970156 | LT970326 | LT970505 | LT970582 | LT970663 |
| LT969873 | LT970048 | LT970157 | LT970327 | LT970506 | LT970583 | LT970664 |
| LT969874 | LT970050 | LT970158 | LT970329 | LT970507 | LT970584 | LT970665 |
| LT969881 | LT970051 | LT970159 | LT970330 | LT970509 | LT970585 | LT970666 |
| LT969885 | LT970052 | LT970161 | LT970331 | LT970510 | LT970586 | LT970668 |
| LT969886 | LT970053 | LT970162 | LT970333 | LT970511 | LT970587\| | LT970669 |
| LT969889 | LT970054 | LT970164 | LT970335 | LT970512 | LT970589 | LT970671 |
| LT969890 | LT970055 | LT970165 | LT970336 | LT970513 | LT970590 | LT970673 |
| LT969891 | LT970056 | LT970166 | LT970338 | LT970514 | LT970591 | LT970673 |
| LT969894 | LT970057 | LT970167 | LT970343 | LT970515 | LT970592 | LT970674 |
| LT969896 | LT970058 | LT970168 | LT970346 | LT970516 | LT970593 | LT970678 |
| LT969898 | LT970059 | LT970169 | LT970347 | LT970517 | LT970594 | LT970682 |
| LT969901 | LT970060 | LT970170 | LT970348 | LT970518 | LT970595 | LT970683 |
| LT969902 | LT970061 | LT970172 | LT970353 | LT970519 | LT970596 | LT970684 |
| LT969903 | LT970062 | LT970179 | LT970359 | LT970520 | LT970597 | LT970685 |
| LT969904 | LT970063 | LT970182 | LT970363 | LT970521 | LT970598 | LT970686 |
| LT969905 | LT970064 | LT970197 | LT970364 | LT970522 | LT970599 | LT970696 |
| LT969906 | LT970067 | LT970202 | LT970367 | LT970523 | LT970600 | LT970698 |
| LT969907 | LT970068 | LT970218 | LT970373 | LT970524 | LT970601 | LT970700 |
| LT969909 | LT970070 | LT970229 | LT970374 | LT970525 | LT970602 | LT970701 |
| LT969911 | LT970074 | LT970230 | LT970377 | LT970526 | LT970603 | LT970703 |
| LT969922 | LT970075 | LT970231 | LT970379 | LT970527 | LT970604 | LT970704 |
| LT969924 | LT970076 | LT970232 | LT970381 | LT970528 | LT970605 | LT970706 |
| LT969927 | LT970077 | LT970233 | LT970382 | LT970529 | LT970606 | LT970707 |
| LT969928 | LT970078 | LT970234 | LT970385 | LT970530 | LT970607 | LT970716 |
| LT969931 | LT970079 | LT970235 | LT970389 | LT970530 | LT970608 | LT970718 |
| LT969934 | LT970080 | LT970236 | LT970391 | LT970531 | LT970609 |  |
| LT969935 | LT970081 | LT970237 | LT970393 | LT970532 | LT970610 |  |
| LT969936 | LT970083 | LT970238 | LT970394 | LT970533 | LT970611 |  |
| LT969938 | LT970084 | LT970239 | LT970401 | LT970534 | LT970612 |  |
| LT969942 | LT970085 | LT970240 | LT970403 | LT970535 | LT970613 |  |
| LT969943 | LT970086 | LT970241 | LT970412 | LT970536 | LT970614 |  |
| LT969945 | LT970087 | LT970242 | LT970415 | LT970537 | LT970615 |  |
| LT969947 | LT970088 | LT970243 | LT970416 | LT970538 | LT970616 |  |
| LT969948 | LT970096 | LT970244 | LT970417 | LT970538 | LT970617 |  |
| LT969951 | LT970102 | LT970245 | LT970418 | LT970539 | LT970618 |  |
| LT969953 | LT970110 | LT970246 | LT970420 | LT970540 | LT970619 |  |
| LT969956 | LT970111 | LT970247 | LT970425 | LT970541 | LT970620 |  |
| LT969958 | LT970112 | LT970248 | LT970427 | LT970542 | LT970621 |  |
| LT969959 | LT970113 | LT970249 | LT970429 | LT970543 | LT970622 |  |
| LT969960 | LT970114 | LT970250 | LT970430 | LT970544 | LT970623 |  |
| LT969962 | LT970115 | LT970251 | LT970433 | LT970545 | LT970624 |  |
| LT969963 | LT970116 | LT970253 | LT970435 | LT970546 | LT970625 |  |
| LT969964 | LT970117 | LT970254 | LT970436 | LT970547 | LT970626 |  |
| LT969970 | LT970118 | LT970255 | LT970437 | LT970548 | LT970627 |  |
| LT969971 | LT970119 | LT970256 | LT970449 | LT970549 | LT970628 |  |
| LT969976 | LT970120 | LT970257 | LT970470 | LT970550 | LT970629 |  |
| LT969977 | LT970121 | LT970259 | LT970471 | LT970551 | LT970630 |  |
| LT969978 | LT970122 | LT970266 | LT970474 | LT970552 | LT970631 |  |
| LT969979 | LT970123 | LT970269 | LT970475 | LT970553 | LT970632 |  |
| LT969980 | LT970124 | LT970271 | LT970476 | LT970554 | LT970633 |  |
| LT969982 | LT970125 | LT970274 | LT970477 | LT970555 | LT970634 |  |
| LT969983 | LT970126 | LT970277 | LT970478 | LT970558 | LT970635 |  |
| LT969986 | LT970127 | LT970278 | LT970481 | LT970559 | LT970636 |  |
| LT969988 | LT970128 | LT970279 | LT970482 | LT970560 | LT970637 |  |
| LT969990 | LT970129 | LT970280 | LT970483 | LT970561 | LT970638 |  |
| LT969995 | LT970130 | LT970285 | LT970484 | LT970561 | LT970639 |  |
| LT969996 | LT970131 | LT970288 | LT970485 | LT970562 | LT970640 |  |
| LT969997 | LT970133 | LT970289 | LT970486 | LT970563 | LT970641 |  |
| LT970001 | LT970134 | LT970290 | LT970487 | LT970564 | LT970642 |  |

**Supplementary Table 2.** Lists of 224 genes submitted to DAVID for GO analysis.

| SN | **Gene symbol** | **Gene ID** | **Species** | SN | **Gene symbol** | **Gene ID** | **Species** |
| --- | --- | --- | --- | --- | --- | --- | --- |
| **1** | LOC4344339 | 4344339 | *Oryza sativa Japonica Group* | **113** | *TPS1* | 844194 | *Arabidopsis thaliana* |
| **2** | LOC4343803 | 4343803 | *Oryza sativa Japonica Group* | **114** | *GOLS2* | 842114 | *Arabidopsis thaliana* |
| **3** | LOC4330727 | 4330727 | *Oryza sativa Japonica Group* | **115** | *GOLS1* | 819331 | *Arabidopsis thaliana* |
| **4** | LOC4328332 | 4328332 | *Oryza sativa Japonica Group* | **116** | *NADP-ME* | 4326769 | *Oryza sativa Japonica Group* |
| **5** | LOC4327168 | 4327168 | *Oryza sativa Japonica Group* | **117** | *SNAC1* | 4334553 | *Oryza sativa Japonica Group* |
| **6** | LOC4332957 | 4332957 | *Oryza sativa Japonica Group* | **118** | *OsPUP7* | 4339571 | *Oryza sativa Japonica Group* |
| **7** | LOC4345581 | 4345581 | *Oryza sativa Japonica Group* | **119** | *OsDREB1A* | 4347620 | *Oryza sativa Japonica Group* |
| **8** | LOC4344172 | 4344172 | *Oryza sativa Japonica Group* | **120** | *AP37* | [8082391](https://www.ncbi.nlm.nih.gov/gene/8082391) | *Sorghum bicolor* |
| **9** | LOC4337526 | 4337526 | *Oryza sativa Japonica Group* | **121** | *ERF26* | 4339974 | *Oryza sativa* |
| **10** | LOC4332352 | 4332352 | *Oryza sativa Japonica Group* | **122** | *PIP1-3* | 4331194 | *Oryza sativa* |
| **11** | LOC4333169 | 4333169 | *Oryza sativa Japonica Group* | **123** | *MRP4* | 100125659 | *Zea mays* |
| **12** | LOC4331586 | 4331586 | *Oryza sativa Japonica Group* | **124** | *CIPK03* | 9639733 | *Arabidopsis thaliana* |
| **13** | LOC4337721 | 4337721 | *Oryza sativa Japonica Group* | **125** | *clcC* | 8620286 | *Dictyostelium discoideum* |
| **14** | LOC4340300 | 4340300 | *Oryza sativa Japonica Group* | **126** | *SRK2E* | 829541 | *Arabidopsis thaliana* |
| **15** | LOC9270608 | 9270608 | *Oryza sativa Japonica Group* | **127** | *LOC4339974* | 4339974 | *Oryza sativa Japonica Group* |
| **16** | LOC4346328 | 4346328 | *Oryza sativa Japonica Group* | **128** | *FAR1* | 827173 | *Arabidopsis thaliana* |
| **17** | LOC4349916 | 4349916 | *Oryza sativa Japonica Group* | **129** | *CIPK12* | 827604 | *Arabidopsis thaliana* |
| **18** | LOC4337170 | 4337170 | *Oryza sativa Japonica Group* | **130** | *FAR1* | 832311 | *Arabidopsis thaliana* |
| **19** | LOC4328582 | 4328582 | *Oryza sativa Japonica Group* | **131** | *NCED3* | 820667 | *Arabidopsis thaliana* |
| **20** | LOC4324159 | 4324159 | *Oryza sativa Japonica Group* | **132** | *ZMNF-YB2* | 542390 | *Zea mays* |
| **21** | LOC112938227 | 112938227 | *Oryza sativa Japonica Group* | **133** | *ABCG25* | 843527 | *Arabidopsis thaliana* |
| **22** | LOC4340585 | 4340585 | *Oryza sativa Japonica Group* | **134** | *NCP1* | 100192929 | *Zea mays* |
| **23** | LOC4346248 | 4346248 | *Oryza sativa Japonica Group* | **135** | *PIP2-2* | 123404010 | *Hordeum vulgare* |
| **24** | LOC4349117 | 4349117 | *Oryza sativa Japonica Group* | **136** | *EIN2; MHZ7* | 831889 | *oryza sativa Japonica Group* |
| **25** | LOC4328666 | 4328666 | *Oryza sativa Japonica Group* | **137** | *NAC2A* | 606326 | *Triticum aestivum* |
| **26** | LOC4324110 | 4324110 | *Oryza sativa Japonica Group* | **138** | *BZIP23* | 4330838 | *Oryza sativa Japonica Group* |
| **27** | LOC4330225 | 4330225 | *Oryza sativa Japonica Group* | **139** | *OsDREB2A* | 4324418 | *Oryza sativa Japonica Group* |
| **28** | LOC4347892 | 4347892 | *Oryza sativa Japonica Group* | **140** | *OsEIN2* | 4342431 | *Oryza sativa Japonica Group* |
| **29** | LOC4335831 | 4335831 | *Oryza sativa Japonica Group* | **141** | *OsSAP8* | 4341520 | *Oryza sativa Japonica Group* |
| **30** | LOC4336249 | 4336249 | *Oryza sativa Japonica Group* | **142** | *ACS6* | 826730 | *Arabidopsis thaliana* |
| **31** | LOC4335799 | 4335799 | *Oryza sativa Japonica Group* | **143** | *CDPK2* | 840471 | *Arabidopsis thaliana* |
| **32** | LOC4329918 | 4329918 | *Oryza sativa Japonica Group* | **144** | *OsPIP2;2* | 4330049 | *Oryza sativa Japonica Group* |
| **33** | LOC4344714 | 4344714 | *Oryza sativa Japonica Group* | **145** | *P0455F03* | 4342446 | *Oryza sativa Japonica Group* |
| **34** | LOC4344999 | 4344999 | *Oryza sativa Japonica Group* | **146** | *PIP1-1* | 4330248 | *Oryza sativa Japonica Group* |
| **35** | LOC4339442 | 4339442 | *Oryza sativa Japonica Group* | **147** | XM_008657758 | 103635265 | *Zea mays* |
| **36** | LOC4350916 | 4350916 | *Oryza sativa Japonica Group* | **148** | NM_001352263 | 100193672 | *Zea mays* |
| **37** | LOC4349805 | 4349805 | *Oryza sativa Japonica Group* | **149** | XM_021460037 | 8077682 | *Sorghum bicolor* |
| **38** | LOC4350717 | 4350717 | *Oryza sativa Japonica Group* | **150** | XM_002466823 | 8062999 | *Sorghum bicolor* |
| **39** | LOC4347825 | 4347825 | *Oryza sativa Japonica Group* | **151** | XM_021464912 | 110436990 | *Sorghum bicolor* |
| **40** | LOC4324824 | 4324824 | *Oryza sativa Japonica Group* | **152** | XR_002454657 | 8059975 | *Sorghum bicolor* |
| **41** | LOC4342173 | 4342173 | *Oryza sativa Japonica Group* | **153** | XM_008660885 | 103637861 | *Zea mays* |
| **42** | LOC4330628 | 4330628 | *Oryza sativa Japonica Group* | **154** | XM_002461491 | 8060733 | *Sorghum bicolor* |
| **43** | LOC4327340 | 4327340 | *Oryza sativa Japonica Group* | **155** | XM_008664969 | 103641629 | *Zea mays* |
| **44** | LOC4343863 | 4343863 | *Oryza sativa Japonica Group* | **156** | XM_002446899 | 8057594 | *Sorghum bicolor* |
| **45** | LOC4344030 | 4344030 | *Oryza sativa Japonica Group* | **157** | XM_021456720 | 110433856 | *Sorghum bicolor* |
| **46** | LOC4332538 | 4332538 | *Oryza sativa Japonica Group* | **158** | XM_002454110 | 8072447 | *Sorghum bicolor* |
| **47** | LOC4331362 | 4331362 | *Oryza sativa Japonica Group* | **159** | NM_001152497 | 100279496 | *Zea mays* |
| **48** | LOC4332731 | 4332731 | *Oryza sativa Japonica Group* | **160** | XM_002437356 | 8058459 | *Sorghum bicolor* |
| **49** | LOC4333501 | 4333501 | *Oryza sativa Japonica Group* | **161** | XM_002467903 | 8083705 | *Sorghum bicolor* |
| **50** | LOC4350003 | 4350003 | *Oryza sativa Japonica Group* | **162** | XM_002467664 | 8080431 | *Sorghum bicolor* |
| **51** | LOC4338289 | 4338289 | *Oryza sativa Japonica Group* | **163** | XM_021451749 | 110431928 | *Sorghum bicolor* |
| **52** | LOC4340325 | 4340325 | *Oryza sativa Japonica Group* | **164** | NM_001147384 | 100272932 | *Zea mays* |
| **53** | LOC4325828 | 4325828 | *Oryza sativa Japonica Group* | **165** | XM_021451229 | 8082122 | *Sorghum bicolor* |
| **54** | LOC4345657 | 4345657 | *Oryza sativa Japonica Group* | **166** | XM_021460968 | 8058764 | *Sorghum bicolor* |
| **55** | LOC4336181 | 4336181 | *Oryza sativa Japonica Group* | **167** | XM_008652899 | 100274054 | *Zea mays* |
| **56** | LOC107278728 | 107278728 | *Oryza sativa Japonica Group* | **168** | XM_002466006 | 8059227 | *Sorghum bicolor* |
| **57** | LOC4344441 | 4344441 | *Oryza sativa Japonica Group* | **169** | XM_002456566 | 8057776 | *Sorghum bicolor* |
| **58** | LOC4347311 | 4347311 | *Oryza sativa Japonica Group* | **170** | XM_021450967 | 8081214 | *Sorghum bicolor* |
| **59** | LOC4342464 | 4342464 | *Oryza sativa Japonica Group* | **171** | XM_002455799 | 8078643 | *Sorghum bicolor* |
| **60** | LOC4326871 | 4326871 | *Oryza sativa Japonica Group* | **172** | XM_021448842 | 110430822 | *Sorghum bicolor* |
| **61** | LOC4329854 | 4329854 | *Oryza sativa Japonica Group* | **173** | XM_002456604 | 8058923 | *Sorghum bicolor* |
| **62** | LOC4331811 | 4331811 | *Oryza sativa Japonica Group* | **174** | XM_002451025 | 8066690 | *Sorghum bicolor* |
| **63** | LOC4352458 | 4352458 | *Oryza sativa Japonica Group* | **175** | XM_021456504 | 8075414 | *Sorghum bicolor* |
| **64** | LOC4346187 | 4346187 | *Oryza sativa Japonica Group* | **176** | XM_008665394 | 103642077 | *Zea mays* |
| **65** | LOC4327316 | 4327316 | *Oryza sativa Japonica Group* | **177** | NM_001350033 | 100383187 | *Zea mays* |
| **66** | LOC4333878 | 4333878 | *Oryza sativa Japonica Group* | **178** | XM_020550699 | 103650094 | *Zea mays* |
| **67** | LOC4339617 | 4339617 | *Oryza sativa Japonica Group* | **179** | XM_021463259 | 8068630 | *Sorghum bicolor* |
| **68** | LOC4349090 | 4349090 | *Oryza sativa Japonica Group* | **180** | XM_002457366 | 8073342 | *Sorghum bicolor* |
| **69** | *AAO3* | 817257 | *Arabidopsis thaliana* | **181** | XM_020546492 | 100273814 | *Zea mays* |
| **70** | *ABCG22* | 830541 | *Arabidopsis thaliana* | **182** | XM_002458456 | 8075588 | *Sorghum bicolor* |
| **71** | *ABI1* | 828714 | *Arabidopsis thaliana* | **183** | XM_002466035 | 8060854 | *Sorghum bicolor* |
| **72** | *ABO1* | 831213 | *Arabidopsis thaliana* | **184** | NM_001291654 | 100274836 | *Zea mays* |
| **73** | *APX2* | 820121 | *Arabidopsis thaliana* | **185** | XM_002451316 | 8066485 | *Sorghum bicolor* |
| **74** | *BGLU18* | 841670 | *Arabidopsis thaliana* | **186** | XM_008663486 | 100275707 | *Zea mays* |
| **75** | *RBOHD* | 834842 | *Arabidopsis thaliana* | **187** | XM_021454032 | 8078579 | *Sorghum bicolor* |
| **76** | *RBOHF* | 842710 | *Arabidopsis thaliana* | **188** | XM_002459271 | 8059924 | *Sorghum bicolor* |
| **77** | *AVP1* | 838138 | *Arabidopsis thaliana* | **189** | BT066249 | 100284805 | *Zea mays* |
| **78** | *CBF1* | 828653 | *Arabidopsis thaliana* | **190** | EU965224 | 100275447 | *Zea mays* |
| **79** | *CBP20* | 834443 | *Arabidopsis thaliana* | **191** | EU953873 | 100381320 | *Zea mays* |
| **80** | *CIPK15* | 830556 | *Arabidopsis thaliana* | **192** | BT040247 | 100217031 | *Zea mays* |
| **81** | *CYP707A1* | 827663 | *Arabidopsis thaliana* | **193** | EU969852 | 103643967 | *Zea mays* |
| **82** | *CYP707A3* | 834570 | *Arabidopsis thaliana* | **194** | EU963465 | 100283536 | *Zea mays* |
| **83** | *CBF2* | 828651 | *Arabidopsis thaliana* | **195** | EU952538 | 103649807 | *Zea mays* |
| **84** | *DREB2A* | 830424 | *Arabidopsis thaliana* | **196** | BT041074 | 100283330 | *Zea mays* |
| **85** | *HDG11* | 843671 | *Arabidopsis thaliana* | **197** | AY105703 | 100274751 | *Zea mays* |
| **86** | *EIN2* | 831889 | *Arabidopsis thaliana* | **198** | EU969275 | 100857041 | *Zea mays* |
| **87** | *ELO1* | 820292 | *Arabidopsis thaliana* | **199** | 819323 | 819323 | *Arabidopsis thaliana* |
| **88** | *FHY3* | 821781 | *Arabidopsis thaliana* | **200** | 831241 | 831241 | *Arabidopsis thaliana* |
| **89** | *GPX3* | 818936 | *Arabidopsis thaliana* | **201** | 841514 | 841514 | *Arabidopsis thaliana* |
| **90** | *ERD9* | 837576 | *Arabidopsis thaliana* | **202** | 839226 | 839226 | *Arabidopsis thaliana* |
| **91** | *HD1* | 829969 | *Arabidopsis thaliana* | **203** | EU968834.1 | 195642967 | *Zea mays* |
| **92** | *HDA6* | 836431 | *Arabidopsis thaliana* | **204** | XM_002440106 | 8061071 | *Sorghum bicolor* |
| **93** | *PKT3* | 817876 | *Arabidopsis thaliana* | **205** | XM_002443727 | 8068578 | *Sorghum bicolor* |
| **94** | *MYBR1* | 836865 | *Arabidopsis thaliana* | **206** | XM_002466569 | 8064218 | *Sorghum bicolor* |
| **95** | *MYB60* | 837403 | *Arabidopsis thaliana* | **207** | XM_021461852 | 8083691 | *Sorghum bicolor* |
| **96** | *MYC2* | 840158 | *Arabidopsis thaliana* | **208** | XM_002463695 | 8061169 | *Sorghum bicolor* |
| **97** | *OST1* | 829541 | *Arabidopsis thaliana* | **209** | XM_002437420 | 8061631 | *Sorghum bicolor* |
| **98** | *HA1* | 816413 | *Arabidopsis thaliana* | **210** | XM_002460388 | 8055854 | *Sorghum bicolor* |
| **99** | *PIP1A* | 825316 | *Arabidopsis thaliana* | **211** | XM_021461536 | 8076352 | *Sorghum bicolor* |
| **100** | *PIP2A* | 824510 | *Arabidopsis thaliana* | **212** | XM_002460398 | 8055864 | *Sorghum bicolor* |
| **101** | *RCAR1* | 838452 | *Arabidopsis thaliana* | **213** | XM_002467023 | 8067406 | *Sorghum bicolor* |
| **102** | *RPK1* | 843258 | *Arabidopsis thaliana* | **214** | XM_021446311 | 110429775 | *Sorghum bicolor* |
| **103** | *XF1* | 842213 | *Arabidopsis thaliana* | **215** | XM_002452914 | 8082723 | *Sorghum bicolor* |
| **104** | *SNRK2-8* | 844164 | *Arabidopsis thaliana* | **216** | XM_021447255 | 8085105 | *Sorghum bicolor* |
| **105** | *WRKY2* | 835726 | *Arabidopsis thaliana* | **217** | XM_021447373 | 8063611 | *Sorghum bicolor* |
| **106** | *ABI2* | 835809 | *Arabidopsis thaliana* | **218** | LOC8081192 | 8081192 | *Sorghum bicolor* |
| **107** | *KAT2* | 827555 | *Arabidopsis thaliana* | **219** | XM_021452107 | 110432163 | *Sorghum bicolor* |
| **108** | *CPK21* | 825807 | *Arabidopsis thaliana* | **220** | XM_002448951 | 8058534 | *Sorghum bicolor* |
| **109** | *ERD1* | 835180 | *Arabidopsis thaliana* | **221** | XM_002442069 | 8061256 | *Sorghum bicolor* |
| **110** | *CPK23* | 825809 | *Arabidopsis thaliana* | **222** | NM_001154000 | 100281081 | *Zea mays* |
| **111** | *RCAR1* | 838452 | *Arabidopsis thaliana* | **223** | XM_002450091 | 8067611 | *Sorghum bicolor* |
| **112** | *HDG11* | 843671 | *Arabidopsis thaliana* | **224** | XM_002456291 | 8062168 | *Sorghum bicolor* |

| **Supplementary Table 3.** Lists of 29 ESTs annotated using Blast2GO | | | | | | | |
| --- | --- | --- | --- | --- | --- | --- | --- |
| SN | ENA ACC number | Description |  | Length | e-Value | sim mean | GO Names |
| 1 | LT970347 | protein-L-isoaspartate O-methyltransferase 1-like isoform X2 | *Setaria viridis* | 438 | 1.50E-79 | 98.4 | P:protein methylation; F:protein-L-isoaspartate (D-aspartate) O-methyltransferase activity |
| 2 | LT969835 | photosystem I chlorophyll a apoprotein A2 | *odestobacter lapidis* | 516 | 1.40E-117 | 100 | P:photosynthesis; F:chlorophyll binding; F:oxidoreductase activity; F:metal ion binding; F:4 iron, 4 sulfur cluster binding; C:chloroplast; C:photosystem I; C:integral component of membrane |
| 3 | LT969842 | photosystem I chlorophyll a apoprotein A2 | *Modestobacter lapidis* | 516 | 6.16E-117 | 100 | P:photosynthesis; F:chlorophyll binding; F:oxidoreductase activity; F:metal ion binding; F:4 iron, 4 sulfur cluster binding; C:chloroplast; C:photosystem I; C:integral component of membrane |
| 4 | LT969891 | photosystem I chlorophyll a apoprotein A2 | *Modestobacter lapidis* | 516 | 1.40E-117 | 100 | P:photosynthesis; F:chlorophyll binding; F:oxidoreductase activity; F:metal ion binding; F:4 iron, 4 sulfur cluster binding; C:chloroplast; C:photosystem I; C:integral component of membrane |
| 5 | LT969943 | hypothetical protein ERO13_D08G129740v2 | *Gossypium hirsutum* | 546 | 3.74E-103 | 100 | P:photosynthesis; F:chlorophyll binding; F:oxidoreductase activity; F:metal ion binding; F:4 iron, 4 sulfur cluster binding; C:chloroplast; C:photosystem I; C:integral component of membrane |
| 6 | LT969977 | photosystem I chlorophyll a apoprotein A2 | *Modestobacter lapidis* | 516 | 1.40E-117 | 100 | P:photosynthesis; F:chlorophyll binding; F:oxidoreductase activity; F:metal ion binding; F:4 iron, 4 sulfur cluster binding; C:chloroplast; C:photosystem I; C:integral component of membrane |
| 7 | LT970381 | Photosystem I chlorophyll a apoprotein A1 | *Capsicum baccatum* | 321 | 1.11E-70 | 100 | P:photosynthesis; F:chlorophyll binding; F:oxidoreductase activity; F:metal ion binding; F:4 iron, 4 sulfur cluster binding; C:chloroplast; C:photosystem I; C:integral component of membrane |
| 8 | LT969845 | hypothetical protein MIMGU_mgv1a024202mg, partial | *Erythranthe guttata* | 98 | 7.20E-61 | 98.94 | P:photosynthesis; C:thylakoid; C:integral component of membrane |
| 9 | LT969873 | hypothetical protein MIMGU_mgv1a024202mg, partial | *Erythranthe guttata* | 98 | 5.10E-62 | 100 | P:photosynthesis; C:thylakoid; C:integral component of membrane |
| 10 | LT969901 | hypothetical protein C4D60_Mb00t15050 | *Musa balbisiana* | 357 | 1.72E-80 | 100 | P:photosynthesis; C:thylakoid; C:integral component of membrane |
| 11 | LT969903 | hypothetical protein MIMGU_mgv1a024202mg, partial | *Erythranthe guttata* | 98 | 7.77E-63 | 100 | P:photosynthesis; C:thylakoid; C:integral component of membrane |
| 12 | LT969904 | hypothetical protein MIMGU_mgv1a024202mg, partial | *Erythranthe guttata* | 98 | 7.77E-63 | 100 | P:photosynthesis; C:thylakoid; C:integral component of membrane |
| 13 | LT969907 | hypothetical protein MIMGU_mgv1a024202mg, partial | *Erythranthe guttata* | 98 | 2.23E-62 | 100 | P:photosynthesis; C:thylakoid; C:integral component of membrane |
| 14 | LT970394 | TPA_asm: hypothetical protein HUJ06_031949 | *Nelumbo nucifera* | 559 | 8.46E-61 | 100 | P:photosynthesis; C:chloroplast; C:thylakoid; C:integral component of membrane |
| 15 | LT970271 | photosystem II protein D1-like | *[Triticum dicoccoides* | 433 | 1.10E-76 | 90.51 | P:photosynthetic electron transport in photosystem II; F:chlorophyll binding; F:electron transporter, transferring electrons within the cyclic electron transport pathway of photosynthesis activity; F:metal ion binding; C:photosystem II; C:plastid; C:integral component of membrane |
| 16 | LT969906 | hypothetical protein SEVIR_9G044690v2 | *Setaria viridis* | 479 | 8.29E-106 | 98.7 | P:photorespiration; P:reductive pentose-phosphate cycle; F:magnesium ion binding; F:monooxygenase activity; F:ribulose-bisphosphate carboxylase activity; C:chloroplast |
| 17 | LT970374 | cyclin-dependent kinase G-1 | *Setaria viridis* | 280 | 1.19E-54 | 100 | P:protein phosphorylation; P:regulation of mitotic cell cycle; F:cyclin-dependent protein serine/threonine kinase activity; F:ATP binding; C:nucleus |
| 18 | LT969947 | heat shock protein 70, partial | *Ziziphus jujuba* | 372 | 3.61E-77 | 97.93 | F:ATP binding; F:ATP hydrolysis activity |
| 19 | LT969852 | small subunit ribosomal protein S12 | *Vigna unguiculata* | 540 | 3.66E-54 | 97.85 | P:translation; F:structural constituent of ribosome; C:small ribosomal subunit |
| 20 | LT969964 | small subunit ribosomal protein S12 | *Vigna unguiculata* | 602 | 8.35E-52 | 96.74 | P:translation; F:structural constituent of ribosome; C:small ribosomal subunit |
| 21 | LT970038 | small subunit ribosomal protein S12 | *Vigna unguiculata* | 669 | 1.44E-52 | 96.77 | P:translation; F:structural constituent of ribosome; C:small ribosomal subunit |
| 22 | LT970039 | small subunit ribosomal protein S12 | *Vigna unguiculata* | 601 | 3.86E-53 | 96.77 | P:translation; F:structural constituent of ribosome; C:small ribosomal subunit |
| 23 | LT970040 | small subunit ribosomal protein S12 | *Vigna unguiculata* | 689 | 1.25E-52 | 96.77 | P:translation; F:structural constituent of ribosome; C:small ribosomal subunit |
| 24 | LT970041 | small subunit ribosomal protein S12 | *Vigna unguiculata* | 539 | 3.66E-54 | 97.85 | P:translation; F:structural constituent of ribosome; C:small ribosomal subunit |
| 25 | LT970043 | small subunit ribosomal protein S12 | *Vigna unguiculata* | 501 | 2.24E-54 | 97.85 | P:translation; F:structural constituent of ribosome; C:small ribosomal subunit |
| 26 | LT970481 | small subunit ribosomal protein S12 | *Vigna unguiculata* | 568 | 5.11E-54 | 97.85 | P:translation; F:structural constituent of ribosome; C:small ribosomal subunit |
| 27 | LT969898 | uncharacterized protein LOC117863389 | *Setaria viridis* | 720 | 2.33E-131 | 92.33 | C:integral component of membrane |
| 28 | LT970683 | hypothetical protein HU200_002364 | *Digitaria exilis* | 458 | 2.19E-74 | 88.59 | C:integral component of membrane |
| 29 | LT970315 | hypothetical protein PhapfoPp090 | *Phalaenopsis aphrodite* | 308 | 1.45E-55 | 97.73 | C:chloroplast |

| **Supplementary Table 4.** 160 genes with Gene ontology term for molecular function | | | | |
| --- | --- | --- | --- | --- |
| **SN** | **ENTREZ_ID** | **Gene description** | **Species** | **GOTERM_MF_DIRECT** |
| 1 | 826730 | 1-aminocyclopropane-1-carboxylic acid (acc) synthase 6(ACS6) | *Arabidopsis thaliana* | GO:0003824~catalytic activity,GO:0005515~protein binding,GO:0016847~1-aminocyclopropane-1-carboxylate synthase activity,GO:0030170~pyridoxal phosphate binding,GO:0042802~identical protein binding, |
| 2 | 100284805 | 10-deacetylbaccatin III 10-O-acetyltransferase(LOC100284805) | *Zea mays* | GO:0016410~N-acyltransferase activity, |
| 3 | 8073342 | 16.9 kDa class I heat shock protein 1(LOC8073342) | *Sorghum bicolor* | GO:0043621~protein self-association,GO:0051082~unfolded protein binding, |
| 4 | 8058534 | 2-carboxy-D-arabinitol-1-phosphatase(LOC8058534) | *Sorghum bicolor* | GO:0016791~phosphatase activity,GO:0016868~intramolecular transferase activity, phosphotransferases, |
| 5 | 8058459 | 4-hydroxy-tetrahydrodipicolinate synthase, chloroplastic(LOC8058459) | *Sorghum bicolor* | GO:0008840~4-hydroxy-tetrahydrodipicolinate synthase, |
| 6 | 4337526 | 50S ribosomal protein L28, chloroplastic(LOC4337526) | *Oryza sativa Japonica Group* | GO:0003729~mRNA binding,GO:0003735~structural constituent of ribosome, |
| 7 | 8062999 | 60S ribosomal protein L10(LOC8062999) | *Sorghum bicolor* | GO:0003735~structural constituent of ribosome, |
| 8 | 103642077 | 60S ribosomal protein L27-3(LOC103642077) | *Zea mays* | GO:0003735~structural constituent of ribosome, |
| 9 | 100193672 | 60S ribosomal protein L35a-like(LOC100193672) | *Zea mays* | GO:0003735~structural constituent of ribosome, |
| 10 | 100125659 | ABC transporter C family MRP4(LOC100125659) | *Zea mays* | GO:0005524~ATP binding,GO:0042626~ATPase activity, coupled to transmembrane movement of substances, |
| 11 | 8061256 | ABC transporter F family member 1(LOC8061256) | *Sorghum bicolor* | GO:0005524~ATP binding,GO:0042626~ATPase activity, coupled to transmembrane movement of substances, |
| 12 | 8076352 | ABC transporter G family member 11(LOC8076352) | *Sorghum bicolor* | GO:0005524~ATP binding,GO:0042626~ATPase activity, coupled to transmembrane movement of substances, |
| 13 | 830541 | ABC-2 type transporter family protein(ABCG22) | *Arabidopsis thaliana* | GO:0005524~ATP binding,GO:0042626~ATPase activity, coupled to transmembrane movement of substances, |
| 14 | 8077682 | APO protein 2, chloroplastic(LOC8077682) | *Sorghum bicolor* | GO:0003723~RNA binding, |
| 15 | 4344714 | AT-hook motif nuclear-localized protein 23(LOC4344714) | *Oryza sativa Japonica Group* | GO:0003680~AT DNA binding,GO:0003700~transcription factor activity, sequence-specific DNA binding, |
| 16 | 843527 | ATP-binding casette family G25(ABCG25) | *Arabidopsis thaliana* | GO:0005524~ATP binding,GO:0015562~efflux transmembrane transporter activity,GO:0042626~ATPase activity, coupled to transmembrane movement of substances, |
| 17 | 840158 | Basic helix-loop-helix (bHLH) DNA-binding family protein(MYC2) | *Arabidopsis thaliana* | GO:0000976~transcription regulatory region sequence-specific DNA binding,GO:0003677~DNA binding,GO:0003700~transcription factor activity, sequence-specific DNA binding,GO:0005515~protein binding,GO:0019900~kinase binding,GO:0043565~sequence-specific DNA binding,GO:0046983~protein dimerization activity, |
| 18 | 828653 | C-repeat/DRE binding factor 1(CBF1) | *Arabidopsis thaliana* | GO:0003677~DNA binding,GO:0003700~transcription factor activity, sequence-specific DNA binding,GO:0005515~protein binding, |
| 19 | 828651 | C-repeat/DRE binding factor 2(CBF2) | *Arabidopsis thaliana* | GO:0000976~transcription regulatory region sequence-specific DNA binding,GO:0003677~DNA binding,GO:0003700~transcription factor activity, sequence-specific DNA binding,GO:0005515~protein binding, |
| 20 | 542390 | CAAT-box DNA binding protein subunit B (NF-YB)(LOC542390) | *Zea mays* | GO:0000981~RNA polymerase II transcription factor activity, sequence-specific DNA binding,GO:0001228~transcriptional activator activity, RNA polymerase II transcription regulatory region sequence-specific binding,GO:0043565~sequence-specific DNA binding,GO:0046982~protein heterodimerization activity, |
| 21 | 834443 | CAP-binding protein 20(CBP20) | *Arabidopsis thaliana* | GO:0000339~RNA cap binding,GO:0005515~protein binding, |
| 22 | 827604 | CBL-interacting protein kinase 12(CIPK12) | *Arabidopsis thaliana* | GO:0004672~protein kinase activity,GO:0005515~protein binding,GO:0005524~ATP binding, |
| 23 | 830556 | CBL-interacting protein kinase 15(CIPK15) | *Arabidopsis thaliana* | GO:0004672~protein kinase activity,GO:0004674~protein serine/threonine kinase activity,GO:0005515~protein binding,GO:0005524~ATP binding, |
| 24 | 8067611 | CBL-interacting protein kinase 15(LOC8067611) | *Sorghum bicolor* | GO:0005524~ATP binding, |
| 25 | 9639733 | CBL-interacting protein kinase 23(LOC9639733) | *Selaginella moellendorffii* | GO:0005524~ATP binding, |
| 26 | 8620286 | CLC 6/7 family protein(clcC) | *Dictyostelium discoideum AX4* | GO:0005244~voltage-gated ion channel activity,GO:0005247~voltage-gated chloride channel activity,GO:0005254~chloride channel activity,GO:0015108~chloride transmembrane transporter activity, |
| 27 | 831241 | Chalcone and stilbene synthase family protein(TT4) | *Arabidopsis thaliana* | GO:0005515~protein binding,GO:0016210~naringenin-chalcone synthase activity,GO:0016747~transferase activity, transferring acyl groups other than amino-acyl groups, |
| 28 | 835180 | Clp ATPase(ERD1) | *Arabidopsis thaliana* | GO:0005524~ATP binding,GO:0016887~ATPase activity, |
| 29 | 8066485 | DExH-box ATP-dependent RNA helicase DExH12(LOC8066485) | *Sorghum bicolor* | GO:0003676~nucleic acid binding,GO:0003724~RNA helicase activity,GO:0005524~ATP binding,GO:0016787~hydrolase activity, |
| 30 | 830424 | DRE-binding protein 2A(DREB2A) | *Arabidopsis thaliana* | GO:0000976~transcription regulatory region sequence-specific DNA binding,GO:0003677~DNA binding,GO:0003700~transcription factor activity, sequence-specific DNA binding,GO:0005515~protein binding, |
| 31 | 4344172 | E3 ubiquitin-protein ligase DIS1-like(LOC4344172) | *Oryza sativa Japonica Group* | GO:0008270~zinc ion binding,GO:0016740~transferase activity,GO:0061630~ubiquitin protein ligase activity, |
| 32 | 110430822 | E3 ubiquitin-protein ligase RZF1-like(LOC110430822) | *Sorghum bicolor* | GO:0061630~ubiquitin protein ligase activity, |
| 33 | 8083691 | ETHYLENE INSENSITIVE 3-like 1 protein(LOC8083691) | *Sorghum bicolor* | GO:0003677~DNA binding,GO:0003700~transcription factor activity, sequence-specific DNA binding, |
| 34 | 842213 | FAD/NAD(P)-binding oxidoreductase family protein(XF1) | *Arabidopsis thaliana* | GO:0004506~squalene monooxygenase activity,GO:0050660~flavin adenine dinucleotide binding, |
| 35 | 827173 | FRS (FAR1 Related Sequences) transcription factor family(FAR1) | *Arabidopsis thaliana* | GO:0003700~transcription factor activity, sequence-specific DNA binding,GO:0005515~protein binding,GO:0008270~zinc ion binding, |
| 36 | 4335831 | G-type lectin S-receptor-like serine/threonine-protein kinase At2g19130(LOC4335831) | *Oryza sativa Japonica Group* | GO:0005524~ATP binding, |
| 37 | 837576 | Glutathione S-transferase family protein(ERD9) | *Arabidopsis thaliana* | GO:0004364~glutathione transferase activity, |
| 38 | 816413 | H[+]-ATPase 1(HA1) | *Arabidopsis thaliana* | GO:0000287~magnesium ion binding,GO:0005515~protein binding,GO:0005524~ATP binding,GO:0008553~hydrogen-exporting ATPase activity, phosphorylative mechanism,GO:0016887~ATPase activity, |
| 39 | 831213 | IKI3 family protein(ABO1) | *Arabidopsis thaliana* | GO:0000049~tRNA binding,GO:0016746~transferase activity, transferring acyl groups,GO:0043621~protein self-association, |
| 40 | 838138 | Inorganic H pyrophosphatase family protein(AVP1) | *Arabidopsis thaliana* | GO:0003729~mRNA binding,GO:0004427~inorganic diphosphatase activity,GO:0009678~hydrogen-translocating pyrophosphatase activity,GO:0046872~metal ion binding, |
| 41 | 8059924 | L-type lectin-domain containing receptor kinase IV.1(LOC8059924) | *Sorghum bicolor* | GO:0004675~transmembrane receptor protein serine/threonine kinase activity,GO:0005524~ATP binding,GO:0030246~carbohydrate binding, |
| 42 | 4342446 | MD-2-related lipid-recognition protein ROSY1(LOC4342446) | *Oryza sativa Japonica Group* | GO:0032934~sterol binding, |
| 43 | 4334553 | NAC domain-containing protein 2-like(LOC4334553) | *Oryza sativa Japonica Group* | GO:0042803~protein homodimerization activity,GO:0043565~sequence-specific DNA binding, |
| 44 | 4325828 | NAC domain-containing protein 48-like(LOC4325828) | *Oryza sativa Japonica Group* | GO:0003677~DNA binding, |
| 45 | 4326769 | NADP-dependent malic enzyme, chloroplastic-like(LOC4326769) | *Oryza sativa Japonica Group* | GO:0004470~malic enzyme activity,GO:0004471~malate dehydrogenase (decarboxylating) (NAD+) activity,GO:0004473~malate dehydrogenase (decarboxylating) (NADP+) activity,GO:0008948~oxaloacetate decarboxylase activity,GO:0046872~metal ion binding,GO:0051287~NAD binding, |
| 46 | 831889 | NRAMP metal ion transporter family protein(EIN2) | *Arabidopsis thaliana* | GO:0003682~chromatin binding,GO:0003729~mRNA binding,GO:0005384~manganese ion transmembrane transporter activity,GO:0005515~protein binding,GO:0015086~cadmium ion transmembrane transporter activity,GO:0042393~histone binding, |
| 47 | 841514 | Nucleolar GTP-binding protein(AT1G50920) | *Arabidopsis thaliana* | GO:0003729~mRNA binding,GO:0005525~GTP binding, |
| 48 | 820292 | Paxneb protein-like protein(ELO1) | *Arabidopsis thaliana* | GO:0005515~protein binding,GO:0016746~transferase activity, transferring acyl groups, |
| 49 | 829541 | Protein kinase superfamily protein(OST1) | *Arabidopsis thaliana* | GO:0004672~protein kinase activity,GO:0004674~protein serine/threonine kinase activity,GO:0005515~protein binding,GO:0005524~ATP binding,GO:0009931~calcium-dependent protein serine/threonine kinase activity,GO:0016301~kinase activity,GO:0019903~protein phosphatase binding,GO:0042802~identical protein binding, |
| 50 | 844164 | Protein kinase superfamily protein(SNRK2-8) | *Arabidopsis thaliana* | GO:0004674~protein serine/threonine kinase activity,GO:0005524~ATP binding, |
| 51 | 828714 | Protein phosphatase 2C family protein(ABI1) | *Arabidopsis thaliana* | GO:0004721~phosphoprotein phosphatase activity,GO:0004722~protein serine/threonine phosphatase activity,GO:0005509~calcium ion binding,GO:0005515~protein binding,GO:0016791~phosphatase activity,GO:0017018~myosin phosphatase activity,GO:0019900~kinase binding,GO:0019901~protein kinase binding,GO:0046872~metal ion binding, |
| 52 | 835809 | Protein phosphatase 2C family protein(ABI2) | *Arabidopsis thaliana* | GO:0004722~protein serine/threonine phosphatase activity,GO:0005515~protein binding,GO:0017018~myosin phosphatase activity,GO:0046872~metal ion binding, |
| 53 | 4347892 | R3H domain-containing protein 1(LOC4347892) | *Oryza sativa Japonica Group* | GO:0003676~nucleic acid binding, |
| 54 | 4340585 | RING-H2 finger protein ATL46(LOC4340585) | *Oryza sativa Japonica Group* | GO:0031625~ubiquitin protein ligase binding, |
| 55 | 4332957 | RNA-binding protein CP29B, chloroplastic(LOC4332957) | *Oryza sativa Japonica Group* | GO:0003729~mRNA binding, |
| 56 | 110431928 | TOM1-like protein 2(LOC110431928) | *Sorghum bicolor* | GO:0035091~phosphatidylinositol binding,GO:0043130~ubiquitin binding, |
| 57 | 4330628 | U-box domain-containing protein 11(LOC4330628) | *Oryza sativa Japonica Group* | GO:0004842~ubiquitin-protein transferase activity, |
| 58 | 110436990 | UDP-glucose 6-dehydrogenase 5(LOC110436990) | *Sorghum bicolor* | GO:0003979~UDP-glucose 6-dehydrogenase activity,GO:0016628~oxidoreductase activity, acting on the CH-CH group of donors, NAD or NADP as acceptor,GO:0051287~NAD binding, |
| 59 | 100274751 | UDP-glucuronate 4-epimerase 1(LOC100274751) | *Zea mays* | GO:0003824~catalytic activity, |
| 60 | 835726 | WRKY DNA-binding protein 2(WRKY2) | *Arabidopsis thaliana* | GO:0000976~transcription regulatory region sequence-specific DNA binding,GO:0003700~transcription factor activity, sequence-specific DNA binding,GO:0005515~protein binding,GO:0043565~sequence-specific DNA binding,GO:0046872~metal ion binding, |
| 61 | 4324824 | WUSCHEL-related homeobox 9-like(LOC4324824) | *Oryza sativa Japonica Group* | GO:0003677~DNA binding,GO:0003700~transcription factor activity, sequence-specific DNA binding, |
| 62 | 817257 | abscisic aldehyde oxidase 3(AAO3) | *Arabidopsis thaliana* | GO:0004031~aldehyde oxidase activity,GO:0005506~iron ion binding,GO:0010293~abscisic aldehyde oxidase activity,GO:0016491~oxidoreductase activity,GO:0031625~ubiquitin protein ligase binding,GO:0050302~indole-3-acetaldehyde oxidase activity,GO:0051537~2 iron, 2 sulfur cluster binding,GO:0071949~FAD binding, |
| 63 | 4331194 | aquaporin PIP 1-3-like(LOC4331194) | *Oryza sativa Japonica Group* | GO:0015250~water channel activity, |
| 64 | 4330248 | aquaporin PIP1-1-like(LOC4330248) | *Oryza sativa Japonica Group* | GO:0015250~water channel activity,GO:0015267~channel activity, |
| 65 | 820121 | ascorbate peroxidase 2(APX2) | *Arabidopsis thaliana* | GO:0004601~peroxidase activity,GO:0016688~L-ascorbate peroxidase activity,GO:0020037~heme binding,GO:0046872~metal ion binding, |
| 66 | 8067406 | auxin response factor 22(LOC8067406) | *Sorghum bicolor* | GO:0003677~DNA binding, |
| 67 | 4330838 | bZIP transcription factor 23-like(LOC4330838) | *Oryza sativa Japonica Group* | GO:0003677~DNA binding,GO:0003700~transcription factor activity, sequence-specific DNA binding, |
| 68 | 110429775 | bZIP transcription factor 68-like(LOC110429775) | *Sorghum bicolor* | GO:0003700~transcription factor activity, sequence-specific DNA binding,GO:0043565~sequence-specific DNA binding, |
| 69 | 841670 | beta glucosidase 18(BGLU18) | *Arabidopsis thaliana* | GO:0004553~hydrolase activity, hydrolyzing O-glycosyl compounds,GO:0008422~beta-glucosidase activity,GO:0042802~identical protein binding,GO:0051993~abscisic acid glucose ester beta-glucosidase activity, |
| 70 | 4329854 | beta-1,4-mannosyl-glycoprotein 4-beta-N-acetylglucosaminyltransferase(LOC4329854) | *Oryza sativa Japonica Group* | GO:0003830~beta-1,4-mannosylglycoprotein 4-beta-N-acetylglucosaminyltransferase activity, |
| 71 | 4346248 | beta-galactosidase 11-like(LOC4346248) | *Oryza sativa Japonica Group* | GO:0004565~beta-galactosidase activity,GO:0030246~carbohydrate binding, |
| 72 | 8061631 | calcium-binding protein KIC(LOC8061631) | *Sorghum bicolor* | GO:0005509~calcium ion binding, |
| 73 | 840471 | calcium-dependent protein kinase 2(CDPK2) | *Arabidopsis thaliana* | GO:0004672~protein kinase activity,GO:0004674~protein serine/threonine kinase activity,GO:0004683~calmodulin-dependent protein kinase activity,GO:0005509~calcium ion binding,GO:0005515~protein binding,GO:0005516~calmodulin binding,GO:0005524~ATP binding,GO:0009931~calcium-dependent protein serine/threonine kinase activity,GO:0016301~kinase activity, |
| 74 | 825807 | calcium-dependent protein kinase 21(CPK21) | *Arabidopsis thaliana* | GO:0004672~protein kinase activity,GO:0004683~calmodulin-dependent protein kinase activity,GO:0005509~calcium ion binding,GO:0005515~protein binding,GO:0005516~calmodulin binding,GO:0005524~ATP binding,GO:0009931~calcium-dependent protein serine/threonine kinase activity,GO:0019903~protein phosphatase binding, |
| 75 | 4346187 | calcium-dependent protein kinase 21-like(LOC4346187) | *Oryza sativa Japonica Group* | GO:0004683~calmodulin-dependent protein kinase activity,GO:0005509~calcium ion binding,GO:0005516~calmodulin binding,GO:0005524~ATP binding,GO:0009931~calcium-dependent protein serine/threonine kinase activity, |
| 76 | 825809 | calcium-dependent protein kinase 23(CPK23) | *Arabidopsis thaliana* | GO:0004672~protein kinase activity,GO:0004683~calmodulin-dependent protein kinase activity,GO:0005509~calcium ion binding,GO:0005515~protein binding,GO:0005516~calmodulin binding,GO:0005524~ATP binding,GO:0009931~calcium-dependent protein serine/threonine kinase activity,GO:0019903~protein phosphatase binding, |
| 77 | 8083705 | calmodulin(LOC8083705) | *Sorghum bicolor* | GO:0005509~calcium ion binding, |
| 78 | 4352458 | condensin-2 complex subunit D3(LOC4352458) | *Oryza sativa Japonica Group* | GO:0003682~chromatin binding,GO:0042393~histone binding, |
| 79 | 839226 | cullin 3(CUL3) | *Arabidopsis thaliana* | GO:0004842~ubiquitin-protein transferase activity,GO:0005515~protein binding,GO:0031625~ubiquitin protein ligase binding, |
| 80 | 8082122 | cyclic dof factor 1(LOC8082122) | *Sorghum bicolor* | GO:0003677~DNA binding,GO:0003700~transcription factor activity, sequence-specific DNA binding, |
| 81 | 8078579 | cyclic dof factor 2(LOC8078579) | *Sorghum bicolor* | GO:0003677~DNA binding,GO:0003700~transcription factor activity, sequence-specific DNA binding, |
| 82 | 827663 | cytochrome P450, family 707, subfamily A, polypeptide 1(CYP707A1) | *Arabidopsis thaliana* | GO:0004497~monooxygenase activity,GO:0005506~iron ion binding,GO:0010295~(+)-abscisic acid 8'-hydroxylase activity,GO:0016491~oxidoreductase activity,GO:0016705~oxidoreductase activity, acting on paired donors, with incorporation or reduction of molecular oxygen,GO:0016709~oxidoreductase activity, acting on paired donors, with incorporation or reduction of molecular oxygen, NAD(P)H as one donor, and incorporation of one atom of oxygen,GO:0020037~heme binding, |
| 83 | 834570 | cytochrome P450, family 707, subfamily A, polypeptide 3(CYP707A3) | *Arabidopsis thaliana* | GO:0004497~monooxygenase activity,GO:0005506~iron ion binding,GO:0010295~(+)-abscisic acid 8'-hydroxylase activity,GO:0016491~oxidoreductase activity,GO:0016709~oxidoreductase activity, acting on paired donors, with incorporation or reduction of molecular oxygen, NAD(P)H as one donor, and incorporation of one atom of oxygen,GO:0020037~heme binding, |
| 84 | 4347620 | dehydration-responsive element-binding protein 1A-like(LOC4347620) | *Oryza sativa Japonica Group* | GO:0003677~DNA binding,GO:0003700~transcription factor activity, sequence-specific DNA binding, |
| 85 | 4339974 | dehydration-responsive element-binding protein 1C(LOC4339974) | *Oryza sativa Japonica Group* | GO:0003677~DNA binding,GO:0003700~transcription factor activity, sequence-specific DNA binding, |
| 86 | 4324418 | dehydration-responsive element-binding protein 2A-like(LOC4324418) | *Oryza sativa Japonica Group* | GO:0000976~transcription regulatory region sequence-specific DNA binding,GO:0003700~transcription factor activity, sequence-specific DNA binding, |
| 87 | 4343863 | dehydrodolichyl diphosphate synthase 6(LOC4343863) | *Oryza sativa Japonica Group* | GO:0002094~polyprenyltransferase activity,GO:0016765~transferase activity, transferring alkyl or aryl (other than methyl) groups, |
| 88 | 8068630 | disease resistance protein RPS2(LOC8068630) | *Sorghum bicolor* | GO:0043531~ADP binding, |
| 89 | 4331811 | elongation factor 1-alpha-like(LOC4331811) | *Oryza sativa Japonica Group* | GO:0003746~translation elongation factor activity,GO:0003924~GTPase activity,GO:0005525~GTP binding, |
| 90 | 8082391 | ethylene-responsive transcription factor 4(LOC8082391) | *Sorghum bicolor* | GO:0000976~transcription regulatory region sequence-specific DNA binding,GO:0003700~transcription factor activity, sequence-specific DNA binding, |
| 91 | 4332538 | eukaryotic translation initiation factor 2 subunit alpha homolog(LOC4332538) | *Oryza sativa Japonica Group* | GO:0003743~translation initiation factor activity,GO:0043022~ribosome binding, |
| 92 | 821781 | far-red elongated hypocotyls 3(FHY3) | *Arabidopsis thaliana* | GO:0001228~transcriptional activator activity, RNA polymerase II transcription regulatory region sequence-specific binding,GO:0003700~transcription factor activity, sequence-specific DNA binding,GO:0005515~protein binding,GO:0008270~zinc ion binding, |
| 93 | 832311 | fatty acid reductase 1(FAR1) | *Arabidopsis thaliana* | GO:0050062~long-chain-fatty-acyl-CoA reductase activity,GO:0080019~fatty-acyl-CoA reductase (alcohol-forming) activity, |
| 94 | 819331 | galactinol synthase 1(GolS1) | *Arabidopsis thaliana* | GO:0016757~transferase activity, transferring glycosyl groups,GO:0046872~metal ion binding,GO:0047216~inositol 3-alpha-galactosyltransferase activity, |
| 95 | 842114 | galactinol synthase 2(GolS2) | *Arabidopsis thaliana* | GO:0016757~transferase activity, transferring glycosyl groups,GO:0046872~metal ion binding,GO:0047216~inositol 3-alpha-galactosyltransferase activity, |
| 96 | 4324110 | gallate 1-beta-glucosyltransferase(LOC4324110) | *Oryza sativa Japonica Group* | GO:0008194~UDP-glycosyltransferase activity,GO:0080043~quercetin 3-O-glucosyltransferase activity,GO:0080044~quercetin 7-O-glucosyltransferase activity, |
| 97 | 100272932 | germin-like protein 2(LOC100272932) | *Zea mays* | GO:0030145~manganese ion binding, |
| 98 | 4344999 | germin-like protein 8-12(LOC4344999) | *Oryza sativa Japonica Group* | GO:0030145~manganese ion binding, |
| 99 | 4347311 | glucose-6-phosphate isomerase 1, chloroplastic(LOC4347311) | *Oryza sativa Japonica Group* | GO:0004347~glucose-6-phosphate isomerase activity, |
| 100 | 818936 | glutathione peroxidase 3(GPX3) | *Arabidopsis thaliana* | GO:0004601~peroxidase activity,GO:0004602~glutathione peroxidase activity, |
| 101 | 4336181 | glycosyl hydrolase 5 family protein(LOC4336181) | *Oryza sativa Japonica Group* | GO:0016985~mannan endo-1,4-beta-mannosidase activity, |
| 102 | 8057776 | heat shock 70 kDa protein(LOC8057776) | *Sorghum bicolor* | GO:0005524~ATP binding,GO:0031072~heat shock protein binding,GO:0044183~protein binding involved in protein folding,GO:0051082~unfolded protein binding,GO:0051787~misfolded protein binding, |
| 103 | 8055854 | heat shock protein 81-2(LOC8055854) | *Sorghum bicolor* | GO:0005524~ATP binding,GO:0051082~unfolded protein binding, |
| 104 | 103641629 | histone H3.2(LOC103641629) | *Zea mays* | GO:0003677~DNA binding,GO:0046982~protein heterodimerization activity, |
| 105 | 829969 | histone deacetylase 1(HD1) | *Arabidopsis thaliana* | GO:0004407~histone deacetylase activity,GO:0005515~protein binding,GO:0008270~zinc ion binding,GO:0032041~NAD-dependent histone deacetylase activity (H3-K14 specific),GO:0046872~metal ion binding, |
| 106 | 836431 | histone deacetylase 6(HDA6) | *Arabidopsis thaliana* | GO:0004407~histone deacetylase activity,GO:0005515~protein binding,GO:0008270~zinc ion binding,GO:0032041~NAD-dependent histone deacetylase activity (H3-K14 specific),GO:0046872~metal ion binding, |
| 107 | 843671 | homeodomain GLABROUS 11(HDG11) | *Arabidopsis thaliana* | GO:0000976~transcription regulatory region sequence-specific DNA binding,GO:0000981~RNA polymerase II transcription factor activity, sequence-specific DNA binding,GO:0003677~DNA binding,GO:0003700~transcription factor activity, sequence-specific DNA binding,GO:0005515~protein binding,GO:0008289~lipid binding, |
| 108 | 8061169 | light-inducible protein CPRF2(LOC8061169) | *Sorghum bicolor* | GO:0003700~transcription factor activity, sequence-specific DNA binding, |
| 109 | 103635265 | methylesterase 7(LOC103635265) | *Zea mays* | GO:0080030~methyl indole-3-acetate esterase activity,GO:0080031~methyl salicylate esterase activity,GO:0080032~methyl jasmonate esterase activity, |
| 110 | 4327316 | monothiol glutaredoxin-S2-like(LOC4327316) | *Oryza sativa Japonica Group* | GO:0046872~metal ion binding,GO:0051537~2 iron, 2 sulfur cluster binding, |
| 111 | 837403 | myb domain protein 60(MYB60) | *Arabidopsis thaliana* | GO:0000976~transcription regulatory region sequence-specific DNA binding,GO:0003700~transcription factor activity, sequence-specific DNA binding, |
| 112 | 836865 | myb domain protein r1(MYBR1) | *Arabidopsis thaliana* | GO:0000976~transcription regulatory region sequence-specific DNA binding,GO:0000978~RNA polymerase II core promoter proximal region sequence-specific DNA binding,GO:0000981~RNA polymerase II transcription factor activity, sequence-specific DNA binding,GO:0003700~transcription factor activity, sequence-specific DNA binding,GO:0005515~protein binding,GO:0042802~identical protein binding,GO:0043565~sequence-specific DNA binding, |
| 113 | 820667 | nine-cis-epoxycarotenoid dioxygenase 3(NCED3) | *Arabidopsis thaliana* | GO:0010436~carotenoid dioxygenase activity,GO:0045549~9-cis-epoxycarotenoid dioxygenase activity,GO:0046872~metal ion binding, |
| 114 | 4331362 | non-specific lipid-transfer protein 2-like(LOC4331362) | *Oryza sativa Japonica Group* | GO:0008289~lipid binding, |
| 115 | 4326871 | ocs element-binding factor 1(LOC4326871) | *Oryza sativa Japonica Group* | GO:0000976~transcription regulatory region sequence-specific DNA binding,GO:0003700~transcription factor activity, sequence-specific DNA binding, |
| 116 | 817876 | peroxisomal 3-ketoacyl-CoA thiolase 3(PKT3) | *Arabidopsis thaliana* | GO:0003988~acetyl-CoA C-acyltransferase activity,GO:0005515~protein binding,GO:0016747~transferase activity, transferring acyl groups other than amino-acyl groups, |
| 117 | 825316 | plasma membrane intrinsic protein 1A(PIP1A) | *Arabidopsis thaliana* | GO:0015250~water channel activity,GO:0015267~channel activity, |
| 118 | 824510 | plasma membrane intrinsic protein 2A(PIP2A) | *Arabidopsis thaliana* | GO:0003729~mRNA binding,GO:0015250~water channel activity,GO:0015267~channel activity,GO:0031625~ubiquitin protein ligase binding, |
| 119 | 8057594 | polyphenol oxidase I, chloroplastic(LOC8057594) | *Sorghum bicolor* | GO:0004097~catechol oxidase activity,GO:0046872~metal ion binding, |
| 120 | 827555 | potassium channel KAT1-like protein(KAT2) | *Arabidopsis thaliana* | GO:0005242~inward rectifier potassium channel activity,GO:0005249~voltage-gated potassium channel activity,GO:0005515~protein binding, |
| 121 | 100281081 | potassium transporter 10(LOC100281081) | *Zea mays* | GO:0015079~potassium ion transmembrane transporter activity, |
| 122 | 4342173 | potassium transporter 22-like(LOC4342173) | *Oryza sativa Japonica Group* | GO:0015079~potassium ion transmembrane transporter activity, |
| 123 | 8061071 | probable ADP,ATP carrier protein At5g56450(LOC8061071) | *Sorghum bicolor* | GO:0005471~ATP:ADP antiporter activity, |
| 124 | 4349916 | probable alkaline/neutral invertase F(LOC4349916) | *Oryza sativa Japonica Group* | GO:0004575~sucrose alpha-glucosidase activity,GO:0033926~glycopeptide alpha-N-acetylgalactosaminidase activity, |
| 125 | 8082723 | probable alpha,alpha-trehalose-phosphate synthase [UDP-forming] 11(LOC8082723) | *Sorghum bicolor* | GO:0003824~catalytic activity, |
| 126 | 4330049 | probable aquaporin PIP2-2(LOC4330049) | *Oryza sativa Japonica Group* | GO:0015250~water channel activity, |
| 127 | 4332731 | probable calcium-binding protein CML27(LOC4332731) | *Oryza sativa Japonica Group* | GO:0005509~calcium ion binding, |
| 128 | 4342464 | probable carboxylesterase 15(LOC4342464) | *Oryza sativa Japonica Group* | GO:0016787~hydrolase activity, |
| 129 | 4338289 | probable glycerol-3-phosphate acyltransferase 3(LOC4338289) | *Oryza sativa Japonica Group* | GO:0016746~transferase activity, transferring acyl groups,GO:0016791~phosphatase activity,GO:0090447~glycerol-3-phosphate 2-O-acyltransferase activity, |
| 130 | 4337721 | probable inactive DNA (cytosine-5)-methyltransferase DRM3(LOC4337721) | *Oryza sativa Japonica Group* | GO:0003677~DNA binding,GO:0008168~methyltransferase activity, |
| 131 | 8085105 | probable manganese-transporting ATPase PDR2(LOC8085105) | *Sorghum bicolor* | GO:0005524~ATP binding,GO:0019829~cation-transporting ATPase activity, |
| 132 | 4337170 | probable pectinesterase/pectinesterase inhibitor 13(LOC4337170) | *Oryza sativa Japonica Group* | GO:0004857~enzyme inhibitor activity,GO:0030599~pectinesterase activity,GO:0045330~aspartyl esterase activity, |
| 133 | 8078643 | probable protein phosphatase 2C 6(LOC8078643) | *Sorghum bicolor* | GO:0046872~metal ion binding, |
| 134 | 4339571 | probable purine permease 4(LOC4339571) | *Oryza sativa Japonica Group* | GO:0005345~purine nucleobase transmembrane transporter activity,GO:0015211~purine nucleoside transmembrane transporter activity, |
| 135 | 4333501 | protein DETOXIFICATION 29(LOC4333501) | *Oryza sativa Japonica Group* | GO:0015297~antiporter activity,GO:0022857~transmembrane transporter activity,GO:0042910~xenobiotic transporter activity, |
| 136 | 4342431 | protein ETHYLENE-INSENSITIVE 2-like(LOC4342431) | *Oryza sativa Japonica Group* | GO:0005384~manganese ion transmembrane transporter activity,GO:0015086~cadmium ion transmembrane transporter activity, |
| 137 | 4336249 | protein NRT1/ PTR FAMILY 4.5(LOC4336249) | *Oryza sativa Japonica Group* | GO:0022857~transmembrane transporter activity, |
| 138 | 4340300 | protein NUCLEAR FUSION DEFECTIVE 4(LOC4340300) | *Oryza sativa Japonica Group* | GO:0022857~transmembrane transporter activity, |
| 139 | 8072447 | protein PIN-LIKES 2(LOC8072447) | *Sorghum bicolor* | GO:0010329~auxin efflux transmembrane transporter activity, |
| 140 | 8064218 | putative linoleate 9S-lipoxygenase 3(LOC8064218) | *Sorghum bicolor* | GO:0016702~oxidoreductase activity, acting on single donors with incorporation of molecular oxygen, incorporation of two atoms of oxygen,GO:0046872~metal ion binding, |
| 141 | 110432163 | putative receptor-like protein kinase At4g00960(LOC110432163) | *Sorghum bicolor* | GO:0004674~protein serine/threonine kinase activity,GO:0005524~ATP binding, |
| 142 | 843258 | receptor-like protein kinase 1(RPK1) | *Arabidopsis thaliana* | GO:0004672~protein kinase activity,GO:0004674~protein serine/threonine kinase activity,GO:0005515~protein binding,GO:0005524~ATP binding,GO:0016301~kinase activity,GO:0042802~identical protein binding, |
| 143 | 8062168 | receptor-like protein kinase HSL1(LOC8062168) | *Sorghum bicolor* | GO:0004674~protein serine/threonine kinase activity,GO:0005524~ATP binding, |
| 144 | 838452 | regulatory component of ABA receptor 1(RCAR1) | *Arabidopsis thaliana* | GO:0004864~protein phosphatase inhibitor activity,GO:0005515~protein binding,GO:0010427~abscisic acid binding,GO:0038023~signaling receptor activity,GO:0042803~protein homodimerization activity, |
| 145 | 834842 | respiratory burst oxidase homologue D(RBOHD) | *Arabidopsis thaliana* | GO:0004601~peroxidase activity,GO:0005509~calcium ion binding,GO:0005515~protein binding,GO:0016174~NAD(P)H oxidase activity, |
| 146 | 842710 | respiratory burst oxidase protein F(RBOH F) | *Arabidopsis thaliana* | GO:0004601~peroxidase activity,GO:0005509~calcium ion binding,GO:0005515~protein binding,GO:0016174~NAD(P)H oxidase activity,GO:0046872~metal ion binding,GO:0050664~oxidoreductase activity, acting on NAD(P)H, oxygen as acceptor, |
| 147 | 100381320 | ripening-related protein(LOC100381320) | *Zea mays* | GO:0004857~enzyme inhibitor activity, |
| 148 | 8055864 | tonoplast dicarboxylate transporter(LOC8055864) | *Sorghum bicolor* | GO:0022857~transmembrane transporter activity, |
| 149 | 8075588 | transcription factor VOZ1(LOC8075588) | *Sorghum bicolor* | GO:0043565~sequence-specific DNA binding, |
| 150 | 4328666 | transcription initiation factor TFIID subunit 15b(LOC4328666) | *Oryza sativa Japonica Group* | GO:0046872~metal ion binding, |
| 151 | 844194 | trehalose-6-phosphate synthase(TPS1) | *Arabidopsis thaliana* | GO:0003825~alpha,alpha-trehalose-phosphate synthase (UDP-forming) activity,GO:0016757~transferase activity, transferring glycosyl groups, |
| 152 | 819323 | ubiquitin 6(UBQ6) | *Arabidopsis thaliana* | GO:0003729~mRNA binding,GO:0003735~structural constituent of ribosome,GO:0031386~protein tag,GO:0031625~ubiquitin protein ligase binding,GO:0046872~metal ion binding, |
| 153 | 100217031 | uncharacterized LOC100217031(LOC100217031) | *Zea mays* | GO:0016301~kinase activity,GO:0016787~hydrolase activity, |
| 154 | 100275707 | uncharacterized LOC100275707(LOC100275707) | *Zea mays* | GO:0004519~endonuclease activity, |
| 155 | 100279496 | uncharacterized LOC100279496(LOC100279496) | *Zea mays* | GO:0046556~alpha-L-arabinofuranosidase activity, |
| 156 | 100283330 | uncharacterized LOC100283330(LOC100283330) | *Zea mays* | GO:0042626~ATPase activity, coupled to transmembrane movement of substances,GO:0046961~proton-transporting ATPase activity, rotational mechanism, |
| 157 | 100283536 | uncharacterized LOC100283536(LOC100283536) | *Zea mays* | GO:0016410~N-acyltransferase activity,GO:0016747~transferase activity, transferring acyl groups other than amino-acyl groups, |
| 158 | 4347825 | uncharacterized protein At2g39795, mitochondrial(LOC4347825) | *Oryza sativa Japonica Group* | GO:0003729~mRNA binding,GO:0008494~translation activator activity,GO:0097177~mitochondrial ribosome binding, |
| 159 | 4341520 | zinc finger A20 and AN1 domain-containing stress-associated protein 8(LOC4341520) | *Oryza sativa Japonica Group* | GO:0003677~DNA binding,GO:0008270~zinc ion binding, |
| 160 | 4350003 | zinc finger CCHC domain-containing protein 10(LOC4350003) | *Oryza sativa Japonica Group* | GO:0003676~nucleic acid binding,GO:0008270~zinc ion binding, |

| **Supplementary Table 5**.130 genes with Gene ontology term for biological process | | | |
| --- | --- | --- | --- |
| ID | Gene Name | Species | GOTERM_BP_DIRECT |
| 826730 | 1-aminocyclopropane-1-carboxylic acid (acc) synthase 6(ACS6) | *Arabidopsis thaliana* | defense response,response to oxidative stress,biosynthetic process, response to water deprivation,response to wounding, response to mechanical stimulus,ethylene biosynthetic process, response to ethylene,response to auxin,response to jasmonic acid,fruit ripening,phloem or xylem histogenesiscell division |
| 8073342 | 16.9 kDa class I heat shock protein 1(LOC8073342) | *Sorghum bicolor* | protein folding,response to heat,response to salt stress,response to hydrogen peroxide,protein oligomerization, |
| 8058459 | 4-hydroxy-tetrahydrodipicolinate synthase, chloroplastic(LOC8058459) | *Sorghum bicolor* | lysine biosynthetic process via diaminopimelate,diaminopimelate biosynthetic process, |
| 4337526 | 50S ribosomal protein L28, chloroplastic(LOC4337526) | *Oryza sativa Japonica Group* | translation, |
| 8062999 | 60S ribosomal protein L10(LOC8062999) | *Sorghum bicolor* | ribosomal large subunit assembly,translation, |
| 103642077 | 60S ribosomal protein L27-3(LOC103642077) | *Zea mays* | translation, |
| 100193672 | 60S ribosomal protein L35a-like(LOC100193672) | *Zea mays* | cytoplasmic translation,ribosomal large subunit biogenesis, |
| 100125659 | ABC transporter C family MRP4(LOC100125659) | *Zea mays* | transmembrane transport, |
| 830541 | ABC-2 type transporter family protein(ABCG22) | *Arabidopsis thaliana* | response to water deprivation,transpiration, |
| 8068578 | AP3-complex subunit beta-A(LOC8068578) | *Sorghum bicolor* | intracellular protein transport,Golgi to vacuole transport,GO:0016192~vesicle-mediated transport,regulation of intracellular pH,lytic vacuole organization, |
| 843527 | ATP-binding casette family G25(ABCG25) | *Arabidopsis thaliana* | response to heat,response to cold,response to abscisic acid,abscisic acid-activated signaling pathway,intercellular transport,negative regulation of post-embryonic development,transmembrane transport,abscisic acid transport, |
| 840158 | Basic helix-loop-helix (bHLH) DNA-binding family protein(MYC2) | *Arabidopsis thaliana* | regulation of transcription, tryptophan metabolic process,response to desiccation,response to wounding,response to abscisic acid,abscisic acid-activated signaling pathway,response to jasmonic acid,indole glucosinolate biosynthetic process,jasmonic acid mediated signaling pathway,positive regulation of flavonoid biosynthetic process,response to chitin,stomatal complex development,regulation of transcription from RNA polymerase II promoter in response to oxidative stress,positive regulation of transcription, DNA-templated,regulation of sequence-specific DNA binding transcription factor activity,protein homotetramerization,regulation of tryptophan metabolic process,regulation of defense response to insect,regulation of secondary cell wall biogenesis, |
| 828653 | C-repeat/DRE binding factor 1(CBF1) | *Arabidopsis thaliana* | response to cold,response to water deprivation,Gcold acclimation,positive regulation of transcription, DNA-templated, |
| 828651 | C-repeat/DRE binding factor 2(CBF2) | *Arabidopsis thaliana* | regulation of transcription, DNA-templated,response to cold,cold acclimation, |
| 542390 | CAAT-box DNA binding protein subunit B (NF-YB)(LOC542390) | *Zea mays* | regulation of transcription from RNA polymerase II promoter, |
| 834443 | CAP-binding protein 20(CBP20) | *Arabidopsis thaliana* | RNA splicing, via endonucleolytic cleavage and ligation,mRNA splicing, via spliceosome,RNA metabolic process,primary miRNA processing,mRNA cis splicing, via spliceosome,defense response to virus, |
| 827604 | CBL-interacting protein kinase 12(CIPK12) | *Arabidopsis thaliana* | signal transduction, |
| 830556 | CBL-interacting protein kinase 15(CIPK15) | *Arabidopsis thaliana* | protein phosphorylation,signal transduction,abscisic acid-activated signaling pathway,negative regulation of abscisic acid-activated signaling pathway, |
| 8067611 | CBL-interacting protein kinase 15(LOC8067611) | *Sorghum bicolor* | signal transduction, |
| 9639733 | CBL-interacting protein kinase 23(LOC9639733) | *Selaginella moellendorffii* | signal transduction, |
| 8620286 | CLC 6/7 family protein(clcC) | *Dictyostelium discoideum AX4* | ion transport,chloride transport,ion transmembrane transport,regulation of ion transmembrane transport,transmembrane transport,chloride transmembrane transport, |
| 831241 | Chalcone and stilbene synthase family protein(TT4) | *Arabidopsis thaliana* | response to oxidative stress,biosynthetic process,response to wounding,response to gravity,chalcone biosynthetic process,response to auxin,response to jasmonic acid,flavonoid biosynthetic process,auxin polar transport,response to UV-B,polyketide biosynthetic process,~regulation of anthocyanin biosynthetic process, |
| 835180 | Clp ATPase(ERD1) | *Arabidopsis thaliana* | macromolecular complex assembly, |
| 8066485 | DExH-box ATP-dependent RNA helicase DExH12(LOC8066485) | *Sorghum bicolor* | RNA catabolic process, |
| 830424 | DRE-binding protein 2A(DREB2A) | *Arabidopsis thaliana* | regulation of transcription, DNA-templated,response to heat,response to water deprivation,response to UV-B,Gheat acclimation,response to hydrogen peroxide,positive regulation of transcription, DNA-templated,cellular response to hypoxia, |
| 4344172 | E3 ubiquitin-protein ligase DIS1-like(LOC4344172) | *Oryza sativa Japonica Group* | ubiquitin-dependent protein catabolic process,protein ubiquitination, |
| 110430822 | E3 ubiquitin-protein ligase RZF1-like(LOC110430822) | *Sorghum bicolor* | ubiquitin-dependent protein catabolic process, |
| 8083691 | ETHYLENE INSENSITIVE 3-like 1 protein(LOC8083691) | *Sorghum bicolor* | ethylene-activated signaling pathway, |
| 842213 | FAD/NAD(P)-binding oxidoreductase family protein(XF1) | *Arabidopsis thaliana* | response to water deprivation,response to ethylene,sterol biosynthetic process, |
| 827173 | FRS (FAR1 Related Sequences) transcription factor family(FAR1) | *Arabidopsis thaliana* | response to red or far red light,red or far-red light signaling pathway,far-red light signaling pathway,response to far red light,positive regulation of circadian rhythm,Gpositive regulation of transcription, DNA-templated,negative regulation of leaf senescence, |
| 4335831 | G-type lectin S-receptor-like serine/threonine-protein kinase At2g19130(LOC4335831) | *Oryza sativa Japonica Group* | recognition of pollen, |
| 837576 | Glutathione S-transferase family protein(ERD9) | *Arabidopsis thaliana* | glutathione metabolic process,toxin catabolic process,response to salt stress,de-etiolation,regulation of growthlateral root development,response to growth hormone,negative regulation of response to water deprivation,response to karrikin, |
| 816413 | H[+]-ATPase 1(HA1) | *Arabidopsis thaliana* | response to water deprivation,response to abscisic acid,regulation of stomatal movement,regulation of intracellular pH,hydrogen ion transmembrane transport,stomatal opening, |
| 831213 | IKI3 family protein(ABO1) | *Arabidopsis thaliana* | tRNA wobble base 5-methoxycarbonylmethyl-2-thiouridine biosynthesis.,tRNA modification,response to oxidative stress,positive regulation of cell proliferation,~auxin-activated signaling pathway,response to abscisic acid,abscisic acid-activated signaling pathway,regulation of abscisic acid-activated signaling pathway,~leaf morphogenesis,regulation of auxin mediated signaling pathway,Gnegative regulation of anthocyanin metabolic process,organ growth,fruit morphogenesis,GO:0051301~cell division,GO:0071215~cellular response to abscisic acid stimulus,GO:0080178~5-carbamoylmethyl uridine residue modification,GO:2000024~regulation of leaf development, |
| 838138 | Inorganic H pyrophosphatase family protein(AVP1) | *Arabidopsis thaliana* | GO:0005985~sucrose metabolic process,GO:0009414~response to water deprivation,GO:0009651~response to salt stress,GO:0009926~auxin polar transport,GO:0010248~establishment or maintenance of transmembrane electrochemical gradient,GO:0048366~leaf development,GO:0052546~cell wall pectin metabolic process,GO:1902600~hydrogen ion transmembrane transport,GO:2000904~regulation of starch metabolic process, |
| 8059924 | L-type lectin-domain containing receptor kinase IV.1(LOC8059924) | *Sorghum bicolor* | GO:0002229~defense response to oomycetes,GO:0042742~defense response to bacterium, |
| 4342446 | MD-2-related lipid-recognition protein ROSY1(LOC4342446) | *Oryza sativa Japonica Group* | GO:0015918~sterol transport,GO:0032366~intracellular sterol transport, |
| 4334553 | NAC domain-containing protein 2-like(LOC4334553) | *Oryza sativa Japonica Group* | GO:0006355~regulation of transcription, DNA-templated,GO:1901002~positive regulation of response to salt stress,GO:1902584~positive regulation of response to water deprivation, |
| 4325828 | NAC domain-containing protein 48-like(LOC4325828) | *Oryza sativa Japonica Group* | GO:0006355~regulation of transcription, DNA-templated, |
| 4326769 | NADP-dependent malic enzyme, chloroplastic-like(LOC4326769) | *Oryza sativa Japonica Group* | GO:0006090~pyruvate metabolic process,GO:0006108~malate metabolic process, |
| 831889 | NRAMP metal ion transporter family protein(EIN2) | *Arabidopsis thaliana* | GO:0001736~establishment of planar polarity,GO:0002237~response to molecule of bacterial origin,GO:0006970~response to osmotic stress,GO:0006979~response to oxidative stress,GO:0008219~cell death,GO:0009408~response to heat,GO:0009651~response to salt stress,GO:0009723~response to ethylene,GO:0009725~response to hormone,GO:0009734~auxin-activated signaling pathway,GO:0009736~cytokinin-activated signaling pathway,GO:0009753~response to jasmonic acid,GO:0009789~positive regulation of abscisic acid-activated signaling pathway,GO:0009871~jasmonic acid and ethylene-dependent systemic resistance, ethylene mediated signaling pathway,GO:0009873~ethylene-activated signaling pathway,GO:0009926~auxin polar transport,GO:0010087~phloem or xylem histogenesis,GO:0010119~regulation of stomatal movement,GO:0010150~leaf senescence,GO:0010182~sugar mediated signaling pathway,GO:0016573~histone acetylation,GO:0031348~negative regulation of defense response,GO:0042742~defense response to bacterium,GO:0043972~histone H3-K23 acetylation,GO:0044154~histone H3-K14 acetylation,GO:0048765~root hair cell differentiation,GO:0050832~defense response to fungus,GO:0051301~cell division,GO:0052544~defense response by callose deposition in cell wall, |
| 100192929 | Ninja-family protein 1(LOC100192929) | *Zea mays* | GO:0007165~signal transduction,GO:0045892~negative regulation of transcription, DNA-templated, |
| 841514 | Nucleolar GTP-binding protein(AT1G50920) | *Arabidopsis thaliana* | GO:0042254~ribosome biogenesis, |
| 820292 | Paxneb protein-like protein(ELO1) | *Arabidopsis thaliana* | GO:0002098~tRNA wobble uridine modification,GO:0006979~response to oxidative stress,GO:0008284~positive regulation of cell proliferation,GO:0009734~auxin-activated signaling pathway,GO:0009737~response to abscisic acid,GO:0009738~abscisic acid-activated signaling pathway,GO:0010928~regulation of auxin mediated signaling pathway,GO:0031538~negative regulation of anthocyanin metabolic process,GO:0035265~organ growth,GO:0043609~regulation of carbon utilization,GO:0051301~cell division,GO:0071329~cellular response to sucrose stimulus,GO:2000024~regulation of leaf development, |
| 829541 | Protein kinase superfamily protein(OST1) | *Arabidopsis thaliana* | GO:0005985~sucrose metabolic process,GO:0006468~protein phosphorylation,GO:0006636~unsaturated fatty acid biosynthetic process,GO:0006970~response to osmotic stress,GO:0009414~response to water deprivation,GO:0009651~response to salt stress,GO:0009737~response to abscisic acid,GO:0009738~abscisic acid-activated signaling pathway,GO:0009789~positive regulation of abscisic acid-activated signaling pathway,GO:0010118~stomatal movement,GO:0010119~regulation of stomatal movement,GO:0010359~regulation of anion channel activity,GO:0019432~triglyceride biosynthetic process,GO:0035556~intracellular signal transduction,GO:0042742~defense response to bacterium,GO:0046777~protein autophosphorylation,GO:0048366~leaf development,GO:0071244~cellular response to carbon dioxide,GO:0071485~cellular response to absence of light,GO:0090333~regulation of stomatal closure,GO:1902456~regulation of stomatal opening,GO:2000377~regulation of reactive oxygen species metabolic process, |
| 844164 | Protein kinase superfamily protein(SNRK2-8) | *Arabidopsis thaliana* | GO:0006468~protein phosphorylation,GO:0006970~response to osmotic stress,GO:0035556~intracellular signal transduction,GO:0080167~response to karrikin, |
| 828714 | Protein phosphatase 2C family protein(ABI1) | *Arabidopsis thaliana* | GO:0006470~protein dephosphorylation,GO:0009408~response to heat,GO:0009409~response to cold,GO:0009737~response to abscisic acid,GO:0009738~abscisic acid-activated signaling pathway,GO:0009787~regulation of abscisic acid-activated signaling pathway,GO:0009788~negative regulation of abscisic acid-activated signaling pathway,GO:0010119~regulation of stomatal movement, |
| 835809 | Protein phosphatase 2C family protein(ABI2) | *Arabidopsis thaliana* | GO:0006469~negative regulation of protein kinase activity,GO:0006470~protein dephosphorylation,GO:0006970~response to osmotic stress,GO:0009408~response to heat,GO:0009414~response to water deprivation,GO:0009737~response to abscisic acid,GO:0009738~abscisic acid-activated signaling pathway,GO:0009788~negative regulation of abscisic acid-activated signaling pathway,GO:0010205~photoinhibition,GO:1902456~regulation of stomatal opening, |
| 4340585 | RING-H2 finger protein ATL46(LOC4340585) | *Oryza sativa Japonica Group* | GO:0016567~protein ubiquitination, |
| 4344339 | RINT1-like protein MAG2L(LOC4344339) | *Oryza sativa Japonica Group* | GO:0006890~retrograde vesicle-mediated transport, Golgi to ER,GO:0060628~regulation of ER to Golgi vesicle-mediated transport, |
| 4332957 | RNA-binding protein CP29B, chloroplastic(LOC4332957) | *Oryza sativa Japonica Group* | GO:1901259~chloroplast rRNA processing, |
| 110431928 | TOM1-like protein 2(LOC110431928) | *Sorghum bicolor* | GO:0043328~protein targeting to vacuole involved in ubiquitin-dependent protein catabolic process via the multivesicular body sorting pathway, |
| 110436990 | UDP-glucose 6-dehydrogenase 5(LOC110436990) | *Sorghum bicolor* | GO:0000271~polysaccharide biosynthetic process,GO:0006024~glycosaminoglycan biosynthetic process,GO:0006065~UDP-glucuronate biosynthetic process, |
| 835726 | WRKY DNA-binding protein 2(WRKY2) | *Arabidopsis thaliana* | GO:0006355~regulation of transcription, DNA-templated,GO:0009555~pollen development,GO:0009942~longitudinal axis specification,GO:0030010~establishment of cell polarity, |
| 4324824 | WUSCHEL-related homeobox 9-like(LOC4324824) | *Oryza sativa Japonica Group* | GO:0099402~plant organ development, |
| 817257 | abscisic aldehyde oxidase 3(AAO3) | *Arabidopsis thaliana* | GO:0009688~abscisic acid biosynthetic process,GO:0009851~auxin biosynthetic process, |
| 820121 | ascorbate peroxidase 2(APX2) | *Arabidopsis thaliana* | GO:0000302~response to reactive oxygen species,GO:0006979~response to oxidative stress,GO:0034599~cellular response to oxidative stress,GO:0042744~hydrogen peroxide catabolic process, |
| 8067406 | auxin response factor 22(LOC8067406) | *Sorghum bicolor* | GO:0006355~regulation of transcription, DNA-templated,GO:0009734~auxin-activated signaling pathway, |
| 4349090 | auxin-responsive protein SAUR32(LOC4349090) | *Oryza sativa Japonica Group* | GO:0009733~response to auxin, |
| 4330838 | bZIP transcription factor 23-like(LOC4330838) | *Oryza sativa Japonica Group* | GO:0009414~response to water deprivation,GO:0009651~response to salt stress,GO:0009738~abscisic acid-activated signaling pathway,GO:0045893~positive regulation of transcription, DNA-templated, |
| 110429775 | bZIP transcription factor 68-like(LOC110429775) | *Sorghum bicolor* | GO:0006355~regulation of transcription, DNA-templated, |
| 841670 | beta glucosidase 18(BGLU18) | *Arabidopsis thaliana* | GO:0005975~carbohydrate metabolic process,GO:0009414~response to water deprivation,GO:0009625~response to insect,GO:0009651~response to salt stress,GO:0009687~abscisic acid metabolic process,GO:0009737~response to abscisic acid,GO:0009738~abscisic acid-activated signaling pathway,GO:0009789~positive regulation of abscisic acid-activated signaling pathway,GO:0010119~regulation of stomatal movement,GO:0019762~glucosinolate catabolic process,GO:0030104~water homeostasis,GO:0050832~defense response to fungus,GO:0051258~protein polymerization,GO:0080119~ER body organization, |
| 4329854 | beta-1,4-mannosyl-glycoprotein 4-beta-N-acetylglucosaminyltransferase(LOC4329854) | *Oryza sativa Japonica Group* | GO:0006487~protein N-linked glycosylation, |
| 4346248 | beta-galactosidase 11-like(LOC4346248) | *Oryza sativa Japonica Group* | GO:0005975~carbohydrate metabolic process, |
| 840471 | calcium-dependent protein kinase 2(CDPK2) | *Arabidopsis thaliana* | GO:0006468~protein phosphorylation,GO:0009789~positive regulation of abscisic acid-activated signaling pathway,GO:0018105~peptidyl-serine phosphorylation,GO:0035556~intracellular signal transduction,GO:0046777~protein autophosphorylation,GO:0080092~regulation of pollen tube growth,GO:1901979~regulation of inward rectifier potassium channel activity, |
| 825807 | calcium-dependent protein kinase 21(CPK21) | *Arabidopsis thaliana* | GO:0018105~peptidyl-serine phosphorylation,GO:0035556~intracellular signal transduction,GO:0046777~protein autophosphorylation, |
| 4346187 | calcium-dependent protein kinase 21-like(LOC4346187) | *Oryza sativa Japonica Group* | GO:0009737~response to abscisic acid,GO:0018105~peptidyl-serine phosphorylation,GO:0035556~intracellular signal transduction,GO:0046777~protein autophosphorylation,GO:1901001~negative regulation of response to salt stress, |
| 825809 | calcium-dependent protein kinase 23(CPK23) | *Arabidopsis thaliana* | GO:0006468~protein phosphorylation,GO:0009737~response to abscisic acid,GO:0010119~regulation of stomatal movement,GO:0018105~peptidyl-serine phosphorylation,GO:0035556~intracellular signal transduction,GO:0046686~response to cadmium ion,GO:0046777~protein autophosphorylation, |
| 4352458 | condensin-2 complex subunit D3(LOC4352458) | *Oryza sativa Japonica Group* | GO:0007076~mitotic chromosome condensation,GO:0009556~microsporogenesis,GO:0010032~meiotic chromosome condensation,GO:0098653~centromere clustering, |
| 8058923 | coronatine-insensitive protein homolog 1a(LOC8058923) | *Sorghum bicolor* | GO:0002213~defense response to insect,GO:0031146~SCF-dependent proteasomal ubiquitin-dependent protein catabolic process,GO:2000022~regulation of jasmonic acid mediated signaling pathway, |
| 839226 | cullin 3(CUL3) | *Arabidopsis thaliana* | GO:0006511~ubiquitin-dependent protein catabolic process,GO:0009639~response to red or far red light,GO:0009793~embryo development ending in seed dormancy,GO:0009911~positive regulation of flower development,GO:0009960~endosperm development,GO:0016567~protein ubiquitination, |
| 827663 | cytochrome P450, family 707, subfamily A, polypeptide 1(CYP707A1) | *Arabidopsis thaliana* | GO:0009639~response to red or far red light,GO:0009687~abscisic acid metabolic process,GO:0016125~sterol metabolic process,GO:0046345~abscisic acid catabolic process,GO:0048838~release of seed from dormancy,GO:0050832~defense response to fungus, |
| 834570 | cytochrome P450, family 707, subfamily A, polypeptide 3(CYP707A3) | *Arabidopsis thaliana* | GO:0009414~response to water deprivation,GO:0009639~response to red or far red light,GO:0009687~abscisic acid metabolic process,GO:0016125~sterol metabolic process,GO:0046345~abscisic acid catabolic process,GO:0071456~cellular response to hypoxia, |
| 4324418 | dehydration-responsive element-binding protein 2A-like(LOC4324418) | *Oryza sativa Japonica Group* | GO:0045893~positive regulation of transcription, DNA-templated, |
| 4343863 | dehydrodolichyl diphosphate synthase 6(LOC4343863) | *Oryza sativa Japonica Group* | GO:0016094~polyprenol biosynthetic process, |
| 8082391 | ethylene-responsive transcription factor 4(LOC8082391) | *Sorghum bicolor* | GO:0009409~response to cold,GO:0009414~response to water deprivation,GO:0009611~response to wounding,GO:0009625~response to insect,GO:0009651~response to salt stress,GO:0009723~response to ethylene,GO:0009733~response to auxin,GO:0009735~response to cytokinin,GO:0009737~response to abscisic acid,GO:0009751~response to salicylic acid,GO:0009753~response to jasmonic acid,GO:0010311~lateral root formation,GO:0010337~regulation of salicylic acid metabolic process,GO:0010364~regulation of ethylene biosynthetic process,GO:0010728~regulation of hydrogen peroxide biosynthetic process,GO:0045892~negative regulation of transcription, DNA-templated,GO:0080027~response to herbivore,GO:0080113~regulation of seed growth,GO:0080141~regulation of jasmonic acid biosynthetic process,GO:2000068~regulation of defense response to insect, |
| 103650094 | expansin-B11-like(LOC103650094) | *Zea mays* | GO:0019953~sexual reproduction, |
| 821781 | far-red elongated hypocotyls 3(FHY3) | *Arabidopsis thaliana* | GO:0006355~regulation of transcription, DNA-templated,GO:0007623~circadian rhythm,GO:0009585~red, far-red light phototransduction,GO:0010017~red or far-red light signaling pathway,GO:0010218~response to far red light,GO:0042753~positive regulation of circadian rhythm,GO:0045893~positive regulation of transcription, DNA-templated,GO:1900056~negative regulation of leaf senescence, |
| 4339617 | fasciclin-like arabinogalactan protein 13(LOC4339617) | *Oryza sativa Japonica Group* | GO:0009834~plant-type secondary cell wall biogenesis, |
| 832311 | fatty acid reductase 1(FAR1) | *Arabidopsis thaliana* | GO:0006629~lipid metabolic process,GO:0009611~response to wounding,GO:0009651~response to salt stress,GO:0010345~suberin biosynthetic process,GO:0035336~long-chain fatty-acyl-CoA metabolic process, |
| 819331 | galactinol synthase 1(GolS1) | *Arabidopsis thaliana* | GO:0006012~galactose metabolic process,GO:0006979~response to oxidative stress,GO:0009408~response to heat,GO:0009409~response to cold,GO:0009414~response to water deprivation,GO:0009651~response to salt stress,GO:0009737~response to abscisic acid, |
| 842114 | galactinol synthase 2(GolS2) | *Arabidopsis thaliana* | GO:0006012~galactose metabolic process,GO:0006979~response to oxidative stress,GO:0009409~response to cold,GO:0009414~response to water deprivation,GO:0009651~response to salt stress,GO:0009737~response to abscisic acid, |
| 4347311 | glucose-6-phosphate isomerase 1, chloroplastic(LOC4347311) | *Oryza sativa Japonica Group* | GO:0006094~gluconeogenesis,GO:0006096~glycolytic process, |
| 818936 | glutathione peroxidase 3(GPX3) | *Arabidopsis thaliana* | GO:0006979~response to oxidative stress,GO:0009738~abscisic acid-activated signaling pathway,GO:0042542~response to hydrogen peroxide,GO:0042631~cellular response to water deprivation, |
| 4336181 | glycosyl hydrolase 5 family protein(LOC4336181) | *Oryza sativa Japonica Group* | GO:0071704~organic substance metabolic process, |
| 8057776 | heat shock 70 kDa protein(LOC8057776) | *Sorghum bicolor* | GO:0034620~cellular response to unfolded protein,GO:0042026~protein refolding,GO:0051085~chaperone mediated protein folding requiring cofactor, |
| 8055854 | heat shock protein 81-2(LOC8055854) | *Sorghum bicolor* | GO:0006457~protein folding,GO:0034605~cellular response to heat,GO:0050821~protein stabilization, |
| 829969 | histone deacetylase 1(HD1) | *Arabidopsis thaliana* | GO:0009294~DNA mediated transformation,GO:0009861~jasmonic acid and ethylene-dependent systemic resistance,GO:0016573~histone acetylation,GO:0016575~histone deacetylation,GO:0042742~defense response to bacterium,GO:0045892~negative regulation of transcription, DNA-templated,GO:1901001~negative regulation of response to salt stress,GO:1902459~positive regulation of stem cell population maintenance,GO:2000026~regulation of multicellular organismal development, |
| 836431 | histone deacetylase 6(HDA6) | *Arabidopsis thaliana* | GO:0009651~response to salt stress,GO:0009737~response to abscisic acid,GO:0009793~embryo development ending in seed dormancy,GO:0010228~vegetative to reproductive phase transition of meristem,GO:0010431~seed maturation,GO:0016441~posttranscriptional gene silencing,GO:0016458~gene silencing,GO:0016575~histone deacetylation,GO:0048510~regulation of timing of transition from vegetative to reproductive phase, |
| 843671 | homeodomain GLABROUS 11(HDG11) | *Arabidopsis thaliana* | GO:0009828~plant-type cell wall loosening,GO:0010091~trichome branching, |
| 103635265 | methylesterase 7(LOC103635265) | *Zea mays* | GO:0009694~jasmonic acid metabolic process,GO:0009696~salicylic acid metabolic process, |
| 837403 | myb domain protein 60(MYB60) | *Arabidopsis thaliana* | GO:0009409~response to cold,GO:0009411~response to UV,GO:0009414~response to water deprivation,GO:0009416~response to light stimulus,GO:0009637~response to blue light,GO:0009646~response to absence of light,GO:0009733~response to auxin,GO:0009737~response to abscisic acid,GO:0009744~response to sucrose,GO:0010118~stomatal movement,GO:0080148~negative regulation of response to water deprivation,GO:1902074~response to salt, |
| 836865 | myb domain protein r1(MYBR1) | *Arabidopsis thaliana* | GO:0006355~regulation of transcription, DNA-templated,GO:0009408~response to heat,GO:0009414~response to water deprivation,GO:0009651~response to salt stress,GO:0009737~response to abscisic acid,GO:0009738~abscisic acid-activated signaling pathway,GO:0009751~response to salicylic acid,GO:0009753~response to jasmonic acid,GO:0010200~response to chitin,GO:0010929~positive regulation of auxin mediated signaling pathway,GO:0042742~defense response to bacterium,GO:0050832~defense response to fungus,GO:1900150~regulation of defense response to fungus,GO:2000022~regulation of jasmonic acid mediated signaling pathway,GO:2000031~regulation of salicylic acid mediated signaling pathway, |
| 820667 | nine-cis-epoxycarotenoid dioxygenase 3(NCED3) | *Arabidopsis thaliana* | GO:0006970~response to osmotic stress,GO:0009414~response to water deprivation,GO:0009688~abscisic acid biosynthetic process,GO:0016121~carotene catabolic process,GO:0042538~hyperosmotic salinity response, |
| 4331362 | non-specific lipid-transfer protein 2-like(LOC4331362) | *Oryza sativa Japonica Group* | GO:0006869~lipid transport, |
| 4326871 | ocs element-binding factor 1(LOC4326871) | *Oryza sativa Japonica Group* | GO:0045893~positive regulation of transcription, DNA-templated, |
| 817876 | peroxisomal 3-ketoacyl-CoA thiolase 3(PKT3) | *Arabidopsis thaliana* | GO:0006633~fatty acid biosynthetic process,GO:0006635~fatty acid beta-oxidation,GO:0009611~response to wounding,GO:0009695~jasmonic acid biosynthetic process,GO:0009789~positive regulation of abscisic acid-activated signaling pathway,GO:0010111~glyoxysome organization,GO:0010124~phenylacetate catabolic process,GO:0019395~fatty acid oxidation,GO:0031408~oxylipin biosynthetic process, |
| 825316 | plasma membrane intrinsic protein 1A(PIP1A) | *Arabidopsis thaliana* | GO:0006833~water transport,GO:0009414~response to water deprivation,GO:0055085~transmembrane transport, |
| 824510 | plasma membrane intrinsic protein 2A(PIP2A) | *Arabidopsis thaliana* | GO:0006833~water transport,GO:0009414~response to water deprivation,GO:0009737~response to abscisic acid,GO:0055085~transmembrane transport,GO:0080170~hydrogen peroxide transmembrane transport, |
| 8057594 | polyphenol oxidase I, chloroplastic(LOC8057594) | *Sorghum bicolor* | GO:0046148~pigment biosynthetic process, |
| 827555 | potassium channel KAT1-like protein(KAT2) | *Arabidopsis thaliana* | GO:0007623~circadian rhythm,GO:0009644~response to high light intensity,GO:0010118~stomatal movement,GO:0034765~regulation of ion transmembrane transport, |
| 100281081 | potassium transporter 10(LOC100281081) | *Zea mays* | GO:0006813~potassium ion transport, |
| 4342173 | potassium transporter 22-like(LOC4342173) | *Oryza sativa Japonica Group* | GO:0006813~potassium ion transport, |
| 8061071 | probable ADP,ATP carrier protein At5g56450(LOC8061071) | *Sorghum bicolor* | GO:0048653~anther development,GO:1990544~mitochondrial ATP transmembrane transport, |
| 4349916 | probable alkaline/neutral invertase F(LOC4349916) | *Oryza sativa Japonica Group* | GO:0005987~sucrose catabolic process, |
| 4338289 | probable glycerol-3-phosphate acyltransferase 3(LOC4338289) | *Oryza sativa Japonica Group* | GO:0010143~cutin biosynthetic process, |
| 4337721 | probable inactive DNA (cytosine-5)-methyltransferase DRM3(LOC4337721) | *Oryza sativa Japonica Group* | GO:0006306~DNA methylation, |
| 8085105 | probable manganese-transporting ATPase PDR2(LOC8085105) | *Sorghum bicolor* | GO:0006874~cellular calcium ion homeostasis,GO:0009846~pollen germination,GO:0010073~meristem maintenance,GO:0010152~pollen maturation,GO:0016036~cellular response to phosphate starvation,GO:0048867~stem cell fate determination, |
| 4337170 | probable pectinesterase/pectinesterase inhibitor 13(LOC4337170) | *Oryza sativa Japonica Group* | GO:0042545~cell wall modification,GO:0045490~pectin catabolic process, |
| 4344030 | probable transcription factor At5g28040(LOC4344030) | *Oryza sativa Japonica Group* | GO:0006355~regulation of transcription, DNA-templated, |
| 4342431 | protein ETHYLENE-INSENSITIVE 2-like(LOC4342431) | *Oryza sativa Japonica Group* | GO:0009873~ethylene-activated signaling pathway,GO:0010104~regulation of ethylene-activated signaling pathway, |
| 8072447 | protein PIN-LIKES 2(LOC8072447) | *Sorghum bicolor* | GO:0009734~auxin-activated signaling pathway,GO:0010252~auxin homeostasis,GO:0010311~lateral root formation,GO:0040009~regulation of growth rate, |
| 8064218 | putative linoleate 9S-lipoxygenase 3(LOC8064218) | *Sorghum bicolor* | GO:0006633~fatty acid biosynthetic process,GO:0031408~oxylipin biosynthetic process,GO:0034440~lipid oxidation, |
| 110432163 | putative receptor-like protein kinase At4g00960(LOC110432163) | *Sorghum bicolor* | GO:0006468~protein phosphorylation, |
| 843258 | receptor-like protein kinase 1(RPK1) | *Arabidopsis thaliana* | GO:0006468~protein phosphorylation,GO:0009409~response to cold,GO:0009414~response to water deprivation,GO:0009651~response to salt stress,GO:0009737~response to abscisic acid,GO:0009738~abscisic acid-activated signaling pathway,GO:0009942~longitudinal axis specification,GO:0009945~radial axis specification,GO:0048508~embryonic meristem development, |
| 838452 | regulatory component of ABA receptor 1(RCAR1) | *Arabidopsis thaliana* | GO:0009738~abscisic acid-activated signaling pathway,GO:0080163~regulation of protein serine/threonine phosphatase activity, |
| 834842 | respiratory burst oxidase homologue D(RBOHD) | *Arabidopsis thaliana* | GO:0006952~defense response,GO:0007231~osmosensory signaling pathway,GO:0009408~response to heat,GO:0009611~response to wounding,GO:0033500~carbohydrate homeostasis,GO:0043069~negative regulation of programmed cell death,GO:0050832~defense response to fungus,GO:0071456~cellular response to hypoxia,GO:0072593~reactive oxygen species metabolic process, |
| 842710 | respiratory burst oxidase protein F(RBOH F) | *Arabidopsis thaliana* | GO:0002679~respiratory burst involved in defense response,GO:0006952~defense response,GO:0007231~osmosensory signaling pathway,GO:0009723~response to ethylene,GO:0009738~abscisic acid-activated signaling pathway,GO:0009873~ethylene-activated signaling pathway,GO:0010119~regulation of stomatal movement,GO:0033500~carbohydrate homeostasis,GO:0043069~negative regulation of programmed cell death,GO:0050665~hydrogen peroxide biosynthetic process,GO:0052542~defense response by callose deposition,GO:0072593~reactive oxygen species metabolic process, |
| 100381320 | ripening-related protein(LOC100381320) | *Zea mays* | GO:0043086~negative regulation of catalytic activity, |
| 8055864 | tonoplast dicarboxylate transporter(LOC8055864) | *Sorghum bicolor* | GO:0098656~anion transmembrane transport, |
| 8075588 | transcription factor VOZ1(LOC8075588) | *Sorghum bicolor* | GO:0045893~positive regulation of transcription, DNA-templated,GO:0048578~positive regulation of long-day photoperiodism, flowering, |
| 844194 | trehalose-6-phosphate synthase(TPS1) | *Arabidopsis thaliana* | GO:0005991~trehalose metabolic process,GO:0005992~trehalose biosynthetic process,GO:0009793~embryo development ending in seed dormancy,GO:0009832~plant-type cell wall biogenesis,GO:0010182~sugar mediated signaling pathway,GO:0048364~root development,GO:0048574~long-day photoperiodism, flowering,GO:0051301~cell division,GO:0080186~developmental vegetative growth, |
| 819323 | ubiquitin 6(UBQ6) | *Arabidopsis thaliana* | GO:0006412~translation,GO:0006511~ubiquitin-dependent protein catabolic process,GO:0016567~protein ubiquitination,GO:0019941~modification-dependent protein catabolic process, |
| 100275707 | uncharacterized LOC100275707(LOC100275707) | *Zea mays* | GO:0010468~regulation of gene expression, |
| 100279496 | uncharacterized LOC100279496(LOC100279496) | *Zea mays* | GO:0005975~carbohydrate metabolic process,GO:0046373~L-arabinose metabolic process, |
| 100283330 | uncharacterized LOC100283330(LOC100283330) | *Zea mays* | GO:0006811~ion transport, |
| 4327168 | uncharacterized LOC4327168(LOC4327168) | *Oryza sativa Japonica Group* | GO:0006952~defense response, |
| 8060854 | uncharacterized LOC8060854(LOC8060854) | *Sorghum bicolor* | GO:0033615~mitochondrial proton-transporting ATP synthase complex assembly, |
| 8075414 | uncharacterized LOC8075414(LOC8075414) | *Sorghum bicolor* | GO:0006629~lipid metabolic process, |
| 4347825 | uncharacterized protein At2g39795, mitochondrial(LOC4347825) | *Oryza sativa Japonica Group* | GO:0070131~positive regulation of mitochondrial translation, |

| **Supplementary Table 6.** 87 Genes with known GO terms enriched in different biological processes using UP_KW_BIOLOGICAL_PROCESS | | | | |
| --- | --- | --- | --- | --- |
| SN | Entrez_ID | Species | Gene Name | UP_KW_BIOLOGICAL_PROCESS |
| 1 | 828714 | *Arabidopsis thaliana* | Protein phosphatase 2C family protein(ABI1) | Abscisic acid signaling pathway, |
| 2 | 835809 | *Arabidopsis thaliana* | Protein phosphatase 2C family protein(ABI2) | Abscisic acid signaling pathway, |
| 3 | 841670 | *Arabidopsis thaliana* | beta glucosidase 18(BGLU18) | Abscisic acid signaling pathway, |
| 4 | 843258 | *Arabidopsis thaliana* | receptor-like protein kinase 1(RPK1) | Abscisic acid signaling pathway, |
| 5 | 838452 | *Arabidopsis thaliana* | regulatory component of ABA receptor 1(RCAR1) | Abscisic acid signaling pathway, |
| 6 | 8058459 | *Sorghum bicolor* | 4-hydroxy-tetrahydrodipicolinate synthase, chloroplastic(LOC8058459) | Amino-acid biosynthesis, Diaminopimelate biosynthesis, Lysine biosynthesis, |
| 7 | 817257 | *Arabidopsis thaliana* | abscisic aldehyde oxidase 3(AAO3) | Auxin biosynthesis, Abscisic acid biosynthesis, |
| 8 | 8072447 | *Sorghum bicolor* | protein PIN-LIKES 2(LOC8072447) | Auxin signaling pathway, |
| 9 | 4349916 | *Oryza sativa Japonica Group* | probable alkaline/neutral invertase F(LOC4349916) | Carbohydrate metabolism, |
| 10 | 819331 | *Arabidopsis thaliana* | galactinol synthase 1(GolS1) | Carbohydrate metabolism, Galactose metabolism, |
| 11 | 842114 | *Arabidopsis thaliana* | galactinol synthase 2(GolS2) | Carbohydrate metabolism, Galactose metabolism, |
| 12 | 837576 | *Arabidopsis thaliana* | Glutathione S-transferase family protein(ERD9) | Detoxification, Growth regulation, Stress response, |
| 13 | 4352458 | *Oryza sativa Japonica Group* | condensin-2 complex subunit D3(LOC4352458) | DNA condensation, |
| 14 | 826730 | *Arabidopsis thaliana* | 1-aminocyclopropane-1-carboxylic acid (acc) synthase 6(ACS6) | Ethylene biosynthesis, Fruit ripening, Plant defense, |
| 15 | 8083691 | *Sorghum bicolor* | ETHYLENE INSENSITIVE 3-like 1 protein(LOC8083691) | Ethylene signaling pathway, |
| 16 | 4342431 | *Oryza sativa Japonica Group* | protein ETHYLENE-INSENSITIVE 2-like(LOC4342431) | Ethylene signaling pathway, |
| 17 | 817876 | *Arabidopsis thaliana* | peroxisomal 3-ketoacyl-CoA thiolase 3(PKT3) | Fatty acid biosynthesis, Fatty acid metabolism,Lipid metabolism,Lipid biosynthesis,Oxylipin biosynthesis, |
| 18 | 8064218 | *Sorghum bicolor* | putative linoleate 9S-lipoxygenase 3(LOC8064218) | Fatty acid metabolism, Lipid metabolism, |
| 19 | 831241 | *Arabidopsis thaliana* | Chalcone and stilbene synthase family protein(TT4) | Flavonoid biosynthesis, |
| 20 | 4347311 | *Oryza sativa Japonica Group* | glucose-6-phosphate isomerase 1, chloroplastic(LOC4347311) | Glycolysis, |
| 21 | 838138 | *Arabidopsis thaliana* | Inorganic H pyrophosphatase family protein(AVP1) | Hydrogen ion transport, Ion transport, Transport, |
| 22 | 816413 | *Arabidopsis thaliana* | H[+]-ATPase 1(HA1) | Hydrogen ion transport, Ion transport,Transport, |
| 23 | 820121 | *Arabidopsis thaliana* | ascorbate peroxidase 2(APX2) | Hydrogen peroxide, |
| 24 | 827555 | *Arabidopsis thaliana* | potassium channel KAT1-like protein(KAT2) | Ion transport,Potassium transport,Transport, |
| 25 | 8620286 | *Dictyostelium discoideum AX4* | CLC 6/7 family protein(clcC) | Ion transport,Transport, |
| 26 | 100283330 | *Zea mays* | uncharacterized LOC100283330(LOC100283330) | Ion transport,Transport, |
| 27 | 8068630 | *Sorghum bicolor* | disease resistance protein RPS2(LOC8068630) | Plant defense, |
| 28 | 832311 | *Arabidopsis thaliana* | fatty acid reductase 1(FAR1) | Lipid metabolism,Lipid biosynthesis, |
| 29 | 834443 | *Arabidopsis thaliana* | CAP-binding protein 20(CBP20) | mRNA processing, mRNA splicing, RNA-mediated gene silencing, |
| 30 | 4342173 | *Oryza sativa Japonica Group* | potassium transporter 22-like(LOC4342173) | on transport,Potassium transport,Transport, |
| 31 | 821781 | *Arabidopsis thaliana* | far-red elongated hypocotyls 3(FHY3) | Phytochrome signaling pathway,Transcription,Transcription regulation, |
| 32 | 834842 | *Arabidopsis thaliana* | respiratory burst oxidase homologue D(RBOHD) | Plant defense, |
| 33 | 829541 | *Arabidopsis thaliana* | Protein kinase superfamily protein(OST1) | Plant defense,Abscisic acid signaling pathway, |
| 34 | 836865 | *Arabidopsis thaliana* | myb domain protein r1(MYBR1) | Plant defense,Transcription,Transcription regulation,Abscisic acid signaling pathway,Jasmonic acid signaling pathway, |
| 35 | 4331811 | *Oryza sativa Japonica Group* | elongation factor 1-alpha-like(LOC4331811) | Protein biosynthesis, |
| 36 | 4332538 | *Oryza sativa Japonica Group* | eukaryotic translation initiation factor 2 subunit alpha homolog(LOC4332538) | Protein biosynthesis, |
| 37 | 110431928 | *Sorghum bicolor* | TOM1-like protein 2(LOC110431928) | Protein transport,Transport, |
| 38 | 841514 | *Arabidopsis thaliana* | Nucleolar GTP-binding protein(AT1G50920) | Ribosome biogenesis, |
| 39 | 835180 | *Arabidopsis thaliana* | Clp ATPase(ERD1) | Stress response, |
| 40 | 4346187 | *Oryza sativa Japonica Group* | calcium-dependent protein kinase 21-like(LOC4346187) | Stress response, |
| 41 | 103649807 | *Zea mays* | class I heat shock protein pseudogene(LOC103649807) | Stress response, |
| 42 | 827663 | *Arabidopsis thaliana* | cytochrome P450, family 707, subfamily A, polypeptide 1(CYP707A1) | Stress response, |
| 43 | 834570 | *Arabidopsis thaliana* | cytochrome P450, family 707, subfamily A, polypeptide 3(CYP707A3) | Stress response, |
| 44 | 100383187 | *Zea mays* | uncharacterized LOC100383187(LOC100383187) | Stress response, |
| 45 | 4341520 | *Oryza sativa Japonica Group* | zinc finger A20 and AN1 domain-containing stress-associated protein 8(LOC4341520) | Stress response, |
| 46 | 820667 | *Arabidopsis thaliana* | nine-cis-epoxycarotenoid dioxygenase 3(NCED3) | Stress response, Abscisic acid biosynthesis, |
| 47 | 818936 | *Arabidopsis thaliana* | glutathione peroxidase 3(GPX3) | Stress response,Abscisic acid signaling pathway, |
| 48 | 831889 | *Arabidopsis thaliana* | NRAMP metal ion transporter family protein(EIN2) | Stress response,Plant defense,Auxin signaling pathway, Cytokinin signaling pathway, Ethylene signaling pathway, |
| 49 | 828651 | *Arabidopsis thaliana* | C-repeat/DRE binding factor 2(CBF2) | Stress response, Transcription,Transcription regulation, |
| 50 | 830424 | *Arabidopsis thaliana* | DRE-binding protein 2A(DREB2A) | Stress response,Transcription,Transcription regulation, |
| 51 | 4334553 | *Oryza sativa Japonica Group* | NAC domain-containing protein 2-like(LOC4334553) | Stress response,Transcription,Transcription regulation, |
| 52 | 4339974 | *Oryza sativa Japonica Group* | dehydration-responsive element-binding protein 1C(LOC4339974) | Stress response,Transcription,Transcription regulation, |
| 53 | 4324418 | *Oryza sativa Japonica Group* | dehydration-responsive element-binding protein 2A-like(LOC4324418) | Stress response,Transcription,Transcription regulation, |
| 54 | 4330838 | *Oryza sativa Japonica Group* | bZIP transcription factor 23-like(LOC4330838) | Stress response,Transcription,Transcription regulation,Abscisic acid signaling pathway, |
| 55 | 4331194 | *Oryza sativa Japonica Group* | aquaporin PIP 1-3-like(LOC4331194) | Stress response,Transport, |
| 56 | 843527 | *Arabidopsis thaliana* | ATP-binding casette family G25(ABCG25) | Stress response,Transport,Abscisic acid signaling pathway, |
| 57 | 4347620 | *Oryza sativa Japonica Group* | dehydration-responsive element-binding protein 1A-like(LOC4347620) | Stress responseTranscription,Transcription regulation, |
| 58 | 4344714 | *Oryza sativa Japonica Group* | AT-hook motif nuclear-localized protein 23(LOC4344714) | Transcription,Transcription regulation, |
| 59 | 542390 | *Zea mays* | CAAT-box DNA binding protein subunit B (NF-YB)(LOC542390) | Transcription,Transcription regulation, |
| 60 | 835726 | *Arabidopsis thaliana* | WRKY DNA-binding protein 2(WRKY2) | Transcription,Transcription regulation, |
| 61 | 4324824 | *Oryza sativa Japonica Group* | WUSCHEL-related homeobox 9-like(LOC4324824) | Transcription,Transcription regulation, |
| 62 | 110429775 | *Sorghum bicolor* | bZIP transcription factor 68-like(LOC110429775) | Transcription,Transcription regulation, |
| 63 | 8082391 | *Sorghum bicolor* | ethylene-responsive transcription factor 4(LOC8082391) | Transcription,Transcription regulation, |
| 64 | 829969 | *Arabidopsis thaliana* | histone deacetylase 1(HD1) | Transcription,Transcription regulation, |
| 65 | 836431 | *Arabidopsis thaliana* | histone deacetylase 6(HDA6) | Transcription,Transcription regulation, |
| 66 | 843671 | *Arabidopsis thaliana* | homeodomain GLABROUS 11(HDG11) | Transcription,Transcription regulation, |
| 67 | 8061169 | *Sorghum bicolor* | light-inducible protein CPRF2(LOC8061169) | Transcription,Transcription regulation, |
| 68 | 837403 | *Arabidopsis thaliana* | myb domain protein 60(MYB60) | Transcription,Transcription regulation, |
| 69 | 4326871 | *Oryza sativa Japonica Group* | ocs element-binding factor 1(LOC4326871) | Transcription,Transcription regulation, |
| 70 | 840158 | *Arabidopsis thaliana* | Basic helix-loop-helix (bHLH) DNA-binding family protein(MYC2) | Transcription,Transcription regulation,Abscisic acid signaling pathway,Jasmonic acid signaling pathway, |
| 71 | 100125659 | *Zea mays* | ABC transporter C family MRP4(LOC100125659) | Transport, |
| 72 | 830541 | *Arabidopsis thaliana* | ABC-2 type transporter family protein(ABCG22) | Transport, |
| 73 | 8068578 | *Sorghum bicolor* | AP3-complex subunit beta-A(LOC8068578) | Transport, |
| 74 | 4330248 | *Oryza sativa Japonica Group* | aquaporin PIP1-1-like(LOC4330248) | Transport, |
| 75 | 4331362 | *Oryza sativa Japonica Group* | non-specific lipid-transfer protein 2-like(LOC4331362) | Transport, |
| 76 | 825316 | *Arabidopsis thaliana* | plasma membrane intrinsic protein 1A(PIP1A) | Transport, |
| 77 | 824510 | *Arabidopsis thaliana* | plasma membrane intrinsic protein 2A(PIP2A) | Transport, |
| 78 | 100281081 | *Zea mays* | potassium transporter 10(LOC100281081) | Transport, |
| 79 | 8061071 | *Sorghum bicolor* | probable ADP,ATP carrier protein At5g56450(LOC8061071) | Transport, |
| 80 | 4330049 | *Oryza sativa Japonica Group* | probable aquaporin PIP2-2(LOC4330049) | Transport, |
| 81 | 4339571 | *Oryza sativa Japonica Group* | probable purine permease 4(LOC4339571) | Transport, |
| 82 | 828653 | *Arabidopsis thaliana* | C-repeat/DRE binding factor 1(CBF1) | Stress response, Transcription, Transcription regulation, |
| 83 | 4345657 | *Oryza sativa Japonica Group* | malate dehydrogenase, chloroplastic(LOC4345657) | Tricarboxylic acid cycle, |
| 84 | 831213 | *Arabidopsis thaliana* | IKI3 family protein(ABO1) | tRNA processing, Auxin signaling pathway, Abscisic acid signaling pathway, |
| 85 | 820292 | *Arabidopsis thaliana* | Paxneb protein-like protein(ELO1) | tRNA processing, Auxin signaling pathway,Abscisic acid signaling pathway, |
| 86 | 4344172 | *Oryza sativa Japonica Group* | E3 ubiquitin-protein ligase DIS1-like(LOC4344172) | Ubl conjugation pathway, |
| 87 | 839226 | *Arabidopsis thaliana* | cullin 3(CUL3) | Ubl conjugation pathway, |

| **Supplementary Table 7.** 102 Genes with known GO terms enriched in different molecular function using UP_KW_MOLECULAR_FUNCTION | | | | |
| --- | --- | --- | --- | --- |
| SN | ID | Gene Name | Species | UP_KW_MOLECULAR_FUNCTION |
| 1 | 840158 | Basic helix-loop-helix (bHLH) DNA-binding family protein(MYC2) | *Arabidopsis thaliana* | Activator, DNA-binding |
| 2 | 828653 | C-repeat/DRE binding factor 1(CBF1) | *Arabidopsis thaliana* | Activator, DNA-binding |
| 3 | 828651 | C-repeat/DRE binding factor 2(CBF2) | *Arabidopsis thaliana* | Activator, DNA-binding |
| 4 | 542390 | CAAT-box DNA binding protein subunit B (NF-YB) | *Zea mays* | Activator, DNA-binding |
| 5 | 830424 | DRE-binding protein 2A(DREB2A) | *Arabidopsis thaliana* | Activator, DNA-binding |
| 6 | 4334553 | NAC domain-containing protein 2-like | *Oryza sativa Japonica Group* | Activator, DNA-binding |
| 7 | 4347620 | dehydration-responsive element-binding protein 1A-like | *Oryza sativa Japonica Group* | Activator, DNA-binding |
| 8 | 4339974 | dehydration-responsive element-binding protein 1C | *Oryza sativa Japonica Group* | Activator, DNA-binding |
| 9 | 4324418 | dehydration-responsive element-binding protein 2A-like | *Oryza sativa Japonica Group* | Activator, DNA-binding |
| 10 | 836865 | myb domain protein r1(MYBR1) | *Arabidopsis thaliana* | Activator,DNA-binding |
| 11 | 831241 | Chalcone and stilbene synthase family protein(TT4) | *Arabidopsis thaliana* | Acyltransferase, Transferase |
| 12 | 817876 | peroxisomal 3-ketoacyl-CoA thiolase 3(PKT3) | *Arabidopsis thaliana* | Acyltransferase, Transferase |
| 13 | 4338289 | probable glycerol-3-phosphate acyltransferase 3 | *Oryza sativa Japonica Group* | Acyltransferase, Transferase |
| 14 | 100283536 | uncharacterized | *Zea mays* | Acyltransferase, Transferase |
| 15 | 100284805 | 10-deacetylbaccatin III 10-O-acetyltransferase | *Zea mays* | Acyltransferase,Transferase |
| 16 | 4337170 | probable pectinesterase/pectinesterase inhibitor 13 | *Oryza sativa Japonica Group* | Aspartyl esterase,Hydrolase |
| 17 | 8055854 | heat shock protein 81-2 | *Sorghum bicolor* | Chaperone |
| 18 | 835180 | Clp ATPase(ERD1) | *Arabidopsis thaliana* | Chaperone, Hydrolase |
| 19 | 831889 | NRAMP metal ion transporter family protein(EIN2) | *Arabidopsis thaliana* | Chromatin regulator |
| 20 | 836431 | histone deacetylase 6(HDA6) | *Arabidopsis thaliana* | Chromatin regulator, Hydrolase, Repressor |
| 21 | 829969 | histone deacetylase 1(HD1) | *Arabidopsis thaliana* | Chromatin regulator, Hydrolase,Repressor |
| 22 | 820292 | Paxneb protein-like protein(ELO1) | *Arabidopsis thaliana* | Developmental protein,Developmental protein |
| 23 | 835726 | WRKY DNA-binding protein 2(WRKY2) | *Arabidopsis thaliana* | Developmental protein,DNA-binding,Developmental protein |
| 24 | 4324824 | WUSCHEL-related homeobox 9-like(LOC4324824) | *Oryza sativa Japonica Group* | Developmental protein,DNA-binding,Developmental protein |
| 25 | 843258 | receptor-like protein kinase 1(RPK1) | *Arabidopsis thaliana* | Developmental protein,Kinase,Receptor,Serine/threonine-protein kinase,Transferase,Developmental protein |
| 26 | 8064218 | putative linoleate 9S-lipoxygenase 3 | *Sorghum bicolor* | Dioxygenase |
| 27 | 820667 | nine-cis-epoxycarotenoid dioxygenase 3(NCED3) | *Arabidopsis thaliana* | Dioxygenase,Oxidoreductase |
| 28 | 4344714 | AT-hook motif nuclear-localized protein 23 | *Oryza sativa Japonica Group* | DNA-binding |
| 29 | 8067406 | auxin response factor 22 | *Sorghum bicolor* | DNA-binding |
| 30 | 4330838 | bZIP transcription factor 23-like | *Oryza sativa Japonica Group* | DNA-binding |
| 31 | 110429775 | bZIP transcription factor 68-like | *Sorghum bicolor* | DNA-binding |
| 32 | 8082122 | cyclic dof factor 1 | *Sorghum bicolor* | DNA-binding |
| 33 | 8078579 | cyclic dof factor 2 | *Sorghum bicolor* | DNA-binding |
| 34 | 8082391 | ethylene-responsive transcription factor 4 | *Sorghum bicolor* | DNA-binding |
| 35 | 103641629 | histone H3.2 | *Zea mays* | DNA-binding |
| 36 | 843671 | homeodomain GLABROUS 11(HDG11) | *Arabidopsis thaliana* | DNA-binding |
| 37 | 8061169 | light-inducible protein CPRF2 | *Sorghum bicolor* | DNA-binding |
| 38 | 837403 | myb domain protein 60(MYB60) | *Arabidopsis thaliana* | DNA-binding |
| 39 | 4326871 | ocs element-binding factor 1 | *Oryza sativa Japonica Group* | DNA-binding |
| 40 | 4337721 | probable inactive DNA (cytosine-5)-methyltransferase DRM3 | *Oryza sativa Japonica Group* | DNA-binding,Methyltransferase,Transferase |
| 41 | 100275707 | uncharacterized | *Zea mays* | Endonuclease,Hydrolase,Nuclease |
| 42 | 841670 | beta glucosidase 18(BGLU18) | *Arabidopsis thaliana* | Glycosidase,Hydrolase |
| 43 | 4346248 | beta-galactosidase 11-like | *Oryza sativa Japonica Group* | Glycosidase,Hydrolase |
| 44 | 4336181 | glycosyl hydrolase 5 family protein | *Oryza sativa Japonica Group* | Glycosidase,Hydrolase |
| 45 | 4349916 | probable alkaline/neutral invertase F | *Oryza sativa Japonica Group* | Glycosidase,Hydrolase |
| 46 | 819331 | galactinol synthase 1(GolS1) | *Arabidopsis thaliana* | Glycosyltransferase,Transferase |
| 47 | 842114 | galactinol synthase 2(GolS2) | *Arabidopsis thaliana* | Glycosyltransferase,Transferase |
| 48 | 4324110 | gallate 1-beta-glucosyltransferase | *Oryza sativa Japonica Group* | Glycosyltransferase,Transferase |
| 49 | 844194 | trehalose-6-phosphate synthase(TPS1) | *Arabidopsis thaliana* | Glycosyltransferase,Transferase |
| 50 | 8066485 | DExH-box ATP-dependent RNA helicase DExH12 | *Sorghum bicolor* | Helicase, Hydrolase |
| 51 | 8078643 | probable protein phosphatase 2C 6 | *Sorghum bicolor* | Hydrolase, |
| 52 | 828714 | Protein phosphatase 2C family protein(ABI1) | *Arabidopsis thaliana* | Hydrolase, Protein phosphatase |
| 53 | 835809 | Protein phosphatase 2C family protein(ABI2) | *Arabidopsis thaliana* | Hydrolase, Protein phosphatase |
| 54 | 100217031 | uncharacterized | *Zea mays* | Hydrolase,KKinase,Receptor,Transferase |
| 55 | 827555 | potassium channel KAT1-like protein(KAT2) | *Arabidopsis thaliana* | Ion channel,Potassium channel,Voltage-gated channel |
| 56 | 8620286 | CLC 6/7 family protein(clcC) | *Dictyostelium discoideum AX4* | Ion channel,Voltage-gated channel, Chloride channel |
| 57 | 829541 | Protein kinase superfamily protein(OST1) | *Arabidopsis thaliana* | Kinase, Serine/threonine-protein kinase,Transferase |
| 58 | 844164 | Protein kinase superfamily protein(SNRK2-8) | *Arabidopsis thaliana* | Kinase, Serine/threonine-protein kinase,Transferase |
| 59 | 840471 | calcium-dependent protein kinase 2(CDPK2) | *Arabidopsis thaliana* | Kinase, Serine/threonine-protein kinase,Transferase |
| 60 | 825807 | calcium-dependent protein kinase 21(CPK21) | *Arabidopsis thaliana* | Kinase, Serine/threonine-protein kinase,Transferase |
| 61 | 4346187 | calcium-dependent protein kinase 21-like | *Oryza sativa Japonica Group* | Kinase, Serine/threonine-protein kinase,Transferase |
| 62 | 825809 | calcium-dependent protein kinase 23(CPK23) | *Arabidopsis thaliana* | Kinase, Serine/threonine-protein kinase,Transferase |
| 63 | 8062168 | receptor-like protein kinase HSL1 | *Sorghum bicolor* | Kinase, Serine/threonine-protein kinase,Transferase |
| 64 | 827604 | CBL-interacting protein kinase 12(CIPK12) | *Arabidopsis thaliana* | Kinase,Serine/threonine-protein kinase,Transferase |
| 65 | 830556 | CBL-interacting protein kinase 15(CIPK15) | *Arabidopsis thaliana* | Kinase,Serine/threonine-protein kinase,Transferase |
| 66 | 8067611 | CBL-interacting protein kinase 15 | *Sorghum bicolor* | Kinase,Serine/threonine-protein kinase,Transferase |
| 67 | 9639733 | CBL-interacting protein kinase 23 | *Selaginella moellendorffii* | Kinase,Serine/threonine-protein kinase,Transferase |
| 68 | 4335831 | G-type lectin S-receptor-like serine/threonine-protein kinase | *Oryza sativa Japonica Group* | Kinase,Transferase |
| 69 | 8059924 | L-type lectin-domain containing receptor kinase IV.1 | *Sorghum bicolor* | Kinase,Transferase |
| 70 | 110432163 | putative receptor-like protein kinase | *Sorghum bicolor* | Kinase,Transferase |
| 71 | 107278728 | rust resistance kinase Lr10 | *Oryza sativa Japonica Group* | Kinase,Transferase |
| 72 | 4347311 | glucose-6-phosphate isomerase 1, chloroplastic | *Oryza sativa Japonica Group* | KW-0413~Isomerase, |
| 73 | 826730 | 1-aminocyclopropane-1-carboxylic acid (acc) synthase 6(ACS6) | *Arabidopsis thaliana* | Lyase |
| 74 | 827663 | cytochrome P450, family 707, subfamily A, polypeptide 1(CYP707A1) | *Arabidopsis thaliana* | Monooxygenase, Oxidoreductase |
| 75 | 834570 | cytochrome P450, family 707, subfamily A, polypeptide 3(CYP707A3) | *Arabidopsis thaliana* | Monooxygenase, Oxidoreductase |
| 76 | 842213 | FAD/NAD(P)-binding oxidoreductase family protein(XF1) | *Arabidopsis thaliana* | Oxidoreductase |
| 77 | 4326769 | NADP-dependent malic enzyme, chloroplastic-like | *Oryza sativa Japonica Group* | Oxidoreductase |
| 78 | 110436990 | UDP-glucose 6-dehydrogenase 5 | *Sorghum bicolor* | Oxidoreductase |
| 79 | 817257 | abscisic aldehyde oxidase 3(AAO3) | *Arabidopsis thaliana* | Oxidoreductase |
| 80 | 832311 | fatty acid reductase 1(FAR1) | *Arabidopsis thaliana* | Oxidoreductase |
| 81 | 4345657 | malate dehydrogenase, chloroplastic | *Oryza sativa Japonica Group* | Oxidoreductase |
| 82 | 8057594 | polyphenol oxidase I, chloroplastic | *Sorghum bicolor* | Oxidoreductase |
| 83 | 820121 | ascorbate peroxidase 2(APX2) | *Arabidopsis thaliana* | Oxidoreductase, Peroxidase |
| 84 | 818936 | glutathione peroxidase 3(GPX3) | *Arabidopsis thaliana* | Oxidoreductase, Peroxidase |
| 85 | 834842 | respiratory burst oxidase homologue D(RBOHD) | *Arabidopsis thaliana* | Oxidoreductase, Peroxidase |
| 86 | 842710 | respiratory burst oxidase protein F(RBOH F) | *Arabidopsis thaliana* | Oxidoreductase, Peroxidase |
| 87 | 838452 | regulatory component of ABA receptor 1(RCAR1) | *Arabidopsis thaliana* | Protein phosphatase inhibitor,Receptor |
| 88 | 4337526 | 50S ribosomal protein L28, chloroplastic | *Oryza sativa Japonica Group* | Ribonucleoprotein,Ribosomal protein |
| 89 | 8062999 | 60S ribosomal protein L10 | *Sorghum bicolor* | Ribonucleoprotein,Ribosomal protein |
| 90 | 103642077 | 60S ribosomal protein L27-3 | *Zea mays* | Ribonucleoprotein,Ribosomal protein |
| 91 | 100193672 | 60S ribosomal protein L35a-like | *Zea mays* | Ribonucleoprotein,Ribosomal protein |
| 92 | 819323 | ubiquitin 6(UBQ6) | *Arabidopsis thaliana* | Ribonucleoprotein,Ribosomal protein |
| 93 | 834443 | CAP-binding protein 20(CBP20) | *Arabidopsis thaliana* | RNA-binding |
| 94 | 4344172 | E3 ubiquitin-protein ligase DIS1-like | *Oryza sativa Japonica Group* | Transferase |
| 95 | 837576 | Glutathione S-transferase family protein(ERD9) | *Arabidopsis thaliana* | Transferase |
| 96 | 816413 | H[+]-ATPase 1(HA1) | *Arabidopsis thaliana* | Transferase |
| 97 | 838138 | Inorganic H pyrophosphatase family protein(AVP1) | *Arabidopsis thaliana* | Transferase |
| 98 | 4329854 | beta-1,4-mannosyl-glycoprotein 4-beta-N-acetylglucosaminyltransferase | *Oryza sativa Japonica Group* | Transferase |
| 99 | 4343863 | dehydrodolichyl diphosphate synthase 6 | *Oryza sativa Japonica Group* | Transferase |
| 100 | 100125659 | ABC transporter C family MRP4 | *Zea mays* | Translocase |
| 101 | 8085105 | probable manganese-transporting ATPase PDR2( | *Sorghum bicolor* | Translocase |
| 102 | 4332957 | RNA-binding protein CP29B, chloroplastic | *Oryza sativa Japonica Group* | Viral nucleoprotein, Ribonucleoprotein, RNA-binding |

| **Supplementary Table 8. UP_tissue report of gene expression analysis** | | | | |
| --- | --- | --- | --- | --- |
|  | **ID** | **Species** | **UP_TISSUE** | **Gene Name** |
| 1 | 4331811 | *Oryza sativa Japonica Group* | Booting stage seed, | elongation factor 1-alpha-like(LOC4331811) |
| 2 | 4330248 | *Oryza sativa Japonica Group* | Booting stage seed,Germinating seed,Immature seed,Meristem, | aquaporin PIP1-1-like(LOC4330248) |
| 3 | 4326769 | *Oryza sativa Japonica Group* | Callus, | NADP-dependent malic enzyme, chloroplastic-like(LOC4326769) |
| 4 | 4346187 | *Oryza sativa Japonica Group* | Flower, | calcium-dependent protein kinase 21-like(LOC4346187) |
| 5 | 4328666 | *Oryza sativa Japonica Group* | Immature seed, | transcription initiation factor TFIID subunit 15b(LOC4328666) |
| 6 | 826730 | *Arabidopsis thaliana* | Leaf, | 1-aminocyclopropane-1-carboxylic acid (acc) synthase 6(ACS6) |
| 7 | 840158 | *Arabidopsis thaliana* | Leaf, | Basic helix-loop-helix (bHLH) DNA-binding family protein(MYC2) |
| 8 | 831241 | *Arabidopsis thaliana* | Leaf, | Chalcone and stilbene synthase family protein(TT4) |
| 9 | 837576 | *Arabidopsis thaliana* | Leaf, | Glutathione S-transferase family protein(ERD9) |
| 10 | 838138 | *Arabidopsis thaliana* | Leaf, | Inorganic H pyrophosphatase family protein(AVP1) |
| 11 | 829541 | *Arabidopsis thaliana* | Leaf, | Protein kinase superfamily protein(OST1) |
| 12 | 844164 | *Arabidopsis thaliana* | Leaf, | Protein kinase superfamily protein(SNRK2-8) |
| 13 | 835809 | *Arabidopsis thaliana* | Leaf, | Protein phosphatase 2C family protein(ABI2) |
| 14 | 820121 | *Arabidopsis thaliana* | Leaf, | ascorbate peroxidase 2(APX2) |
| 15 | 825807 | *Arabidopsis thaliana* | Leaf, | calcium-dependent protein kinase 21(CPK21) |
| 16 | 839226 | *Arabidopsis thaliana* | Leaf, | cullin 3(CUL3) |
| 17 | 827663 | *Arabidopsis thaliana* | Leaf, | cytochrome P450, family 707, subfamily A, polypeptide 1(CYP707A1) |
| 18 | 821781 | *Arabidopsis thaliana* | Leaf, | far-red elongated hypocotyls 3(FHY3) |
| 19 | 818936 | *Arabidopsis thaliana* | Leaf, | glutathione peroxidase 3(GPX3) |
| 20 | 829969 | *Arabidopsis thaliana* | Leaf, | histone deacetylase 1(HD1) |
| 21 | 837403 | *Arabidopsis thaliana* | Leaf, | myb domain protein 60(MYB60) |
| 22 | 836865 | *Arabidopsis thaliana* | Leaf, | myb domain protein r1(MYBR1) |
| 23 | 817876 | *Arabidopsis thaliana* | Leaf, | peroxisomal 3-ketoacyl-CoA thiolase 3(PKT3) |
| 24 | 825316 | *Arabidopsis thaliana* | Leaf, | plasma membrane intrinsic protein 1A(PIP1A) |
| 25 | 824510 | *Arabidopsis thaliana* | Leaf, | plasma membrane intrinsic protein 2A(PIP2A) |
| 26 | 838452 | *Arabidopsis thaliana* | Leaf, | regulatory component of ABA receptor 1(RCAR1) |
| 27 | 844194 | *Arabidopsis thaliana* | Leaf, | trehalose-6-phosphate synthase(TPS1) |
| 28 | 819323 | *Arabidopsis thaliana* | Leaf, | ubiquitin 6(UBQ6) |
| 29 | 816413 | *Arabidopsis thaliana* | Leaf,Root,Seedling, | H[+]-ATPase 1(HA1) |
| 30 | 836431 | *Arabidopsis thaliana* | Leaf,Roots, | histone deacetylase 6(HDA6) |
| 31 | 4331362 | *Oryza sativa Japonica Group* | Seed, | non-specific lipid-transfer protein 2-like(LOC4331362) |
| 32 | 100284805 | *Zea mays* | Seedling, | 10-deacetylbaccatin III 10-O-acetyltransferase(LOC100284805) |
| 33 | 103642077 | *Zea mays* | Seedling, | 60S ribosomal protein L27-3(LOC103642077) |
| 34 | 100193672 | *Zea mays* | Seedling, | 60S ribosomal protein L35a-like(LOC100193672) |
| 35 | 100125659 | *Zea mays* | Seedling, | ABC transporter C family MRP4(LOC100125659) |
| 36 | 542390 | *Zea mays* | Seedling, | CAAT-box DNA binding protein subunit B (NF-YB)(LOC542390) |
| 37 | 100192929 | *Zea mays* | Seedling, | Ninja-family protein 1(LOC100192929) |
| 38 | 103637861 | *Zea mays* | Seedling, | Protein EXORDIUM(LOC103637861) |
| 39 | 100274751 | *Zea mays* | Seedling, | UDP-glucuronate 4-epimerase 1(LOC100274751) |
| 40 | 4331194 | *Oryza sativa Japonica Group* | Seedling, | aquaporin PIP 1-3-like(LOC4331194) |
| 41 | 103649807 | *Zea mays* | Seedling, | class I heat shock protein pseudogene(LOC103649807) |
| 42 | 103641629 | *Zea mays* | Seedling, | histone H3.2(LOC103641629) |
| 43 | 103635265 | *Zea mays* | Seedling, | methylesterase 7(LOC103635265) |
| 44 | 100281081 | *Zea mays* | Seedling, | potassium transporter 10(LOC100281081) |
| 45 | 100381320 | *Zea mays* | Seedling, | ripening-related protein(LOC100381320) |
| 46 | 100217031 | *Zea mays* | Seedling, | uncharacterized LOC100217031(LOC100217031) |
| 47 | 100274836 | *Zea mays* | Seedling, | uncharacterized LOC100274836(LOC100274836) |
| 48 | 100275707 | *Zea mays* | Seedling, | uncharacterized LOC100275707(LOC100275707) |
| 49 | 100279496 | *Zea mays* | Seedling, | uncharacterized LOC100279496(LOC100279496) |
| 50 | 100283536 | *Zea mays* | Seedling, | uncharacterized LOC100283536(LOC100283536) |
| 51 | 100383187 | *Zea mays* | Seedling, | uncharacterized LOC100383187(LOC100383187) |
| 52 | 103643967 | *Zea mays* | Seedling, | uncharacterized LOC103643967(LOC103643967) |
| 53 | 100857041 | *Zea mays* | Seedling,Vegetative tissue, | uncharacterized LOC100857041(LOC100857041) |

| **Supportive Table 9.** List of genes highly enrich in biological process using ShniyGO | | | | | |
| --- | --- | --- | --- | --- | --- |
| category | number_of_genes | Gene Names | p_value | FDR | description |
| Process | 13 | *MYB60,BGLU18,GolS2,XF1,RPK1,GolS1,NCED3,PIP1A,OST1,ABCG22,CYP707A3,ABI2,MYBR1* | 1.42E-14 | 4.69E-11 | Response to water deprivation |
| Process | 21 | *MYB60,BGLU18,GolS2,XF1,RPK1,ABCG25,TPS1,GolS1,ELO1,NCED3,AT3G58490,PIP1A,ABI1,OST1,CIPK15,EIN2,ABCG22,CYP707A3,ABI2,HDA6,MYBR1* | 9.98E-15 | 4.69E-11 | Response to oxygen-containing compound |
| Process | 22 | *MYB60,BGLU18,GolS2,XF1,RPK1,ABCG25,SNRK2-8,GolS1,NCED3,PIP1A,KAT2,CBF2,CBF1,ABI1,OST1,EIN2,ABCG22,CYP707A3,RBOHD,ABI2,HDA6,MYBR1* | 7.81E-14 | 7.35E-11 | Response to abiotic stimulus |
| Process | 14 | *MYB60,BGLU18,GolS2,RPK1,ABCG25,GolS1,ELO1,AT3G58490,ABI1,OST1,CIPK15,ABI2,HDA6,MYBR1* | 2.07E-13 | 1.41E-10 | Response to abscisic acid |
| Process | 33 | *MYB60,CDPK2,BGLU18,GolS2,XF1,RPK1,ABCG25,SNRK2-8,TPS1,bZIP23,PKT3,GolS1,ELO1,NCED3,AT3G58490,PIP1A,CPK21,CPK23,KAT2,CIPK12,CBF2,CBF1,ABI1,OST1,HD1,CIPK15,EIN2,ABCG22,CYP707A3,RBOHD,ABI2,HDA6,MYBR1* | 1.80E-13 | 1.41E-10 | Response to stimulus |
| Process | 24 | *MYB60,BGLU18,GolS2,XF1,RPK1,ABCG25,TPS1,bZIP23,PKT3,GolS1,ELO1,NCED3,AT3G58490,PIP1A,ABI1,OST1,CIPK15,EIN2,ABCG22,CYP707A3,RBOHD,ABI2,HDA6,MYBR1* | 1.64E-12 | 8.58E-10 | Response to chemical |
| Process | 25 | *MYB60,BGLU18,GolS2,XF1,RPK1,ABCG25,SNRK2-8,TPS1,PKT3,GolS1,ELO1,NCED3,PIP1A,CBF2,CBF1,ABI1,OST1,HD1,EIN2,ABCG22,CYP707A3,RBOHD,ABI2,HDA6,MYBR1* | 8.16E-12 | 3.84E-09 | Response to stress |
| Process | 8 | *CDPK2,BGLU18,PKT3,ABI1,OST1,CIPK15,EIN2,ABI2* | 3.10E-11 | 1.21E-08 | Regulation of abscisic acid-activated signaling pathway |
| Process | 12 | *BGLU18,GolS2,RPK1,SNRK2-8,GolS1,NCED3,OST1,EIN2,RBOHD,ABI2,HDA6,MYBR1* | 4.01E-11 | 1.26E-08 | Response to osmotic stress |
| Process | 14 | *MYB60,BGLU18,GolS2,XF1,RPK1,bZIP23,GolS1,NCED3,PIP1A,OST1,ABCG22,CYP707A3,ABI2,MYBR1* | 9.48E-11 | 2.79E-08 | Response to inorganic substance |
| Process | 9 | *BGLU18,RPK1,ABCG25,ELO1,ABI1,OST1,CIPK15,ABI2,MYBR1* | 1.59E-10 | 4.39E-08 | Abscisic acid-activated signaling pathway |
| Process | 27 | *AGL28,MYB60,CDPK2,BGLU18,RPK1,ABCG25,SNRK2-8,TPS1,bZIP23,PKT3,ELO1,CPK21,CPK23,KAT2,CIPK12,CBF2,CBF1,ABI1,OST1,HD1,CIPK15,EIN2,RBOHD,WRKY2,ABI2,HDA6,MYBR1* | 1.71E-10 | 4.47E-08 | Regulation of cellular process |
| Process | 10 | *CDPK2,BGLU18,PKT3,ELO1,ABI1,OST1,CIPK15,EIN2,ABI2,MYBR1* | 2.27E-10 | 5.62E-08 | Regulation of signal transduction |
| Process | 28 | *AGL28,MYB60,CDPK2,BGLU18,RPK1,ABCG25,SNRK2-8,TPS1,bZIP23,PKT3,ELO1,CPK21,CPK23,KAT2,CIPK12,CBF2,CBF1,ABI1,OST1,HD1,CIPK15,EIN2,CBP20,RBOHD,WRKY2,ABI2,HDA6,MYBR1* | 2.89E-10 | 6.28E-08 | Regulation of biological process |
| Process | 29 | *AGL28,MYB60,CDPK2,BGLU18,RPK1,ABCG25,SNRK2-8,TPS1,bZIP23,AAO3,PKT3,ELO1,CPK21,CPK23,KAT2,CIPK12,CBF2,CBF1,ABI1,OST1,HD1,CIPK15,EIN2,CBP20,RBOHD,WRKY2,ABI2,HDA6,MYBR1* | 6.52E-10 | 1.23E-07 | Biological regulation |
| Process | 17 | *CDPK2,BGLU18,RPK1,ABCG25,SNRK2-8,TPS1,ELO1,CPK21,CPK23,CIPK12,ABI1,OST1,CIPK15,EIN2,RBOHD,ABI2,MYBR1* | 1.33E-09 | 2.40E-07 | Signal transduction |
| Process | 5 | *BGLU18,AAO3,NCED3,CYP707A1,CYP707A3* | 1.69E-09 | 2.94E-07 | Abscisic acid metabolic process |
| Process | 11 | *MYB60,GolS2,RPK1,ABCG25,GolS1,CBF2,CBF1,ABI1,EIN2,RBOHD,ABI2* | 3.32E-09 | 5.04E-07 | Response to temperature stimulus |
| Process | 15 | *MYB60,BGLU18,GolS2,RPK1,ABCG25,GolS1,ELO1,AT3G58490,ABI1,OST1,CIPK15,EIN2,ABI2,HDA6,MYBR1* | 6.70E-09 | 9.84E-07 | Response to hormone |
| Process | 11 | *BGLU18,RPK1,ABCG25,TPS1,ELO1,ABI1,OST1,CIPK15,EIN2,ABI2,MYBR1* | 8.05E-09 | 1.15E-06 | Cellular response to oxygen-containing compound |
| Process | 11 | *MYB60,CDPK2,BGLU18,PKT3,ELO1,ABI1,OST1,CIPK15,EIN2,ABI2,MYBR1* | 2.14E-08 | 2.72E-06 | Regulation of response to stimulus |
| Process | 5 | *CDPK2,BGLU18,PKT3,OST1,EIN2* | 2.64E-08 | 3.18E-06 | Positive regulation of abscisic acid-activated signaling pathway |
| Process | 16 | *MYB60,BGLU18,GolS2,RPK1,ABCG25,TPS1,GolS1,ELO1,AT3G58490,ABI1,OST1,CIPK15,EIN2,ABI2,HDA6,MYBR1* | 2.74E-08 | 3.18E-06 | Response to organic substance |
| Process | 9 | *BGLU18,GolS2,RPK1,GolS1,NCED3,OST1,EIN2,HDA6,MYBR1* | 3.76E-08 | 4.11E-06 | Response to salt stress |
| Process | 19 | *CDPK2,BGLU18,RPK1,ABCG25,SNRK2-8,TPS1,PKT3,ELO1,CPK21,CPK23,CIPK12,ABI1,OST1,CIPK15,EIN2,CYP707A3,RBOHD,ABI2,MYBR1* | 5.49E-08 | 5.87E-06 | Cellular response to stimulus |
| Process | 6 | *CDPK2,BGLU18,PKT3,OST1,EIN2,MYBR1* | 5.71E-08 | 5.96E-06 | Positive regulation of signal transduction |
| Process | 38 | *MYB60,CDPK2,BGLU18,ZCF125,RPK1,ABCG25,SNRK2-8,TPS1,AAO3,PKT3,SPD1,ELO1,NCED3,AT3G58490,PIP1A,CPK21,CPK23,AT4G16720,KAT2,CIPK12,CYP707A1,ABI1,OST1,AT4G35910,HD1,CIPK15,EIN2,ABCG22,CBP20,CYP707A3,RBOHD,ERD1,WRKY2,ABI2,ABCA10,HDA6,MYBR1,YCF3* | 1.12E-07 | 1.08E-05 | Cellular process |
| Process | 14 | *BGLU18,RPK1,ABCG25,TPS1,PKT3,ELO1,ABI1,OST1,CIPK15,EIN2,CYP707A3,RBOHD,ABI2,MYBR1* | 1.15E-07 | 1.08E-05 | Cellular response to chemical stimulus |
| Process | 8 | *MYB60,GolS2,RPK1,ABCG25,GolS1,CBF2,CBF1,ABI1* | 3.30E-07 | 3.04E-05 | Response to cold |
| Process | 11 | *AGL28,CDPK2,BGLU18,PKT3,ELO1,CBF1,OST1,HD1,EIN2,HDA6,MYBR1* | 6.38E-07 | 5.77E-05 | Positive regulation of cellular process |
| Process | 5 | *BGLU18,ABI1,OST1,EIN2,ABI2* | 6.77E-07 | 6.01E-05 | Regulation of stomatal movement |
| Process | 11 | *BGLU18,RPK1,ABCG25,TPS1,ELO1,ABI1,OST1,CIPK15,EIN2,ABI2,MYBR1* | 1.30E-06 | 0.00011 | Cellular response to organic substance |
| Process | 10 | *BGLU18,RPK1,ABCG25,ELO1,ABI1,OST1,CIPK15,EIN2,ABI2,MYBR1* | 1.41E-06 | 0.00012 | Hormone-mediated signaling pathway |
| Process | 11 | *MYB60,ABCG25,ELO1,ABI1,HD1,CIPK15,EIN2,CBP20,RBOHD,ABI2,HDA6* | 1.38E-06 | 0.00012 | Negative regulation of biological process |
| Process | 27 | *CDPK2,BGLU18,GolS2,XF1,RPK1,SNRK2-8,TPS1,AAO3,PKT3,GolS1,ELO1,NCED3,AT3G58490,CPK21,CPK23,AT4G16720,CIPK12,CYP707A1,ABI1,OST1,AT4G35910,HD1,CIPK15,CBP20,CYP707A3,ABI2,HDA6* | 1.54E-06 | 0.00013 | Primary metabolic process |
| Process | 6 | *ABCG25,GolS1,ABI1,EIN2,RBOHD,ABI2* | 2.27E-06 | 0.00018 | Response to heat |
| Process | 4 | *MYB60,AT3G58490,KAT2,OST1* | 2.65E-06 | 0.00021 | Stomatal movement |
| Process | 30 | *CDPK2,BGLU18,GolS2,XF1,RPK1,SNRK2-8,TPS1,AAO3,CYP71A12,PKT3,GolS1,ELO1,NCED3,AT3G58490,CPK21,CPK23,AT4G16720,CIPK12,CYP707A1,ABI1,OST1,AT4G35910,HD1,CIPK15,CBP20,CYP707A3,RBOHD,ABI2,HDA6,YCF3* | 2.94E-06 | 0.00023 | Metabolic process |
| Process | 27 | *CDPK2,BGLU18,GolS2,XF1,RPK1,SNRK2-8,TPS1,AAO3,PKT3,GolS1,ELO1,NCED3,AT3G58490,CPK21,CPK23,AT4G16720,CIPK12,CYP707A1,ABI1,OST1,AT4G35910,HD1,CIPK15,CBP20,CYP707A3,ABI2,HDA6* | 9.26E-06 | 0.00065 | Organic substance metabolic process |
| Process | 3 | *NCED3,CYP707A1,CYP707A3* | 1.52E-05 | 0.001 | Isoprenoid catabolic process |
| Process | 7 | *BGLU18,AAO3,PKT3,NCED3,CYP707A1,OST1,CYP707A3* | 1.72E-05 | 0.0011 | Monocarboxylic acid metabolic process |
| Process | 26 | *CDPK2,BGLU18,RPK1,SNRK2-8,TPS1,AAO3,PKT3,ELO1,NCED3,AT3G58490,CPK21,CPK23,AT4G16720,CIPK12,CYP707A1,ABI1,OST1,AT4G35910,HD1,CIPK15,CBP20,CYP707A3,RBOHD,ABI2,HDA6,YCF3* | 1.73E-05 | 0.0011 | Cellular metabolic process |
| Process | 8 | *BGLU18,AAO3,PKT3,NCED3,AT3G58490,CYP707A1,OST1,CYP707A3* | 1.69E-05 | 0.0011 | Cellular lipid metabolic process |
| Process | 6 | *BGLU18,XF1,AAO3,NCED3,CYP707A1,CYP707A3* | 1.67E-05 | 0.0011 | Organic hydroxy compound metabolic process |
| Process | 4 | *PKT3,NCED3,CYP707A1,CYP707A3* | 2.42E-05 | 0.0015 | Cellular lipid catabolic process |
| Process | 9 | *BGLU18,XF1,AAO3,PKT3,NCED3,AT3G58490,CYP707A1,OST1,CYP707A3* | 2.56E-05 | 0.0016 | Lipid metabolic process |
| Process | 5 | *MYB60,ABI1,CIPK15,EIN2,ABI2* | 3.22E-05 | 0.002 | Negative regulation of response to stimulus |
| Process | 2 | *HD1,HDA6* | 4.66E-05 | 0.0028 | Histone h4 deacetylation |
| Process | 2 | *CYP707A1,CYP707A3* | 6.51E-05 | 0.0039 | Abscisic acid catabolic process |
| Process | 13 | *CDPK2,RPK1,SNRK2-8,CPK21,CPK23,CIPK12,ABI1,OST1,AT4G35910,HD1,CIPK15,ABI2,HDA6* | 7.46E-05 | 0.0043 | Cellular protein modification process |
| Process | 14 | *CDPK2,RPK1,SNRK2-8,ELO1,CPK21,CPK23,CIPK12,ABI1,OST1,AT4G35910,HD1,CIPK15,ABI2,HDA6* | 7.75E-05 | 0.0043 | Macromolecule modification |
| Process | 4 | *MYB60,KAT2,OST1,ABI2* | 0.00011 | 0.0057 | Response to light intensity |
| Process | 3 | *ABI1,CIPK15,ABI2* | 1.00E-04 | 0.0057 | Negative regulation of abscisic acid-activated signaling pathway |
| Process | 3 | *CDPK2,KAT2,OST1* | 1.00E-04 | 0.0057 | Regulation of ion transmembrane transport |
| Process | 3 | *TPS1,ELO1,EIN2* | 0.00012 | 0.0063 | Cellular response to carbohydrate stimulus |
| Process | 4 | *MYB60,TPS1,ELO1,EIN2* | 0.00014 | 0.007 | Response to carbohydrate |
| Process | 11 | *CDPK2,RPK1,SNRK2-8,AT3G58490,CPK21,CPK23,CIPK12,ABI1,OST1,CIPK15,ABI2* | 0.00016 | 0.0078 | Phosphate-containing compound metabolic process |
| Process | 2 | *RPK1,WRKY2* | 0.00017 | 0.0084 | Longitudinal axis specification |
| Process | 3 | *PKT3,CYP707A1,CYP707A3* | 0.00017 | 0.0084 | Monocarboxylic acid catabolic process |
| Process | 4 | *CDPK2,CPK21,CPK23,OST1* | 2.00E-04 | 0.0094 | Protein autophosphorylation |
| Process | 3 | *XF1,CYP707A1,CYP707A3* | 0.00025 | 0.0116 | Sterol metabolic process |
| Process | 2 | *HD1,EIN2* | 0.00028 | 0.013 | Jasmonic acid and ethylene-dependent systemic resistance |
| Process | 14 | *CDPK2,RPK1,SNRK2-8,CPK21,CPK23,AT4G16720,CIPK12,ABI1,OST1,AT4G35910,HD1,CIPK15,ABI2,HDA6* | 3.00E-04 | 0.0137 | Cellular protein metabolic process |
| Process | 2 | *ELO1,AT4G35910* | 0.00032 | 0.0147 | tRNA wobble uridine modification |
| Process | 2 | *CDPK2,OST1* | 0.00032 | 0.0147 | Regulation of ion transmembrane transporter activity |
| Process | 4 | *PKT3,HD1,EIN2,RBOHD* | 0.00034 | 0.0148 | Response to wounding |
| Process | 2 | *OST1,ABI2* | 0.00037 | 0.0159 | Regulation of stomatal opening |
| Process | 3 | *CDPK2,CPK21,CPK23* | 4.00E-04 | 0.017 | Peptidyl-serine phosphorylation |
| Process | 2 | *AAO3,NCED3* | 0.00047 | 0.0192 | Abscisic acid biosynthetic process |
| Process | 6 | *CDPK2,SNRK2-8,CPK21,CPK23,OST1,EIN2* | 0.00053 | 0.0209 | Intracellular signal transduction |
| Process | 4 | *AAO3,PKT3,NCED3,OST1* | 0.00068 | 0.0268 | Monocarboxylic acid biosynthetic process |
| Process | 2 | *GolS2,GolS1* | 0.00084 | 0.0318 | Galactose metabolic process |
| Process | 9 | *BGLU18,GolS2,AAO3,PKT3,GolS1,NCED3,CYP707A1,OST1,CYP707A3* | 0.0013 | 0.046 | Small molecule metabolic process |
| Process | 13 | *AGL28,MYB60,bZIP23,ELO1,CBF2,CBF1,OST1,HD1,CBP20,WRKY2,ABI2,HDA6,MYBR1* | 0.0013 | 0.0464 | Regulation of metabolic process |
| Process | 16 | *CDPK2,BGLU18,RPK1,SNRK2-8,AT3G58490,CPK21,CPK23,AT4G16720,CIPK12,ABI1,OST1,AT4G35910,HD1,CIPK15,ABI2,HDA6* | 0.0013 | 0.0464 | Organonitrogen compound metabolic process |

| **Supplementary Table 10.** Genes significantly enriched in different molecular Function using shinyGO and using Arabidopsis model | | | | | |
| --- | --- | --- | --- | --- | --- |
| **category** | **Number of genes** | **Gene Names** | **p_value** | **FDR** | **description** |
| Function | 37 | *AGL28,MYB60,CDPK2,BGLU18,GolS2,XF1,ZCF125,RPK1,ABCG25,SNRK2-8, bZIP23,AAO3,CYP71A12,GolS1,SPD1, NCED3, CPK21, CPK23,AT4G16720, CIPK12,CYP707A1, CBF2,CBF1, ABI1,OST1,AT4G35910,CIPK15,EIN2,ABCG22,CBP20,CYP707A3,RBOHD,ERD1,WRKY2,ABI2,ABCA10,MYBR1* | 1.67E-10 | 3.50E-07 | Binding |
| Function | 30 | *AGL28,MYB60,CDPK2,XF1,ZCF125,RPK1,ABCG25,SNRK2-8,bZIP23,AAO3,CYP71A12,SPD1,CPK21,CPK23,AT4G16720,CIPK12,CYP707A1,CBF2,CBF1,OST1,AT4G35910,CIPK15,EIN2,ABCG22,CBP20,CYP707A3,ERD1,WRKY2,ABCA10,MYBR1* | 2.88E-10 | 3.50E-07 | Organic cyclic compound binding |
| Function | 30 | *AGL28,MYB60,CDPK2,XF1,ZCF125,RPK1,ABCG25,SNRK2-8,bZIP23,AAO3,CYP71A12,SPD1,CPK21,CPK23,AT4G16720,CIPK12,CYP707A1,CBF2,CBF1,OST1,AT4G35910,CIPK15,EIN2,ABCG22,CBP20,CYP707A3,ERD1,WRKY2,ABCA10,MYBR1* | 2.61E-10 | 3.50E-07 | Heterocyclic compound binding |
| Function | 32 | *CDPK2,BGLU18,GolS2,XF1,ZCF125,RPK1,ABCG25,SNRK2-8,TPS1,AAO3,CYP71A12,PKT3,GolS1,ELO1,NCED3,AT3G58490,CPK21,CPK23,CIPK12,CYP707A1,ABI1,OST1,AT4G35910,HD1,CIPK15,ABCG22,CYP707A3,RBOHD,ERD1,ABI2,ABCA10,HDA6* | 2.01E-09 | 1.06E-06 | Catalytic activity |
| Function | 26 | *CDPK2,GolS2,XF1,ZCF125,RPK1,ABCG25,SNRK2-8, AAO3, CYP71A12, GolS1,SPD1,NCED3,CPK21, CPK23,CIPK12, CYP707A1,ABI1,OST1,CIPK15,ABCG22,CYP707A3,RBOHD,ERD1, WRKY2,ABI2,ABCA10* | 9.50E-08 | 3.99E-05 | Ion binding |
| Function | 3 | *CPK21,CPK23,OST1* | 1.51E-06 | 0.00053 | Protein phosphatase binding |
| Function | 4 | *CDPK2,CPK21,CPK23,OST1* | 1.94E-06 | 0.00058 | Calcium-dependent protein serine/ threonine kinase activity |
| Function | 16 | *CDPK2,XF1,ZCF125,RPK1,ABCG25,SNRK2-8,AAO3,SPD1,CPK21,CPK23,CIPK12,OST1,CIPK15,ABCG22,ERD1,ABCA10* | 4.13E-06 | 0.00087 | Nucleotide binding |
| Function | 14 | *CDPK2,ZCF125,RPK1,ABCG25,SNRK2-8,SPD1,CPK21,CPK23,CIPK12,OST1,CIPK15,ABCG22,ERD1,ABCA10* | 5.90E-06 | 0.001 | ATP binding |
| Function | 16 | *CDPK2,XF1,ZCF125,RPK1,ABCG25,SNRK2-8,AAO3,SPD1,CPK21,CPK23,CIPK12,OST1,CIPK15,ABCG22,ERD1,ABCA10* | 1.02E-05 | 0.0014 | Anion binding |
| Function | 8 | *CDPK2,RPK1,SNRK2-8,CPK21,CPK23,CIPK12,OST1,CIPK15* | 3.97E-05 | 0.0038 | Protein serine/threonine kinase activity |
| Function | 2 | *CYP707A1,CYP707A3* | 4.66E-05 | 0.0043 | (+)-abscisic acid 8-hydroxylase activity |
| Function | 12 | *CDPK2,RPK1,SNRK2-8,CPK21,CPK23,CIPK12,ABI1,OST1,HD1,CIPK15,ABI2,HDA6* | 1.00E-04 | 0.0085 | Catalytic activity, acting on a protein |
| Function | 2 | *GolS2,GolS1* | 0.00014 | 0.0112 | Inositol 3-alpha-galactosyltransferase activity |
| Function | 9 | *CDPK2,RPK1,SNRK2-8,CPK21,CPK23,CIPK12,OST1,AT4G35910,CIPK15* | 0.00017 | 0.0129 | Transferase activity, transferring phosphorus-containing groups |
| Function | 14 | *CDPK2,GolS2,RPK1,SNRK2-8,TPS1,PKT3,GolS1,ELO1,CPK21,CPK23,CIPK12,OST1,AT4G35910,CIPK15* | 0.00025 | 0.0183 | Transferase activity |
| Function | 2 | *HD1,HDA6* | 0.00028 | 0.0196 | NAD-dependent histone deacetylase activity (H3-K14 specific) |
| Function | 5 | *AAO3,CPK21,CPK23,ABI1,OST1* | 4.00E-04 | 0.0239 | Enzyme binding |
| Function | 4 | *XF1,CYP71A12,CYP707A1,CYP707A3* | 0.00055 | 0.0295 | Monooxygenase activity |
| Function | 14 | *CDPK2,GolS2,AAO3,CYP71A12,GolS1,NCED3,CPK21,CPK23,CYP707A1,ABI1,CYP707A3,RBOHD,WRKY2,ABI2* | 0.00063 | 0.033 | Metal ion binding |
| Function | 4 | *AAO3,CYP71A12,CYP707A1,CYP707A3* | 0.00074 | 0.0363 | Iron ion binding |
| Function | 4 | *CDPK2,CPK21,CPK23,RBOHD* | 9E-04 | 0.0429 | Calcium ion binding |

| Supplementary Table 11. Cluster of genes using gene set enrichment score in several molecular function | |
| --- | --- |
| Gene Group 1 | Enrichment Score: 1.33 |
| ENTREZ_GENE_ID | Gene Name |
| 4326871 | ocs element-binding factor 1 |
| 4324418 | dehydration-responsive element-binding protein 2A-like |
| 4324824 | WUSCHEL-related homeobox 9-like |
| 4330838 | bZIP transcription factor 23-like |
| 4334553 | NAC domain-containing protein 2-like |
| 4347620 | dehydration-responsive element-binding protein 1A-like |
| 4339974 | dehydration-responsive element-binding protein 1C |
| 4344714 | AT-hook motif nuclear-localized protein 23) |
| Gene Group 2 | Enrichment Score: 0.78 |
| ENTREZ_GENE_ID | Gene Name |
| 4330248 | aquaporin PIP1-1-like |
| 4340585 | RING-H2 finger protein ATL46 |
| 4340300 | protein NUCLEAR FUSION DEFECTIVE 4 |
| 4330049 | probable aquaporin PIP2-2) |
| 4332352 | uncharacterized LOC4332352 |
| 4350916 | uncharacterized LOC4350916 |
| 4342173 | potassium transporter 22-like |
| 4336249 | protein NRT1/ PTR FAMILY 4.5 |
| 4329854 | beta-1,4-mannosyl-glycoprotein 4-beta-N-acetylglucosaminyltransferase |
| 4340325 | uncharacterized |
| 4339571 | probable purine permease 4 |
| 4337170 | probable pectinesterase/pectinesterase inhibitor 13 |
| 4333878 | uncharacterized |
| 4345581 | protein CHAPERONE-LIKE PROTEIN OF POR1, chloroplastic |
| 4333501 | protein DETOXIFICATION 29 |
| 4342431 | protein ETHYLENE-INSENSITIVE 2-like |
| 107278728 | rust resistance kinase Lr10 |
| 4333169 | uncharacterized |
| 4331194 | aquaporin PIP 1-3-like |
| 4335799 | photosystem I subunit O |
| 4338289 | probable glycerol-3-phosphate acyltransferase 3 |

| **Supplementary Table 12. Cluster of genes having critical function in several molecular function using Kappa score** | | | | | |
| --- | --- | --- | --- | --- | --- |
| **Cluster 1** | **SN** | **Gene** | **Species** | **Kappa** | **Kappa value term** |
| 1 | 1 | dehydration-responsive element-binding protein 1C (LOC4339974) | *Oryza sativa Japonica Group* | 0.83 | very High |
|  | 2 | dehydration-responsive element-binding protein 1A-like(LOC4347620) | *Oryza sativa Japonica Group* | 0.80 |  |
|  | 3 | dehydration-responsive element-binding protein 2A-like(LOC4324418) | *Oryza sativa Japonica Group* | 0.695 | High |
|  | 4 | C-repeat/DRE binding factor 2(CBF2) | *Arabidopsis thaliana* | 0.65 |  |
|  | 5 | NAC domain-containing protein 2-like(LOC4334553) | *Oryza sativa Japonica Group* | 0.64 |  |
|  | 6 | AT-hook motif nuclear-localized protein 23(LOC4344714) | *Oryza sativa Japonica Group* | 0.64 |  |
|  | 7 | light-inducible protein CPRF2(LOC8061169) | *Sorghum bicolor* | 0.60 |  |
|  | 8 | C-repeat/DRE binding factor 1(CBF1) | *Arabidopsis thaliana* | 0.59 |  |
|  | 9 | bZIP transcription factor 23-like(LOC4330838) | *Oryza sativa Japonica Group* | 0.59 |  |
|  | 10 | WUSCHEL-related homeobox 9-like(LOC4324824) | *Oryza sativa Japonica Group* | 0.59 |  |
|  | 11 | cyclic dof factor 2(LOC8078579) | *Sorghum bicolor* | 0.52 |  |
|  | 12 | cyclic dof factor 1(LOC8082122) | *Sorghum bicolor* | 0.52 |  |
|  | 13 | bZIP transcription factor 68-like(LOC110429775) | *Sorghum bicolor* | 0.50 |  |
|  | 14 | ocs element-binding factor 1(LOC4326871) | *Oryza sativa Japonica Group* | 0.50 |  |
|  | 15 | DRE-binding protein 2A(DREB2A) | *Arabidopsis thaliana* | 0.4877792 | Moderate |
|  | 16 | homeodomain GLABROUS 11(HDG11) | *Arabidopsis thaliana* | 0.4444178 |  |
|  | 17 | ETHYLENE INSENSITIVE 3-like 1 protein(LOC8083691) | *Sorghum bicolor* | 0.4210344 |  |
|  | 18 | auxin response factor 22(LOC8067406) | *Sorghum bicolor* | 0.4166441 |  |
|  | 19 | CAAT-box DNA binding protein subunit B (NF-YB)(LOC542390) | *Zea mays* | 0.4137676 |  |
|  | 20 | probable inactive DNA (cytosine-5)-methyltransferase DRM3(LOC4337721) | *Oryza sativa Japonica Group* | 0.3999761 |  |
|  | 21 | histone H3.2(LOC103641629) | *Zea mays* | 0.3571154 |  |
| **Cluster II** | **SN** | **Gene** | **Species** | **Kappa** |  |
|  | 1 | uncharacterized LOC4333169(LOC4333169) | *Oryza sativa Japonica Group* | 0.9230753 | Very high |
|  | 2 | uncharacterized LOC103643967(LOC103643967) | *Zea mays* | 0.9230753 |  |
|  | 3 | uncharacterized LOC4340325(LOC4340325) | *Oryza sativa Japonica Group* | 0.9090893 |  |
|  | 4 | uncharacterized LOC4333878(LOC4333878) | *Oryza sativa Japonica Group* | 0.8571396 |  |
|  | 5 | uncharacterized LOC4332352(LOC4332352) | *Oryza sativa Japonica Group* | 0.83 |  |
|  | 6 | uncharacterized LOC8075414(LOC8075414) | *Sorghum bicolor* | 0.78 |  |
|  | 7 | probable purine permease 4(LOC4339571) | *Oryza sativa Japonica Group* | 0.78 |  |
|  | 8 | protein NRT1/ PTR FAMILY 4.5(LOC4336249) | *Oryza sativa Japonica Group* | 0.76 |  |
|  | 9 | uncharacterized LOC4350916(LOC4350916) | *Oryza sativa Japonica Group* | 0.7272678 | High |
|  | 10 | tonoplast dicarboxylate transporter(LOC8055864) | *Sorghum bicolor* | 0.7142792 |  |
|  | 11 | protein CHAPERONE-LIKE PROTEIN OF POR1, chloroplastic(LOC4345581) | *Oryza sativa Japonica Group* | 0.7142792 |  |
|  | 12 | photosystem I subunit O(LOC4335799) | *Oryza sativa Japonica Group* | 0.7142792 |  |
|  | 13 | 5'-adenylylsulfate reductase-like 5(LOC8081214) | *Sorghum bicolor* | 0.7142792 |  |
|  | 14 | protein NUCLEAR FUSION DEFECTIVE 4(LOC4340300) | *Oryza sativa Japonica Group* | 0.7058748 |  |
|  | 15 | uncharacterized LOC100273814(LOC100273814) | *Zea mays* | 0.6249907 |  |
|  | 16 | rust resistance kinase Lr10(LOC107278728) | *Oryza sativa Japonica Group* | 0.6249907 |  |
|  | 17 | protein DETOXIFICATION 29(LOC4333501) | *Oryza sativa Japonica Group* | 0.6249907 |  |
|  | 18 | uncharacterized LOC8060854(LOC8060854) | *Sorghum bicolor* | 0.6153764 |  |
|  | 19 | beta-1,4-mannosyl-glycoprotein 4-beta-N-acetylglucosaminyltransferase(LOC4329854) | *Oryza sativa Japonica Group* | 0.5882247 |  |
|  | 20 | RING-H2 finger protein ATL46(LOC4340585) | *Oryza sativa Japonica Group* | 0.5714164 |  |
|  | 21 | UDP-glucuronate 4-epimerase 1(LOC100274751) | *Zea mays* | 0.521725 |  |
|  | 22 | probable pectinesterase/pectinesterase inhibitor 13(LOC4337170) | *Oryza sativa Japonica Group* | 0.461522 | Moderate |
|  | 23 | protein ETHYLENE-INSENSITIVE 2-like(LOC4342431) | *Oryza sativa Japonica Group* | 0.461522 |  |
|  | 24 | protein PIN-LIKES 2(LOC8072447) | *Sorghum bicolor* | 0.4444297 |  |
|  | 25 | potassium transporter 10(LOC100281081) | *Zea mays* | 0.4444297 |  |
|  | 26 | aquaporin PIP1-1-like(LOC4330248) | *Oryza sativa Japonica Group* | 0.4137746 |  |
|  | 27 | ABC transporter G family member 11(LOC8076352) | *Sorghum bicolor* | 0.3999833 |  |
|  | 28 | probable glycerol-3-phosphate acyltransferase 3(LOC4338289) | *Oryza sativa Japonica Group* | 0.3999818 |  |
|  | 29 | probable ADP,ATP carrier protein At5g56450(LOC8061071) | *Sorghum bicolor* | 0.3809348 |  |
|  | 30 | ABC-2 type transporter family protein(ABCG22) | *Arabidopsis thaliana* | 0.3749798 |  |
|  | 31 | probable aquaporin PIP2-2(LOC4330049) | *Oryza sativa Japonica Group* | 0.3703509 |  |
|  | 32 | aquaporin PIP 1-3-like(LOC4331194) | *Oryza sativa Japonica Group* | 0.3571227 |  |

| **Supplementary Table 13 A**. Genes involved in several biological pathways using KEGG pathway enrichment analysis | | | | |
| --- | --- | --- | --- | --- |
|  | ID | Gene Name | Species | KEGG_PATHWAY |
| 1 | 826730 | 1-aminocyclopropane-1-carboxylic acid (acc) synthase 6(ACS6) | *Arabidopsis thaliana* | ath00270:Cysteine and methionine metabolism,ath01100:Metabolic pathways,ath01110:Biosynthesis of secondary metabolites,ath04016:MAPK signaling pathway - plant, |
| 2 | 8073342 | 16.9 kDa class I heat shock protein 1(LOC8073342) | *Sorghum bicolor* | sbi04141:Protein processing in endoplasmic reticulum, |
| 3 | 8058459 | 4-hydroxy-tetrahydrodipicolinate synthase, chloroplastic(LOC8058459) | *Sorghum bicolor* | sbi00261:Monobactam biosynthesis,sbi00300:Lysine biosynthesis,sbi01100:Metabolic pathways,sbi01110:Biosynthesis of secondary metabolites,sbi01230:Biosynthesis of amino acids, |
| 4 | 4337526 | 50S ribosomal protein L28, chloroplastic(LOC4337526) | *Oryza sativa Japonica Group* | osa03010:Ribosome, |
| 5 | 8062999 | 60S ribosomal protein L10(LOC8062999) | *Sorghum bicolor* | sbi03010:Ribosome, |
| 6 | 103642077 | 60S ribosomal protein L27-3(LOC103642077) | *Zea mays* | zma03010:Ribosome, |
| 7 | 100193672 | 60S ribosomal protein L35a-like(LOC100193672) | *Zea mays* | zma03010:Ribosome, |
| 8 | 830541 | ABC-2 type transporter family protein(ABCG22) | *Arabidopsis thaliana* | ath02010:ABC transporters, |
| 9 | 843527 | ATP-binding casette family G25(ABCG25) | *Arabidopsis thaliana* | ath02010:ABC transporters, |
| 10 | 840158 | Basic helix-loop-helix (bHLH) DNA-binding family protein(MYC2) | *Arabidopsis thaliana* | ath04016:MAPK signaling pathway - plant,ath04075:Plant hormone signal transduction, |
| 11 | 834443 | CAP-binding protein 20(CBP20) | *Arabidopsis thaliana* | ath03013:Nucleocytoplasmic transport,ath03015:mRNA surveillance pathway,ath03040:Spliceosome, |
| 12 | 831241 | Chalcone and stilbene synthase family protein(TT4) | *Arabidopsis thaliana* | ath00941:Flavonoid biosynthesis,ath00960:Tropane, piperidine and pyridine alkaloid biosynthesis,ath01100:Metabolic pathways,ath01110:Biosynthesis of secondary metabolites,ath04712:Circadian rhythm - plant, |
| 13 | 8066485 | DExH-box ATP-dependent RNA helicase DExH12(LOC8066485) | *Sorghum bicolor* | sbi03040:Spliceosome, |
| 14 | 4344172 | E3 ubiquitin-protein ligase DIS1-like(LOC4344172) | *Oryza sativa Japonica Group* | osa04120:Ubiquitin mediated proteolysis, |
| 15 | 8083691 | ETHYLENE INSENSITIVE 3-like 1 protein(LOC8083691) | *Sorghum bicolor* | sbi04016:MAPK signaling pathway - plant,sbi04075:Plant hormone signal transduction, |
| 16 | 842213 | FAD/NAD(P)-binding oxidoreductase family protein(XF1) | *Arabidopsis thaliana* | ath00100:Steroid biosynthesis,ath00909:Sesquiterpenoid and triterpenoid biosynthesis,ath01100:Metabolic pathways,ath01110:Biosynthesis of secondary metabolites, |
| 17 | 837576 | Glutathione S-transferase family protein(ERD9) | *Arabidopsis thaliana* | ath00480:Glutathione metabolism,ath01100:Metabolic pathways, |
| 18 | 816413 | H[+]-ATPase 1(HA1) | *Arabidopsis thaliana* | ath00190:Oxidative phosphorylation,ath01100:Metabolic pathways, |
| 19 | 4326769 | NADP-dependent malic enzyme, chloroplastic-like(LOC4326769) | *Oryza sativa Japonica Group* | osa00620:Pyruvate metabolism,osa00710:Carbon fixation in photosynthetic organisms,osa01100:Metabolic pathways,osa01200:Carbon metabolism, |
| 20 | 831889 | NRAMP metal ion transporter family protein(EIN2) | *Arabidopsis thaliana* | ath04016:MAPK signaling pathway - plant,ath04075:Plant hormone signal transduction, |
| 21 | 841514 | Nucleolar GTP-binding protein(AT1G50920) | *Arabidopsis thaliana* | ath03008:Ribosome biogenesis in eukaryotes, |
| 22 | 829541 | Protein kinase superfamily protein(OST1) | *Arabidopsis thaliana* | ath04016:MAPK signaling pathway - plant,ath04075:Plant hormone signal transduction, |
| 23 | 844164 | Protein kinase superfamily protein(SNRK2-8) | *Arabidopsis thaliana* | ath04016:MAPK signaling pathway - plant,ath04075:Plant hormone signal transduction, |
| 24 | 828714 | Protein phosphatase 2C family protein(ABI1) | *Arabidopsis thaliana* | ath04016:MAPK signaling pathway - plant,ath04075:Plant hormone signal transduction, |
| 25 | 835809 | Protein phosphatase 2C family protein(ABI2) | *Arabidopsis thaliana* | ath04016:MAPK signaling pathway - plant,ath04075:Plant hormone signal transduction, |
| 26 | 4329918 | TNF receptor-associated factor 6(LOC4329918) | *Oryza sativa Japonica Group* | osa00130:Ubiquinone and other terpenoid-quinone biosynthesis,osa01100:Metabolic pathways,osa01110:Biosynthesis of secondary metabolites,osa01240:Biosynthesis of cofactors, |
| 27 | 110436990 | UDP-glucose 6-dehydrogenase 5(LOC110436990) | *Sorghum bicolor* | sbi00040:Pentose and glucuronate interconversions,sbi00053:Ascorbate and aldarate metabolism,sbi00520:Amino sugar and nucleotide sugar metabolism,sbi01100:Metabolic pathways,sbi01240:Biosynthesis of cofactors,sbi01250:Biosynthesis of nucleotide sugars, |
| 28 | 100274751 | UDP-glucuronate 4-epimerase 1(LOC100274751) | *Zea mays* | zma00053:Ascorbate and aldarate metabolism,zma00520:Amino sugar and nucleotide sugar metabolism,zma01100:Metabolic pathways,zma01240:Biosynthesis of cofactors,zma01250:Biosynthesis of nucleotide sugars, |
| 29 | 835726 | WRKY DNA-binding protein 2(WRKY2) | *Arabidopsis thaliana* | ath04626:Plant-pathogen interaction, |
| 30 | 817257 | abscisic aldehyde oxidase 3(AAO3) | *Arabidopsis thaliana* | ath00906:Carotenoid biosynthesis,ath01100:Metabolic pathways,ath01110:Biosynthesis of secondary metabolites, |
| 31 | 820121 | ascorbate peroxidase 2(APX2) | *Arabidopsis thaliana* | ath00053:Ascorbate and aldarate metabolism,ath00480:Glutathione metabolism,ath01100:Metabolic pathways, |
| 32 | 4349090 | auxin-responsive protein SAUR32(LOC4349090) | *Oryza sativa Japonica Group* | osa04075:Plant hormone signal transduction, |
| 33 | 4330838 | bZIP transcription factor 23-like(LOC4330838) | *Oryza sativa Japonica Group* | osa04075:Plant hormone signal transduction, |
| 34 | 841670 | beta glucosidase 18(BGLU18) | *Arabidopsis thaliana* | ath00906:Carotenoid biosynthesis,ath01110:Biosynthesis of secondary metabolites, |
| 35 | 4329854 | beta-1,4-mannosyl-glycoprotein 4-beta-N-acetylglucosaminyltransferase(LOC4329854) | *Oryza sativa Japonica Group* | osa00510:N-Glycan biosynthesis,osa01100:Metabolic pathways, |
| 36 | 840471 | calcium-dependent protein kinase 2(CDPK2) | *Arabidopsis thaliana* | ath04626:Plant-pathogen interaction, |
| 37 | 825807 | calcium-dependent protein kinase 21(CPK21) | *Arabidopsis thaliana* | ath04626:Plant-pathogen interaction, |
| 38 | 4346187 | calcium-dependent protein kinase 21-like(LOC4346187) | *Oryza sativa Japonica Group* | osa04626:Plant-pathogen interaction, |
| 39 | 825809 | calcium-dependent protein kinase 23(CPK23) | *Arabidopsis thaliana* | ath04626:Plant-pathogen interaction, |
| 40 | 8083705 | calmodulin(LOC8083705) | *Sorghum bicolor* | sbi04016:MAPK signaling pathway - plant,sbi04070:Phosphatidylinositol signaling system,sbi04626:Plant-pathogen interaction, |
| 41 | 8058923 | coronatine-insensitive protein homolog 1a(LOC8058923) | *Sorghum bicolor* | sbi04075:Plant hormone signal transduction, |
| 42 | 839226 | cullin 3(CUL3) | *Arabidopsis thaliana* | ath04120:Ubiquitin mediated proteolysis, |
| 43 | 827663 | cytochrome P450, family 707, subfamily A, polypeptide 1(CYP707A1) | *Arabidopsis thaliana* | ath00906:Carotenoid biosynthesis,ath01110:Biosynthesis of secondary metabolites, |
| 44 | 834570 | cytochrome P450, family 707, subfamily A, polypeptide 3(CYP707A3) | *Arabidopsis thaliana* | ath00906:Carotenoid biosynthesis,ath01110:Biosynthesis of secondary metabolites, |
| 45 | 4343863 | dehydrodolichyl diphosphate synthase 6(LOC4343863) | *Oryza sativa Japonica Group* | osa00900:Terpenoid backbone biosynthesis,osa01110:Biosynthesis of secondary metabolites, |
| 46 | 8068630 | disease resistance protein RPS2(LOC8068630) | *Sorghum bicolor* | sbi04626:Plant-pathogen interaction, |
| 47 | 4331811 | elongation factor 1-alpha-like(LOC4331811) | *Oryza sativa Japonica Group* | osa03013:Nucleocytoplasmic transport, |
| 48 | 4332538 | eukaryotic translation initiation factor 2 subunit alpha homolog(LOC4332538) | *Oryza sativa Japonica Group* | osa04141:Protein processing in endoplasmic reticulum, |
| 49 | 832311 | fatty acid reductase 1(FAR1) | *Arabidopsis thaliana* | ath00073:Cutin, suberine and wax biosynthesis,ath04146:Peroxisome, |
| 50 | 819331 | galactinol synthase 1(GolS1) | *Arabidopsis thaliana* | ath00052:Galactose metabolism,ath01100:Metabolic pathways, |
| 51 | 842114 | galactinol synthase 2(GolS2) | *Arabidopsis thaliana* | ath00052:Galactose metabolism,ath01100:Metabolic pathways, |
| 52 | 4347311 | glucose-6-phosphate isomerase 1, chloroplastic(LOC4347311) | *Oryza sativa Japonica Group* | osa00010:Glycolysis / Gluconeogenesis,osa00030:Pentose phosphate pathway,osa00500:Starch and sucrose metabolism,osa00520:Amino sugar and nucleotide sugar metabolism,osa01100:Metabolic pathways,osa01110:Biosynthesis of secondary metabolites,osa01200:Carbon metabolism,osa01250:Biosynthesis of nucleotide sugars, |
| 53 | 818936 | glutathione peroxidase 3(GPX3) | *Arabidopsis thaliana* | ath00480:Glutathione metabolism,ath00590:Arachidonic acid metabolism,ath01100:Metabolic pathways, |
| 54 | 8057776 | heat shock 70 kDa protein(LOC8057776) | *Sorghum bicolor* | sbi03040:Spliceosome,sbi04141:Protein processing in endoplasmic reticulum,sbi04144:Endocytosis, |
| 55 | 8055854 | heat shock protein 81-2(LOC8055854) | *Sorghum bicolor* | sbi04141:Protein processing in endoplasmic reticulum,sbi04626:Plant-pathogen interaction, |
| 56 | 4345657 | malate dehydrogenase, chloroplastic(LOC4345657) | *Oryza sativa Japonica Group* | osa00020:Citrate cycle (TCA cycle),osa00270:Cysteine and methionine metabolism,osa00620:Pyruvate metabolism,osa00630:Glyoxylate and dicarboxylate metabolism,osa00710:Carbon fixation in photosynthetic organisms,osa01100:Metabolic pathways,osa01110:Biosynthesis of secondary metabolites,osa01200:Carbon metabolism, |
| 57 | 820667 | nine-cis-epoxycarotenoid dioxygenase 3(NCED3) | *Arabidopsis thaliana* | ath00906:Carotenoid biosynthesis,ath01100:Metabolic pathways,ath01110:Biosynthesis of secondary metabolites, |
| 58 | 817876 | peroxisomal 3-ketoacyl-CoA thiolase 3(PKT3) | *Arabidopsis thaliana* | ath00071:Fatty acid degradation,ath00280:Valine, leucine and isoleucine degradation,ath00592:alpha-Linolenic acid metabolism,ath01040:Biosynthesis of unsaturated fatty acids,ath01100:Metabolic pathways,ath01110:Biosynthesis of secondary metabolites,ath01212:Fatty acid metabolism,ath04146:Peroxisome, |
| 59 | 4335799 | photosystem I subunit O(LOC4335799) | *Oryza sativa Japonica Group* | osa00195:Photosynthesis,osa01100:Metabolic pathways, |
| 60 | 8057594 | polyphenol oxidase I, chloroplastic(LOC8057594) | *Sorghum bicolor* | sbi00350:Tyrosine metabolism,sbi00950:Isoquinoline alkaloid biosynthesis,sbi01100:Metabolic pathways,sbi01110:Biosynthesis of secondary metabolites, |
| 61 | 8082723 | probable alpha,alpha-trehalose-phosphate synthase [UDP-forming] 11(LOC8082723) | *Sorghum bicolor* | sbi00500:Starch and sucrose metabolism,sbi01100:Metabolic pathways,sbi01110:Biosynthesis of secondary metabolites, |
| 62 | 4332731 | probable calcium-binding protein CML27(LOC4332731) | *Oryza sativa Japonica Group* | osa04626:Plant-pathogen interaction, |
| 63 | 4338289 | probable glycerol-3-phosphate acyltransferase 3(LOC4338289) | *Oryza sativa Japonica Group* | osa00561:Glycerolipid metabolism,osa00564:Glycerophospholipid metabolism,osa01100:Metabolic pathways,osa01110:Biosynthesis of secondary metabolites, |
| 64 | 8078643 | probable protein phosphatase 2C 6(LOC8078643) | *Sorghum bicolor* | sbi04016:MAPK signaling pathway - plant,sbi04075:Plant hormone signal transduction, |
| 65 | 4342431 | protein ETHYLENE-INSENSITIVE 2-like(LOC4342431) | *Oryza sativa Japonica Group* | osa04016:MAPK signaling pathway - plant,osa04075:Plant hormone signal transduction, |
| 66 | 8064218 | putative linoleate 9S-lipoxygenase 3(LOC8064218) | *Sorghum bicolor* | sbi00591:Linoleic acid metabolism, |
| 67 | 838452 | regulatory component of ABA receptor 1(RCAR1) | *Arabidopsis thaliana* | ath04016:MAPK signaling pathway - plant,ath04075:Plant hormone signal transduction, |
| 68 | 834842 | respiratory burst oxidase homologue D(RBOHD) | *Arabidopsis thaliana* | ath04016:MAPK signaling pathway - plant,ath04626:Plant-pathogen interaction, |
| 69 | 842710 | respiratory burst oxidase protein F(RBOH F) | *Arabidopsis thaliana* | ath04016:MAPK signaling pathway - plant,ath04626:Plant-pathogen interaction, |
| 70 | 844194 | trehalose-6-phosphate synthase(TPS1) | *Arabidopsis thaliana* | ath00500:Starch and sucrose metabolism,ath01100:Metabolic pathways,ath01110:Biosynthesis of secondary metabolites, |
| 71 | 819323 | ubiquitin 6(UBQ6) | *Arabidopsis thaliana* | ath03010:Ribosome,ath04120:Ubiquitin mediated proteolysis, |
| 72 | 100283330 | uncharacterized LOC100283330(LOC100283330) | *Zea mays* | zma00190:Oxidative phosphorylation,zma01100:Metabolic pathways,zma04145:Phagosome, |

| **Supplementary Table 13 B. Pathway analysis using KEGG with ShinyGO** | | | | |
| --- | --- | --- | --- | --- |
| Enrichment FDR | nGenes | Fold Enrichment | Pathway | Genes |
| 5.26E-06 | 10 | 8.862 | DNA-binding transcription factor activity | *MYB60 MYC2 HDG11 bZIP23 FHY3 FAR1 CBF2 CBF1 WRKY2 MYBR1* |
| 1.65E-05 | 6 | 17.68 | Protein kinase activity | *CDPK2 RPK1 CPK21 CPK23 OST1 CIPK15* |
| 5.51E-05 | 6 | 13.51 | Protein serine/threonine kinase activity | *CDPK2 RPK1 SNRK2-8 CIPK12 OST1 CIPK15* |
| 9.32E-05 | 6 | 11.71 | Transcription cis-regulatory region binding | *MYB60 MYC2 HDG11 CBF2 WRKY2 MYBR1* |
| 8.71E-06 | 5 | 34.54 | Identical protein binding | *BGLU18 RPK1 ACS6 OST1 MYBR1* |
| 1.65E-05 | 5 | 27.45 | Calcium ion binding | *CDPK2 CPK21 CPK23 ABI1 RBOHD* |
| 2.52E-06 | 4 | 122.9 | Calcium-dependent protein serine/threonine kinase activity | *CDPK2 CPK21 CPK23 OST1* |
| 0.003375 | 4 | 9.314 | Zinc ion binding | *FHY3 FAR1 HD1 HDA6* |
| 5.51E-05 | 3 | 92.15 | Calmodulin-dependent protein kinase activity | *CDPK2 CPK21 CPK23* |
| 0.003375 | 3 | 15.69 | Oxidoreductase activity | *AAO3 CYP707A1 CYP707A3* |
| 0.004149 | 3 | 14.34 | DNA-binding transcription factor activity-RNA polymerase II-specific | *HDG11 bZIP23 MYBR1* |
| 9.32E-05 | 2 | 360.9 | Protein phosphatase binding | *CPK21 CPK23* |
| 9.32E-05 | 2 | 360.9 | Inositol 3-alpha-galactosyltransferase activity | *GolS2 GolS1* |
| 0.000199 | 2 | 240.6 | NAD-dependent histone deacetylase activity (H3-K14 specific) | *HD1 HDA6* |
| 0.001479 | 2 | 80.21 | Water channel activity | *PIP2A PIP1A* |
| 0.001973 | 2 | 67.15 | Protein serine/threonine phosphatase activity | *ABI1 ABI2* |
| 0.00221 | 2 | 61.43 | Kinase binding | *MYC2 ABI1* |
| 0.003375 | 2 | 44.42 | Peroxidase activity | *GPX3 APX2* |
| 0.003375 | 2 | 47.33 | Ubiquitin protein ligase binding | *AAO3 PIP2A* |
| 0.004878 | 2 | 34.37 | Oxidoreductase activity | *CYP707A1 CYP707A3* |

| **Supplementary Table 14.** Genes grouped by functional categories defined by high-level GO terms | |
| --- | --- |
| High level GO category | Genes |
| Regulation of biological process | bZIP1 bZIP23 NAC1 DREB1C CPK21 DREB1A |
| Biological regulation | bZIP1 bZIP23 NAC1 DREB1C CPK21 DREB1A |
| Regulation of cellular process | bZIP1 bZIP23 NAC1 DREB1C CPK21 DREB1A |
| Response to stimulus | PIP2-2 PIP1-1 bZIP23 PIP1-1 NAC1 CPK21 |
| Response to stress | PIP2-2 PIP1-1 bZIP23 PIP1-1 NAC1 CPK21 |
| Biosynthetic process | bZIP1 bZIP23 NAC1 DREB1C CPK21 DREB1A |
| Response to abiotic stimulus | PIP2-2 PIP1-1 bZIP23 PIP1-1 NAC1 CPK21 |
| Regulation of metabolic process | bZIP1 bZIP23 NAC1 DREB1C DREB1A |
| Response to chemical | PIP2-2 PIP1-1 bZIP23 PIP1-1 NAC1 CPK21 |
| Localization | PIP2-2 PIP1-1 PIP1-3 |
| Establishment of localization | PIP2-2 PIP1-1 PIP1-3 |
| Positive regulation of biological process | bZIP1 bZIP23 NAC1 |
| Signaling | bZIP23 CPK21 |
| Response to endogenous stimulus | bZIP23 CPK21 |
| Regulation of response to stimulus | NAC1 CPK21 |
| Cellular response to stimulus | bZIP23 CPK21 |
| Multicellular organismal process | WOX9 |
| Developmental process | WOX9 |
| Methylation | DRM3 |
| Negative regulation of biological process | CPK21 |
| Anatomical structure development | WOX9 |
